# A Systematic Survey of the Reactivity of Chlorinated N_2_P_2_, NP_3_ and P_4_ Ring Systems

**DOI:** 10.1002/chem.201903410

**Published:** 2019-11-22

**Authors:** Jonas Bresien, Liesa Eickhoff, Axel Schulz, Tim Suhrbier, Alexander Villinger

**Affiliations:** ^1^ Institut für Chemie Universität Rostock Albert-Einstein-Str. 3a 18059 Rostock Germany; ^2^ Abteilung Materialdesign Leibniz-Institut für Katalyse an der, Universität Rostock e.V. Albert-Einstein-Str. 29a 18059 Rostock Germany

**Keywords:** biradicals, computational chemistry, phosphorus, PN chemistry, ring systems

## Abstract

The reactivity of the four‐membered NP_3_ ring system [RN(μ‐PCl)_2_PR] (R=Mes*=2,4,6‐tri‐*tert‐*butylphenyl) towards Lewis acids, Lewis bases, and reducing agents was investigated. Comparisons with the literature‐known, analogous cyclic compounds [ClP(μ‐NR)]_2_ (R=Ter=2,6‐dimesitylphenyl) and [ClP(μ‐PR)]_2_ (R=Mes*) are drawn, to obtain a better systematic understanding of the reactivity of cyclic NP species. Apart from experimental results, DFT computations are discussed to further the insight into bonding and electronic structure of these compounds.

## Graphical abstract

**News on NP_3_ ring systems**: The reactivity of chlorinated NP_3_ ring systems was investigated by experimental and computational methods. Comparisons with known N_2_P_2_ and P_4_ congeners are drawn to obtain a systematic understanding of the reactivity of these types of compounds.

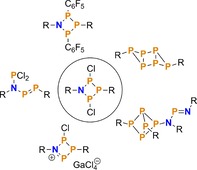

## Introduction

Phosphorus chemistry nowadays plays an important role in a variety of research domains, such as biochemistry, organic and inorganic chemistry, catalysis, and materials science.^1^, ^2^, ^3^, ^4^, ^5^, ^6^ A great variety of chemical processes rely on phosphorus compounds, both in nature^7^ and in industrial chemistry. For example, phosphane‐based ligands find widespread use in transition metal complexes that are being applied for large‐scale industrial processes, such as hydroformylation reactions or syntheses involving C−C and C−N bond formation.^3^

It is therefore desirable to further the systematic development of phosphorus chemistry, particularly when regarding the fact that phosphorus is often dubbed a “carbon copy”.^8^ The chemistry of carbon is, of course, well and systematically investigated, and there is a plethora of different types of reactions that can be used to synthesize a large variety of different classes of organic compounds. It is probably without dispute that the same level of understanding has not yet been reached in case of phosphorus chemistry, which is why we are interested in a systematic investigation of phosphorus‐based compounds.

**Scheme 1:**
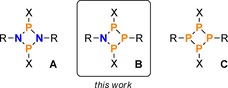
Four‐membered N_2_P_2_, NP_3_ and P_4_ ring systems (R=sterically demanding substituent, X=(pseudo)halogen).

**Scheme 2:**
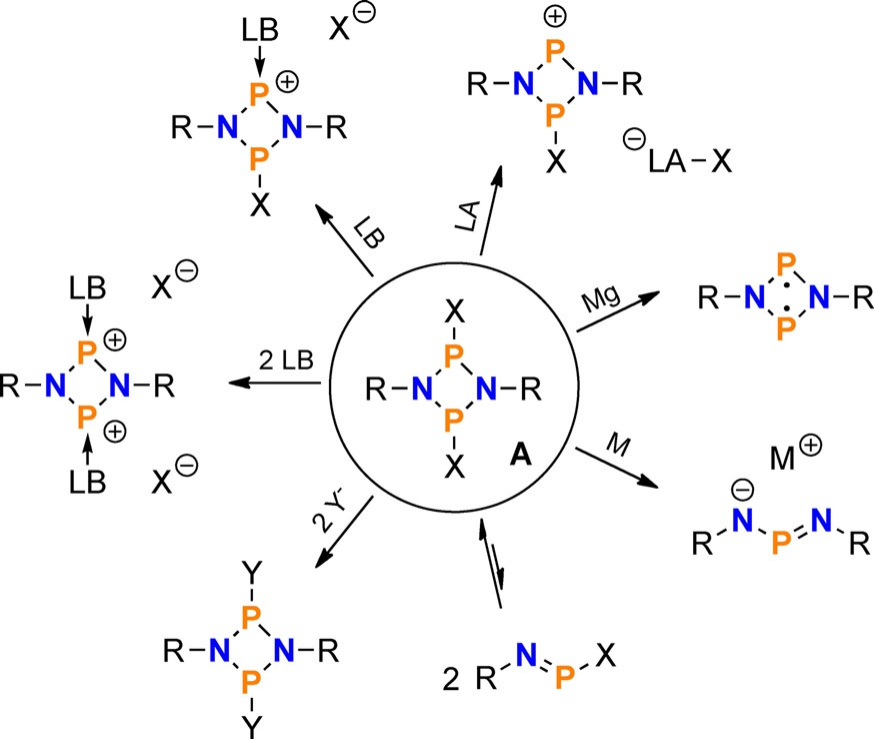
Reactivity of **A** (R=sterically demanding substituent, LB=Lewis base; LA=Lewis acid; M=Mg, K; X, Y=Cl, OTf, N_3_, etc.).

In particular, our group has a long‐standing interest in cyclic oligophosphanes and aminophosphanes. Such ring systems show a diverse reaction behaviour and have therefore attracted the interest of many researchers during the past few decades.^9^, ^10^, ^11^, ^12^, ^13^, ^14^, ^15^ Among these classes of compounds, especially four‐membered N_2_P_2_ ring systems were thoroughly studied, as detailed in a number of review articles.^16^, ^17^, ^18^, ^19^, ^20^ Out of the many possible substitution patterns at the N_2_P_2_ ring system, those species with halogen substituents (e.g. compounds of the type [XP(μ‐NR)]_2_ (**A**, X=halogen, R=sterically demanding group; Scheme 1) were shown to be easily functionalized by halide abstraction, substitution reactions, or reduction, rendering them worthwhile building blocks in phosphorus‐nitrogen chemistry (Scheme 2).^21^, ^22^, ^23^, ^24^, ^25^, ^26^

**Scheme 3:**
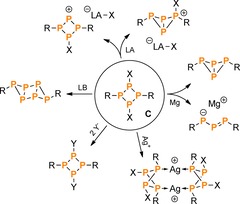
Reactivity of **C** (R=sterically demanding substituent, LB=Lewis base; LA=Lewis acid; X=Cl; Y=C_6_F_5_, see below).

More recently, we became interested in the reactivity of cyclic phosphanes of the type [XP(μ‐PR)]_2_ (**C**),^27^, ^28^, ^29^, ^30^, ^31^ which had barely been investigated prior to our work. In particular, we were interested in how the reaction behaviour of these species would compare to the congeneric N_2_P_2_ ring systems (**A**), in view of the formal replacement of the two N atoms by phosphorus. It was found that the P_4_ ring system **C** displayed a tendency to stabilize positive charges (induced by halide abstraction or substitution) by rearrangement reactions associated with the formation of transannular P−P bonds (Scheme 3).^32^, ^33^, ^34^, ^35^ The N_2_P_2_ ring system **A**, on the other hand, was shown to stabilize positive charges by delocalization of π‐electron density within the ring, due to the *p*‐type character of the lone pair of electrons (LP) at N. In contrast, the LP at phosphorus in the P_4_ ring system has a large s character, which hampers this kind of electronic interaction.

**Scheme 4:**
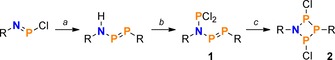
Synthesis of **2** (R=Mes*) starting from Mes*NPCl^37^ (a: Mes*PH_2_, NEt_3_; b: *n*BuLi, PCl_3_, −80 °C; c: THF).

The apparent differences in reactivity prompted us to investigate NP_3_ ring systems (**B**, Scheme 1),^36^ which incorporate only one nitrogen atom for electronic stabilization and can formally be regarded as a blend of N_2_P_2_ and P_4_ ring systems. Particularly, we were interested in the reactivity of ring system **B** towards Lewis acids, Lewis bases, nucleophiles, as well as reducing agents. The synthesis of the NP_3_ ring system [Mes*N(μ‐PCl)_2_PMes*] (**2**; Mes*=2,4,6‐tri‐*tert‐*butylphenyl, Scheme 4) was recently published^36^ and served as a starting point for the investigations reported in this paper.

## Results and Discussion

To begin with, the synthesis of **2** could be optimized using a MeCN/CH_2_Cl_2_ mixture instead of THF for the isomerization step (Scheme 4 c). As reported previously, the isomerization of **1** can lead to different products depending on the polarity of the solvent.^36^ Since more polar media favour the formation of the desired ring system **2**, MeCN seemed a reasonable choice of solvent; however, **1** and **2** were only sparingly soluble in MeCN. After addition of CH_2_Cl_2_ to the suspension, the starting material slowly dissolved. Letting the mixture rest overnight afforded large block‐shaped crystals of the product in 73 % yield. A series of ^31^P NMR spectra was recorded to monitor the progress of the isomerization. The spectra show that almost no side products were formed in the MeCN/CH_2_Cl_2_ mixture (Figure S3, Supporting Information). Using this improved protocol, compound **2** could be synthesized on multi‐gram scale.

**Scheme 5:**
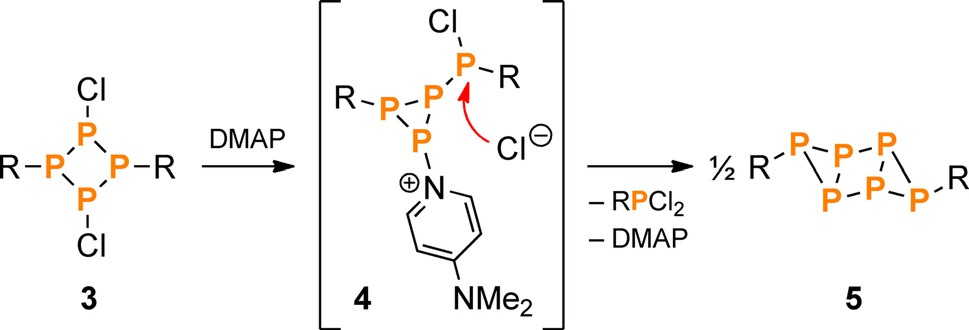
Reaction of **3** with DMAP (R=Mes*).^33^

**Scheme 6:**
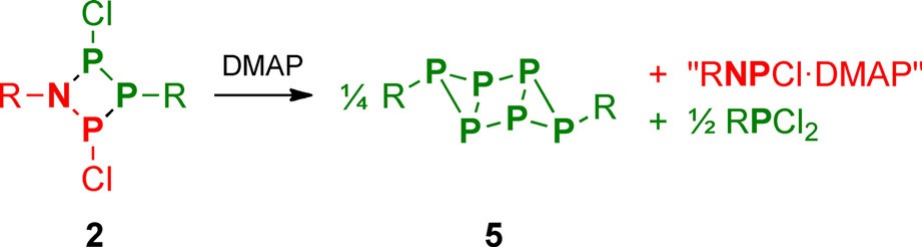
Reaction of **2** with DMAP (R=Mes*).

In a first series of experiments, the reactivity of the NP_3_ ring system **2** towards the Lewis base DMAP (4‐dimethylaminopyridine) was investigated. As previously shown, the reaction of DMAP with the P_4_ ring system [ClP(μ‐PMes*)]_2_ (**3**, type **C**) led to rearrangement of the P−P bonding system and elimination of Mes*PCl_2_, yielding the tricyclic hexaphosphane **5** in nearly quantitative yields (Scheme 5).^33^ It was therefore of interest to see if a similar reaction behaviour could be observed in case of the NP_3_ ring system **2**, possibly yielding an analogous tricyclic structure with an N‐capped P_4_ ring system. Astonishingly, though, the isolated product of the reaction of **2** with DMAP was the same tricyclic P_6_ system (**5**), indicating that the N atom had formally been eliminated from the starting material (Scheme 6). To shed light on the reaction path, in situ ^31^P NMR spectra were collected, which showed that Mes*PCl_2_ and presumably Mes*NPCl⋅DMAP (pp. S19ff) were formed as further products of the reaction. Hence, the formation of **5** can be understood in terms of a formal cycloreversion of **2** (Scheme 6), that is, the products can formally be derived from the monomeric building blocks of **2**, Mes*NPCl and “Mes*PPCl” (which dimerizes to [ClP(μ‐PMes*)]_2_ (**3**), cf. Scheme 5). However, as no intermediates besides **4** could be observed in the ^31^P NMR spectra (Figure S6), the exact mechanism of the reaction remains unclear.

It is worthy to note that the reaction of the acyclic NP_3_ compound **1** with DMAP led to a similar outcome, which indicates conversion of **1** to **2** under reaction conditions.

**Scheme 7:**
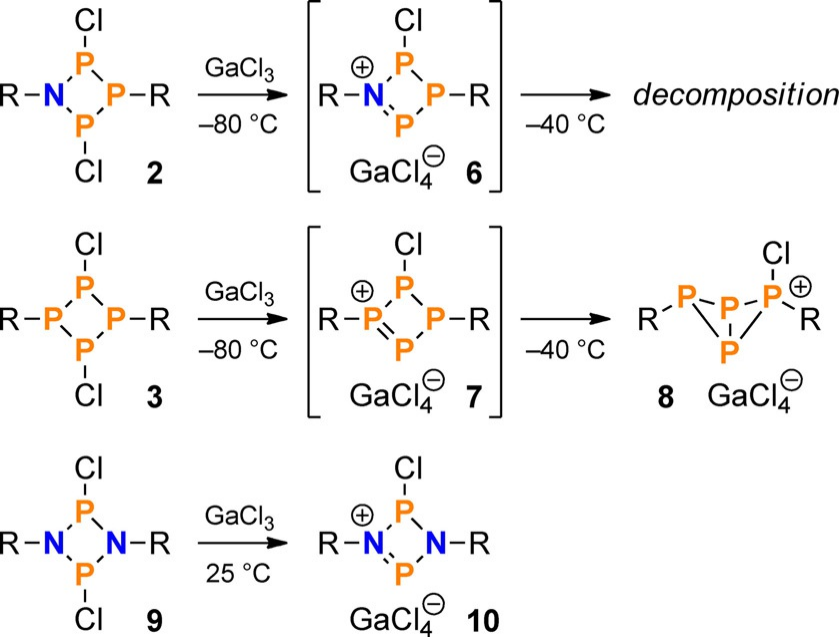
Reaction of NP_3_ ring system **2** with GaCl_3_ (R=Mes*), in comparison with the reactivities of P_4_ and N_2_P_2_ ring systems **3** (R=Mes*)^32^ and **9** (R=Ter=2,6‐dimesitylphenyl).^23^

In a next set of experiments, the reactivity of the NP_3_ ring system **2** towards Lewis acids was investigated. As GaCl_3_ had proved to be a suitable reagent for selective chloride abstraction,^38^, ^39^, ^40^, ^41^, ^42^, ^43^, ^44^ it was chosen as a model substrate to investigate this type of reaction. As expected, low temperature ^31^P NMR spectroscopy revealed that abstraction of one chloride ion from the ring system led to the formation of a phosphenium salt (**6**; Scheme 7, top), which is analogous to those observed in case of the congeneric N_2_P_2_ and P_4_ ring systems (**7**, **10**; Scheme 7).

**Figure 1:**
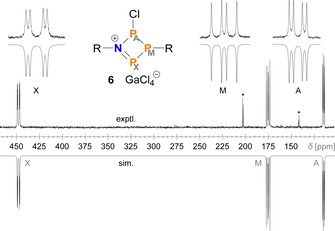
Experimental and simulated ^31^P NMR spectrum (−70 °C) of the reaction between **2** and GaCl_3_ showing the formation of phosphenium salt **6** (R=Mes*; starting material and by‐products indicated by asterisks).

Similarly to tetraphosphenium salt **7**,^32^ a solution of the azatriphosphenium salt **6** was only stable at low temperatures below −40 °C, as evidenced by variable temperature NMR spectra (Figure S8). In the ^31^P NMR spectrum, **6** was identified by an AMX spin system (Figure 1) with a characteristic, downfield‐shifted X part, due to the positive charge and strongly polarized NP double bond (*δ*(P_X_)=+446.4 ppm; cf. **7**: +358.9,^32^ **10**: +366.6 ppm).^23^ All observed NMR shifts and coupling constants are in good agreement with theoretical data (Table 1).

**Table 1:** Experimental ^31^P NMR data of **6** (AMX spin system). Calculated values (PBE0‐D3/def2‐SVP, cf. SI) are given in brackets.

	*δ*	*J* [Hz]	
	[ppm]	A	M
A	114.1 (105.7)		
M	175.4 (126.3)	−317 (−273)	
X	446.4 (442.7)	+107 (+69)	−470 (−442)

**Scheme 8:**
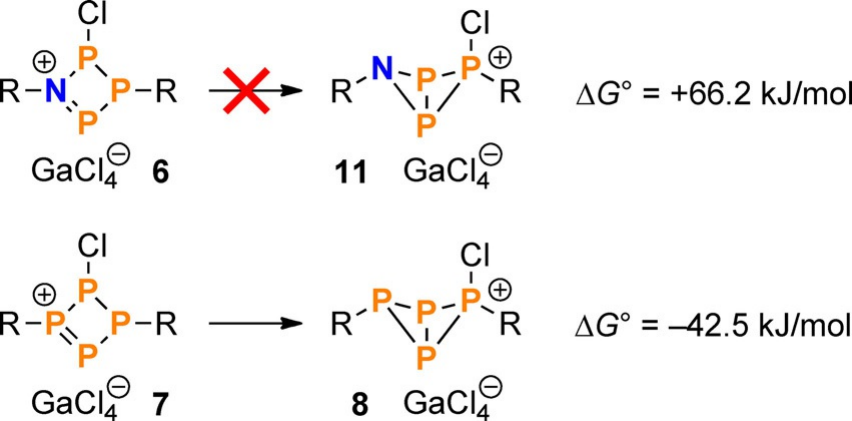
Calculated Gibbs energies for the isomerization of the cations of compounds **6** and **7** (PBE0‐D3/def2‐SVP).

Contrary to its N_2_P_2_ and P_4_ congeners **7** and **10**, the NP_3_ species **6** decomposed to an unidentified mixture of products upon warming. At first glance, this might seem unexpected, especially in view of the fact that the diazadiphosphenium salt **10** is perfectly stable up to well beyond 200 °C.^23^ In case of the NP_3_ species **6**, the single N atom is apparently not sufficient to stabilize the formal phosphenium centre by donation of π‐electron density into the vacant *p*‐orbital at P. On the other hand, while the P_4_ system **7** can stabilize itself by formation of a transannular P−P bond and concomitant pyramidalization of all P atoms, the same is not true of the NP_3_ ring system **6**, as this would entail a high ring strain at the N atom, which is energetically unfavourable.^45^, ^46^ Consequently, the bicyclic NP_3_ isomer **11** is calculated to be much higher in energy than the phosphenium isomer **6** (Scheme 8), whereas the isomerization of the P_4_ system **7** to the bicyclic phosphino‐phosphonium salt **8** is thermodynamically favoured.

**Scheme 9:**
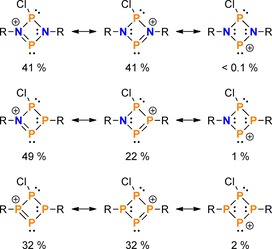
Some Lewis resonance structures of model phosphenium cations (R=H) as well as their respective weights from NRT analysis.

To better understand the differences in bonding within the cations of **6**, **7**, and **10**, Natural Resonance Theory (NRT) calculations on model cations (with R=H) were performed. For each species, a total of about 45 resonance structures was considered, most of which describe bond polarization within the ring or negative hyperconjugation of the LPs at Cl into the ring system. To simplify the discussion, only the two most important contributions to the Lewis resonance Scheme, as well as the Lewis structure with an electron sextet at the formal phosphenium centre will be considered in the following (Scheme 9). Two trends can be derived, which underline the observed reactivity: Firstly, the overall contribution of the two most important Lewis structures (with NP or PP double bonds, respectively) decreases along the series N_2_P_2_, NP_3_, P_4_; that is the formal phosphenium is best stabilized by π‐type interactions in the N_2_P_2_ cation, and least stabilized in the P_4_ derivative. Secondly, the weight of the resonance structure with an electron sextet at the formal phosphenium increases from N_2_P_2_ to P_4_, formally rendering the latter the most reactive derivative. This is corroborated by the fact that the isomerization of the tetraphosphenium salt **7** to the bicyclic isomer **8** proceeded even at −80 °C.^32^

**Figure 2:**
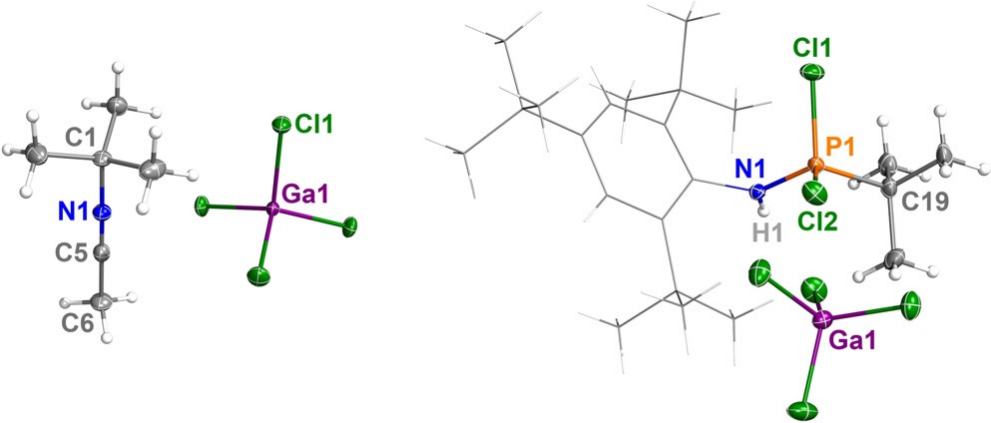
Molecular structures of the decomposition products [MeCN*t*Bu]. [GaCl_4_] (left) and [Mes*N(H)PCl_2_ *t*Bu][GaCl_4_] (right) in the crystal. Ellipsoids are set at 50 % probability (123 K). Selected bond lengths (Å) and angles (°): [CH_3_CN*t*Bu][GaCl_4_] N1−C1 1.479(3), N1−C5 1.129(3), C5‐N1‐C1 178.2(2), N1‐C5‐C6 180.0(3), C1‐N1‐C5‐C6 180.0; [Mes*N(H)PCl_2_ *t*Bu][GaCl_4_] N1−P1 1.604(2), N1−H1 0.77(3), P1−C19 1.815(3), P1−Cl1 1.9798(9), N1‐P1‐C19 112.1(1), N1‐P1‐Cl1 111.40(9), C19‐P1‐Cl1 109.8(1), C1‐N1‐P1‐C19 −179.4(2).

As with the P_4_ congener **7**, all attempts to crystallize the azatriphosphenium salt **6** at low temperatures remained unsuccessful. In two instances, a few crystals of two different decomposition products could be isolated and studied by single crystal X‐ray diffraction (SC‐XRD). In both cases, a *t*Bu group of the Mes* moiety had been transferred onto another molecular fragment or solvent molecule, indicating decomposition of the sterically demanding substituent (Figure 2). Cleavage of *t*Bu groups from Mes* substituents is not uncommon in the presence of Lewis acids (or by thermal treatment),^47^, ^48^, ^49^, ^50^, ^51^ thus demonstrating the high Lewis acidity of salt **6**.

**Scheme 10:**
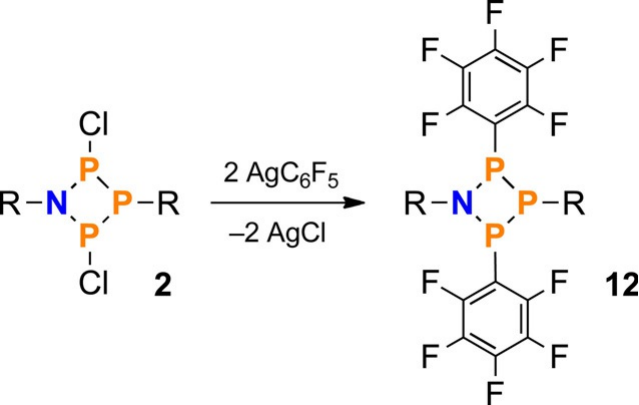
Reaction of **2** with AgC_6_F_5_ (R=Mes*). Analogous reactions were observed for congeneric N_2_P_2_ 52 and P_4_ species.

**Figure 3:**
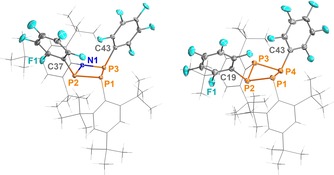
Molecular structures of **12** (left) and **13** (right) in the crystal. Ellipsoids are set at 50 % probability (123 K and 173 K, respectively). Selected bond lengths (Å) and angles (°): **12** P1−P2 2.2461(6), P1−P3 2.2532(6), P2−N1 1.744(1), P2−C37 1.884(2), P3−N1 1.742(1), P3−C43 1.888(2), P2‐P1‐P3 76.09(2), N1‐P2‐P1 88.29(5), N1‐P3‐P1 88.12(5), P3‐N1‐P2 105.39(7), P1‐P2‐P3‐N1 −164.04(8); **13** P1−P2 2.2471(8), P1−P4 2.2611(9), P2−C19 1.853(2), P2−P3 2.1949(9), P3−P4 2.2367(8), P4−C43 1.858(2), P2‐P1‐P4 84.37(3), P3‐P2‐P1 88.99(3), P2‐P3‐P4 86.17(3), P3‐P4‐P1 87.61(3), P1‐P2‐P4‐P3 −142.45(4).

Next, we were interested in demonstrating nucleophilic substitution at the NP_3_ ring system **2**. By analogy with the congeneric N_2_P_2_ ring system [ClP(μ‐NDipp)]_2_ (Dipp=diisopropylphenyl),^52^ the reaction of **2** with AgC_6_F_5_ resulted in precipitation of AgCl and formation of [Mes*N(μ‐PC_6_F_5_)_2_PMes*] (**12**, Scheme 10). After filtration, the latter could be crystallized from CH_2_Cl_2_/MeCN to give yellow, block shaped crystals suitable for SC‐XRD (Figure 3, left; yield of isolated substance: 48 %).

The molecular structure of **12** revealed a flattened NP_3_ ring system (fold angle:^29^, ^53^ 164.04(8)°) with NP and PP bond lengths that correspond to somewhat shortened NP and slightly elongated PP single bonds, respectively (cf. Σ*r*_cov_(N−P)=1.82 Å;. Σ*r*_cov_(P−P)=2.22 Å).^54^ The nitrogen atom is located in a nearly planar coordination environment (Σ(∢N)=352.8(3)°), whereas the phosphorus atom P1 is strongly pyramidalized (Σ(∢P)=277.2(1)°). These structural parameters compare well with those of the starting material (**2**).^36^

Since the reactivity of the P_4_ ring system **3** towards AgC_6_F_5_ had not been reported previously, we also treated a solution of **3** with AgC_6_F_5_, resulting in the corresponding P_4_ ring system [F_5_C_6_P(μ‐PMes*)]_2_ (**13**, Figure 3, right). Its crystal structure could be determined by SC‐XRD. While the overall molecular structure is quite similar to that of its NP_3_ congener (**12**), compound **13** exhibits two Mes*‐bound P atoms in a pyramidal coordination environment. Hence, the P_4_ ring system adopts a much more puckered conformation with a fold angle of 142.45(4)°, which corresponds nicely to the experimental fold angle of the P_4_ ring system in the starting material **3** (120–143° depending on the modification).^29^

**Scheme 11:**
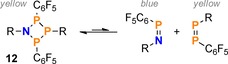
Cycloreversion of **12** in solution (R=Mes*). Due to the blue colour of (*Z*)‐Mes*N=PC_6_F_5_, the mixture appears green. The equilibrium ratio between the ring system **12** and the cycloreversion products is about 5:1:1.

Both **12** and **13** displayed complex heteronuclear coupling patterns in the ^31^P and ^19^F NMR spectra. Additionally, the NP_3_ ring system **12** exhibited various broadened signals due to hindered rotation of the C_6_F_5_ substituents (Figure S9). It is worthy to note that a yellow solution of **12** in CH_2_Cl_2_ started to turn green after one day, which was accompanied by the appearance of additional signals in the ^31^P NMR spectrum. These signals could be assigned to the [2+2] cycloreversion products of the NP_3_ ring system **12**, that is, (*Z*)‐Mes*P=PC_6_F_5_ (*δ*(^31^P)=381.2, 568.7 ppm; *J*=−557 Hz) and (*Z*)‐Mes*N=PC_6_F_5_ (*δ*(^31^P)=361.7 ppm)^52^ (Scheme 11, see also Figure S10). Computed ^31^P NMR data corroborate the assignment ((*Z*)‐Mes*P=PC_6_F_5_: 373.7, 525.8 ppm, −507 Hz; (*Z*)‐Mes*N=PC_6_F_5_: 372.8 ppm). The same type of reactivity was previously discussed for “symmetric” N_2_E_2_ (E=pnictogen) ring systems that can be regarded as dimers of iminopnictanes (RN=ER′).^21^, ^40^, ^55^ The equilibrium between monomeric and dimeric species was shown to depend on the size of the substituents R and R′.

**Scheme 12:**
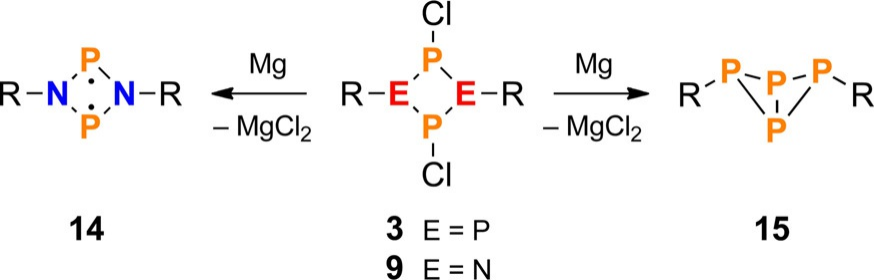
Reduction of N_2_P_2_ (**9**) and P_4_ ring systems (**3**) leads to different types of products, namely the open shell singlet biradical **14** in case of E=N, and the closed shell [1.1.0]‐bicyclic species **15** in case of E=P.

Lastly, the reduction of the NP_3_ ring system **2** was of particular interest, especially when considering that the congeneric N_2_P_2_ species **9** and P_4_ species **3** yielded two very different reduction products (Scheme 12). While the former can be reduced to a singlet biradical without a transannular PP bond (**14**),^26^ reduction of the latter leads selectively to the formation of a P_4_ butterfly with a transannular PP bond (**15**).^34^ This can be understood in terms of π‐electron delocalization, as detailed above, or ring strain at the nitrogen atom which prevents the N_2_P_2_ species from pyramidalization.^46^ In any case, it seemed interesting to explore which type of stabilization (planarization vs. pyramidalization) would predominate in the NP_3_ case.

**Scheme 13:**
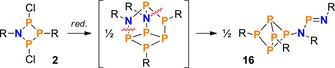
Formally, cage compound **16** (R=Mes*) can be derived from a β‐cage‐type structure, which itself may be viewed as a dimer of Mes*NP_3_Mes* (red.=reduction).

**Scheme 14:**
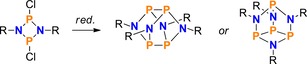
With small substituents (e.g. R=*t*Bu), the reduction of N_2_P_2_ ring systems leads to the formation of N_4_P_4_ cage compounds (left: α‐, right: β‐cage) that can be regarded as dimers of the respective biradicals [P(μ‐NR)]_2_.^59^, ^60^, ^61^

To that end, compound **2** was reduced using activated Mg chips, by analogy with the reduction of **3** and **9**.^26^, ^34^, ^56^ This led to a mixture of products, which—to our surprise—contained the bicyclic tetraphosphane Mes*P_4_Mes* (**15**)^57^ as one of the main components, indicating a formal cycloreversion of the NP_3_ ring system into NP and PP fragments. To further investigate the formation of the bicyclotetraphosphane **15**, Cp_2_Ti(BTMSA)^58^ (BTMSA=bis(trimethylsilyl)acetylene) was employed as a milder reducing agent. Indeed, this procedure facilitated the isolation of a few crystals of an unusual N_2_P_6_ cage compound with a bicyclo[1.1.1]pentaphosphane scaffold (**16**, Scheme 13). Compound **16** may be regarded as a dimer of the biradical Mes*NP_3_Mes* (**17**, p. S62f), which is predicted to be only slightly less stable than its [1.1.0]‐bicyclic isomer (**18**, Δ*G*°=14.2 kJ mol^−1^ at DLPNO‐CCSD(T)/def2‐TZVP//PBE‐D3/def2‐SVP level of theory, see below). Formal dimerization of congeneric N_2_P_2_ biradicals was already reported for species with small substituents R, leading to the formation of α‐ or β‐cage structures with an N_4_P_4_ scaffold (Scheme 14).^59^, ^60^, ^61^

**Figure 4:**
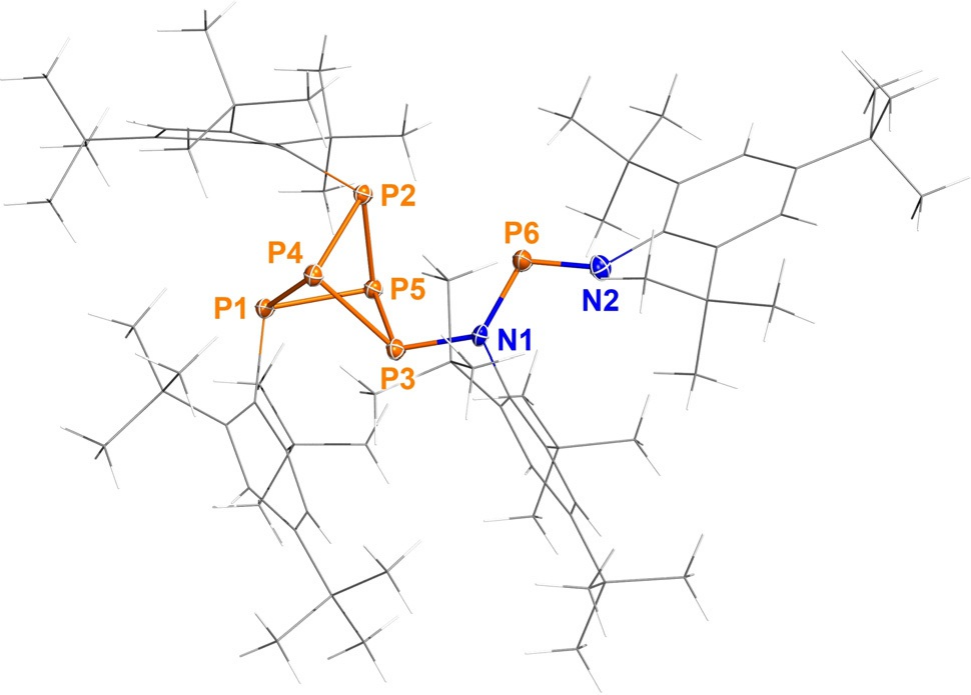
Molecular structure of **16** in the crystal. Ellipsoids are set at 50 % probability (123 K). Selected bond lengths (Å) and angles (°): P1−P4 2.245(1), P1−P5 2.274(1), P2−P4 2.250(1), P2−P5 2.276(1), P3−P4 2.270(1), P3−P5 2.225(1), P3−N1 1.759(3), P6−N1 1.702(3), P6−N2 1.544(3), P4‐P1‐P5 77.66(4), P4‐P2‐P5 77.50(4), P5‐P3‐P4 78.14(4), P1‐P4‐P2 84.80(4), P1‐P4‐P3 81.23(4), P2‐P4‐P3 88.27(5), P3‐P5‐P1 81.58(4), P3‐P5‐P2 88.72(5), P1‐P5‐P2 83.52(4), N1‐P3‐P4 116.2(1), N1‐P3‐P5 112.2(1), P6‐N1‐P3 121.4(2), N2‐P6‐N1 110.6(2), P1‐P4‐P5‐P2 118.59(6), P1‐P4‐P5‐P3 −113.97(6), P2‐P4‐P5‐P3 127.44(6), P3‐N1‐P6‐N2 −167.9(2).

The molecular structure of **16** could be elucidated by SC‐XRD (Figure 4). All PP bond lengths within the bicyclo[1.1.1]pentaphosphane scaffold correspond to slightly elongated PP single bonds (cf. Σ*r*_cov_(P−P)=2.22 Å).^54^ The N1−P6 distance corresponds to a polarized NP single bond, whereas the N2‐P6 distance is in the range of a typical NP double bond (cf. Σ*r*_cov_(N−P)=1.82, Σ*r*_cov_(N=P)=1.62 Å). It is worthy to note that the bond angles at the P atoms within the P_5_ cage are quite small (P1, P2, P3: avg. 77.8(2)°; P4, P5: avg. 84(1)°), as expected for a polycyclic P_*n*_ structure. When viewed along the P4−P5 axis, all substituents are bent to the left, thus minimizing the repulsion between the LPs at P1, P2, and P3. To the best of our knowledge, compound **16** is the first example of a bicyclo[1.1.1]pentaphosphane derivative.

In our attempts to fully characterize compound **16**, it transpired that this species was rather unstable and only of intermediary nature. Several NMR experiments were run using isolated crystals of compound **16** as well as the reaction mixture of **2** and Cp_2_Ti(BTMSA) (Figure S13). All experiments eventually indicated the formation of a similar product mixture as observed for the reduction of **2** with Mg. Hence, the formation of Mes*P_4_Mes* can be rationalized by formal cleavage of the P3‐P4 and P3‐P5 bonds in compound **16**.

**Figure 5:**
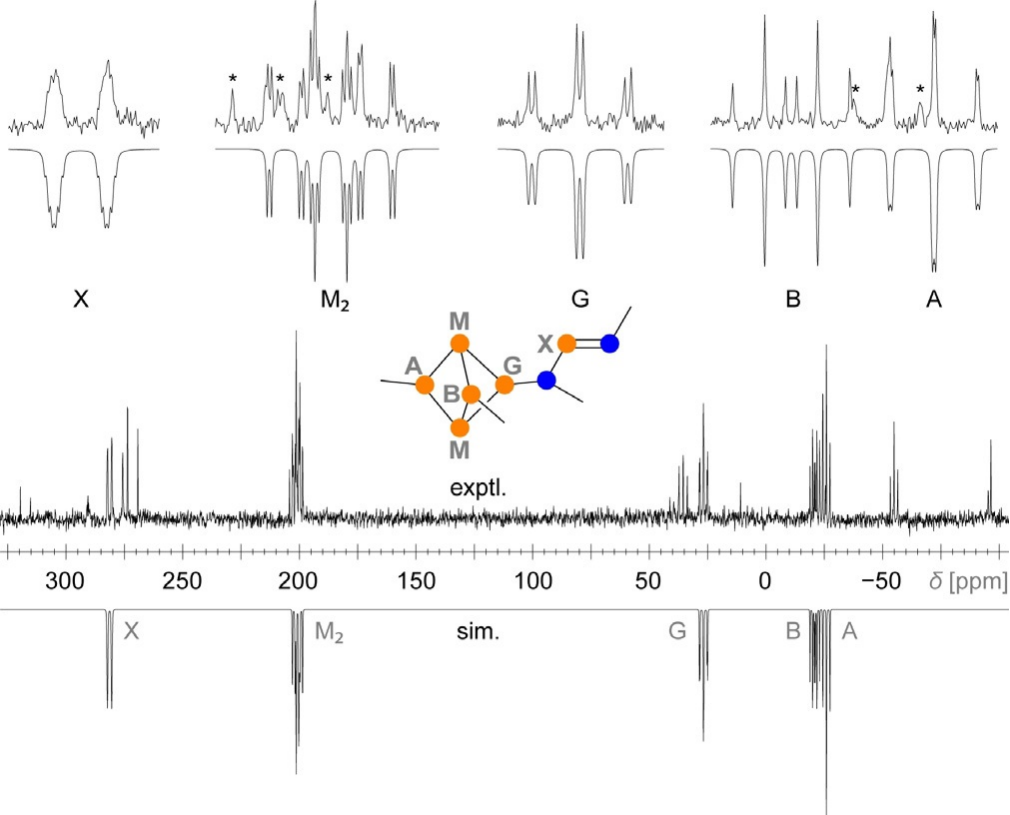
^31^P NMR spectrum of a solution containing cage compound **16**. The experimental spectrum (up) shows some impurities due to the instability of **16**. The simulated spectrum (down) was fitted to the most intense signals. A detailed view of all signals is presented at the top (lines caused by impurities are indicated by an asterisk).

Despite its inherent instability, compound **16** could be identified by ^31^P NMR spectroscopy in a solution that was freshly prepared from isolated crystals. The six phosphorus atoms displayed an ABGM_2_X spin system (Figure 5), with typical NMR shifts for the Mes*‐substituted P atoms (−26.0, −21.0 ppm)^29^ and a downfield‐shifted X part corresponding to the P atom involved in the NP double bond (281.3 ppm). The experimental shifts and coupling constants, which were extracted from the spectrum by line‐shape fitting, correspond very well to calculated NMR data (Table 2).

**Table 2:** Experimental ^31^P NMR data of **16** (ABGM_2_X spin system). Calculated values (PBE0‐D3/def2‐TZVP, cf. SI) are given in brackets.

	*δ*	*J* [Hz]			
	[ppm]	A	B	G	M
A	−25.0 (−19.0)				
B	−21.0 (−26.6)	−4 (−14)			
G	26.7 (29.9)	−6 (−22)	−3 (−9)		
M_2_	200.7 (189.9)	−185 (−132)	−136 (−83)	−202 (−160)	
X	281.3 (283.7)	−14 (−15)	+224 (+288)	−28 (−36)	+19 (+47)

Natural bond orbital (NBO) and natural localized molecular orbital (NLMO) analysis^62^, ^63^ of **16** revealed that all P atoms of the P_5_ scaffold are connected by localized σ‐type bonds, and that each atom possesses one LP that is mainly localized in an s orbital (s‐character: 65–69 %). The Wiberg bond indices of the PP bonds range from 0.90 to 0.95, indicating typical PP single bonds in agreement with experimental structural data. The exocyclic NPN scaffold comprises an NP double bond (N2=P6) and a *p*‐type LP at N1, which interacts slightly with the anti‐bonding π* orbital (donor–acceptor energy *E* ^(2)^=106.6 kJ mol^−1^). Again, these findings are in line with the experimental structure.

**Scheme 15:**
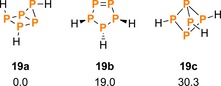
Different isomers of P_5_H_3_ and their relative Gibbs energies (Δ*G*° in kJ mol^−1^, DLPNO‐CCSD(T)/def2‐TZVP//PBE0‐D3/def2‐TZVP).

The stability of different P_5_H_*n*_ (*n=*0–7) structures has been investigated theoretically and experimentally in a number of publications.^64^, ^65^, ^66^, ^67^, ^68^, ^69^, ^70^, ^71^ Still, the bicyclo[1.1.1]pentaphosphane motif was considered only in a single publication, which identified the minimum energy structure of P_5_H_3_ as bicyclo[2.1.0]pentaphosphane (**19 a**).^68^ This is in agreement with our own results, which show that bicyclo[1.1.1]pentaphosphane (**19 c**) is in fact the least favoured isomer of those previously discussed in the literature (Scheme 15, see also p. S40).

**Scheme 16:**
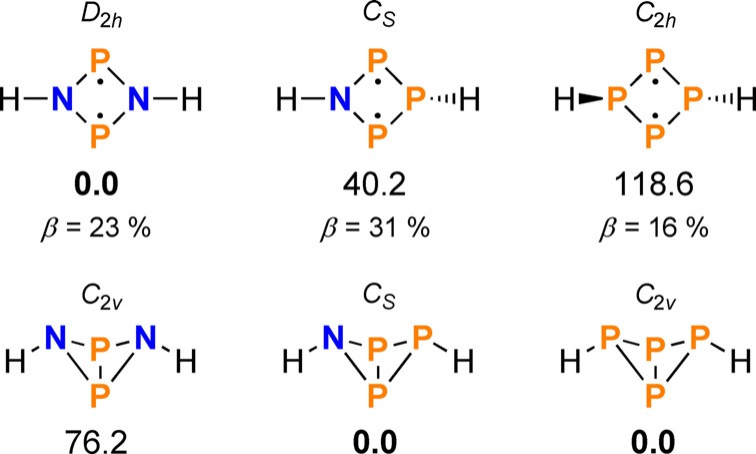
Comparison of N_2_P_2_, NP_3_ and P_4_ ring systems with respect to the relative stabilities of biradical and bicyclic isomers (Δ*G*° in kJ mol^−1^, DLPNO‐CCSD(T)/def2‐TZVP//PBE‐D3/def2‐SVP).

Since we did not find clear experimental evidence whether the reduction of the NP_3_ ring system **2** led to intermediary formation of the biradical (**17**) or bicyclic isomer (**18**) of Mes*NP_3_Mes* (cf. Supporting Information, pp. S35f and Scheme 16 for R=H), DFT and ab‐initio calculations were performed to compare both isomers (for details on computations, please refer to the Supporting Information). As already indicated, the bicyclic structure **18** is energetically slightly favoured (Δ*G*°=14.2 kJ mol^−1^). When disregarding effects of the bulky substituents, that is, using H_2_NP_3_ as a model compound, the difference in energy between both isomers is somewhat more pronounced (Δ*G*°=40.2 kJ mol^−1^, Scheme 16). By comparison, the energetic difference between biradical and bicyclic structure of H_2_P_4_ amounts to 118.6 kJ mol^−1^, rendering the hypothetical H_2_P_4_ biradical very unstable. In contrast, the H_2_N_2_P_2_ biradical is substantially more stable than the bicyclic structure (Δ*G*°=−76.2 kJ mol^−1^), in agreement with earlier considerations and experimental observations.^26^, ^46^

The biradical character of H_2_NP_3_ was computed to be 31 %, which is slightly larger than the biradical character of its N_2_P_2_ congener (23 %; cf. [P(μ‐NTer)]_2_ (**14**): 27 %).^72^ The predicted biradical character of the P_4_ species is still smaller (16 %), which can be attributed to a different through‐bond interaction in the absence of a N atom.^73^, ^74^ Overall, the biradical character of all three species is moderate and compares with other pnictogen‐based biradicals.^75^, ^76^, ^77^, ^78^, ^79^

## Conclusion

**Scheme 17:**
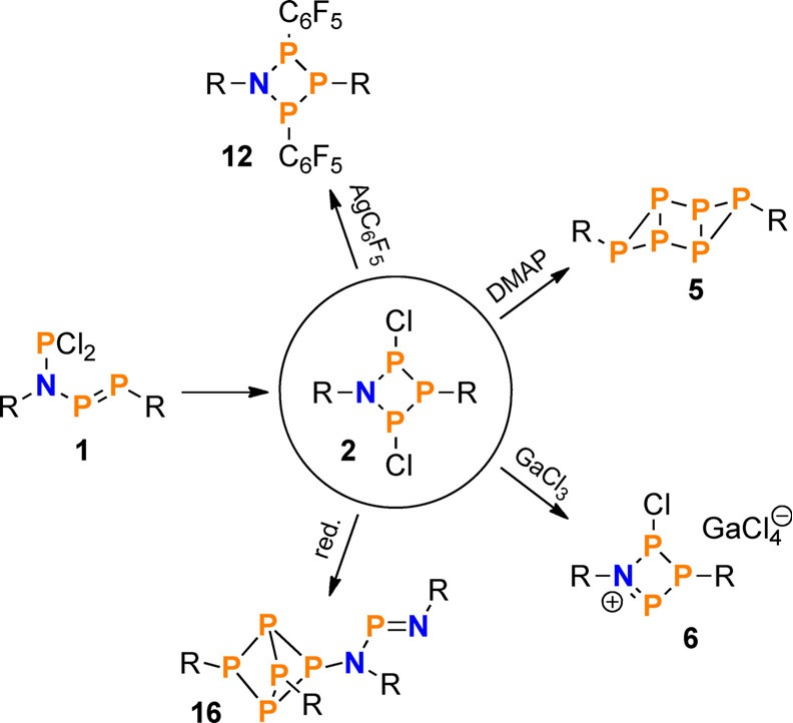
Summarized reactivity of the NP_3_ ring system **2**.

In conclusion, it was shown that the chemistry of the NP_3_ ring system **2** systematically expands the known chemistry of congeneric N_2_P_2_ and P_4_ ring systems. Some similarities with these known compounds notwithstanding, we could observe some unexpected reaction behaviour, such as the formation of a bicyclo[1.1.1]pentaphosphane derivative (**16**) or the formation of products with formal [NP]_*n*_ and [PP]_*n*_ composition due to formal cycloreversion of the NP_3_ ring system (e.g. Mes*P_6_Mes* (**5**), Mes*NPCl, or (*Z*)‐Mes*PPC_6_F_5_; cf. Scheme 17).

In particular, it was demonstrated that chloride abstraction from **2** using a Lewis acid such as GaCl_3_ resulted in the formation of a highly labile azatriphosphenium salt (**6**). Substitution of the Cl atoms with C_6_F_5_, on the other hand, gave a rather stable NP_3_ ring system (**12**), which underwent partial cycloreversion in solution to yield a diphosphene and an iminophosphane. Most intriguingly, the reduction of **2** afforded a bicyclo[1.1.1]pentaphoshane (**16**), an as yet uninvestigated substance class.

Comprehensive theoretical studies were performed to understand the differences and similarities between N_2_P_2_, NP_3_ and P_4_ ring systems. One main factor that governs the stability of the products is the electronic structure of the lone pairs of electrons at N versus P: The p‐type lone pair at N can easily delocalize into the ring system, resulting in resonance stabilization of highly reactive species, whereas the s‐type lone pair at P is rather unsuited for this kind of interaction. Moreover, the ring strain at N versus P plays a role in the stability of different ring systems, as previously detailed elsewhere.^45^, ^46^

In consequence, N_2_P_2_ derivatives tend to form planar ring systems (often involving electron delocalization), while P_4_ systems may stabilize themselves by intramolecular bond formation (bicyclic structures). The investigated reactivity of the NP_3_ ring system **2** implies that, in case none of the former types of stabilization predominate, cycloreversion becomes important as an alternative pathway of energy gain. This is often associated with a mixture of products, rendering the isolation of pure substances a challenge.

## Experimental Section

All manipulations were carried out under oxygen‐ and moisture‐free conditions in an inert atmosphere of argon, using standard Schlenk or Drybox techniques. For detailed synthetic protocols, analytic data and experimental spectra please refer to the Supporting Information.

CCDC https://www.ccdc.cam.ac.uk/services/structures?id=doi:10.1002/chem.201903410 contain the supplementary crystallographic data for this paper. These data are provided free of charge by http://www.ccdc.cam.ac.uk/.

Computations were performed using the programs Gaussian09^80^ and Orca 4.1.1.^81^ Structure optimizations employed the pure density functional PBE or hybrid functional PBE0,^82^, ^83^, ^84^ in conjunction with Grimme's dispersion correction D3(BJ).^85^, ^86^ For more accurate single‐point energies, the DLPNO‐CCSD(T)^87^, ^88^, ^89^ method was applied. All calculations used the basis sets def2‐SVP or def2‐TZVP.^90^ Detailed information on all calculations is given in the Supporting Information.

## Conflict of interest

The authors declare no conflict of interest.

## Supporting information

As a service to our authors and readers, this journal provides supporting information supplied by the authors. Such materials are peer reviewed and may be re‐organized for online delivery, but are not copy‐edited or typeset. Technical support issues arising from supporting information (other than missing files) should be addressed to the authors.

Supplementary
