# Supplementary material for: A Systematic Survey of the Reactivity of Chlorinated N2P2, NP3 and P4 Ring Systems
Source: Chemistry. 2019 Nov 22;25(71):16311–9. doi: 10.1002/chem.201903410 (PMC6973262; doi:10.1002/chem.201903410)
Supplement: Supplementary file 1 — Supplementary [file CHEM-25-16311-s001.pdf]

CHEMISTRY
A European Journal

Supporting Information

A Systematic Survey of the Reactivity of Chlorinated N_2P_2, NP_3 and P_4 Ring Systems

Jonas Bresien,*[a] Liesa Eickhoff,[a] Axel Schulz,*[a, b] Tim Suhrbier,[a] and Alexander Villinger[a]

chem_201903410_sm_miscellaneous_information.pdf

SUPPORTING INFORMATION

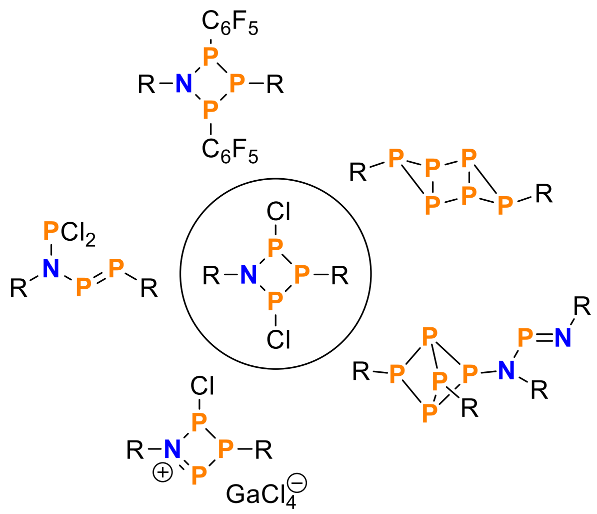

This file includes:

1 Experimental ... S2

2 Structure Elucidation ... S4

3 Syntheses of Starting Materials ... S9

4 Syntheses of Compounds ... S16

5 Additional spectroscopic data ... S37

6 Computational Details ... S38

7 References ... S69

1 Experimental

General Information. All manipulations were carried out under oxygen- and moisture-free conditions in an inert atmosphere of argon, using standard Schlenk or Drybox techniques.

All solvents were obtained from commercial sources. Reactants and starting materials were either obtained from commercial sources or were synthesized. Dichloromethane (CH_2Cl_2) was purified according to a literature procedure[1], dried over P_4O_{10}, stored over CaH_2 and freshly distilled. Prior to use, it was degassed by at least three freeze-pump-thaw cycles. Diethyl ether (Et_2O), tetrahydrofuran (THF), benzene (C_6H_6) and toluene (C_7H_8) were dried over Na/benzophenone and freshly distilled prior to use. *n*-Pentane and *n*-hexane were dried over Na/benzophenone/tetraglyme (tetraglyme = $Me(OCH_2CH_2)_4OMe$) and freshly distilled prior to use. Fluorobenzene (PhF) and acetonitrile (CH_3CN) were dried over CaH_2, freshly distilled and degassed prior to use. Deuterated dichloromethane (CD_2Cl_2) was dried over P_4O_{10}, stored over CaH_2, freshly distilled and degassed. Deuterated benzene (C_6D_6) and tetrahydrofuran (THF-d_8) were dried over Na and freshly distilled, with the latter being stored over molecular sieves (4 Å) in the refrigerator. Deuterated acetonitrile (CD_3CN) was dried over CaH_2, freshly distilled and stored over molecular sieves (4 Å).

1,3-Dichlorobenzene (abcr), 4-dimethylaminopyridine (Sigma-Aldrich), magnesium turnings (abcr, 99.8%, for Grignard reactions) and *n*-butyllithium (Acros, 2.5 mol/L in hexane) were used as received. Gallium trichloride (Sigma-Aldrich, 99.999 %) and triphenylphosphane were purified *via* sublimation. Triethylamine (Sigma-Aldrich, 99 %) was dried over Na and freshly distilled. Phosphorus trichloride (Merck, for synthesis) was dried over P_4O_{10}, freshly distilled and degassed. Mes*PH_2, Mes*NPCl, Mes*N(PCl_2)PPMes*, [ClP(μ-PMes*)]$_2$, Cp_2Ti(BTMSA) and AgC_6F_5 were prepared according to literature procedures.[2–5]

NMR spectra were obtained on a Bruker AVANCE 250, 300 or 500 MHz spectrometer and were referenced internally to the deuterated solvent (^{13}C: CD_2Cl_2 δ_{ref} = 54.0 ppm, C_6D_6 δ_{ref} = 128.4 ppm, THF-d_8 $\delta_{ref,1}$ = 25.4 ppm, $\delta_{ref,2}$ = 67.6 ppm), to protic impurities in the deuterated solvent (^{1}H: $CHDCl_2$ δ_{ref} = 5.32 ppm, C_6HD_5 δ_{ref} = 7.16 ppm, THF-d_7 $\delta_{ref,1}$ = 1.73 ppm, $\delta_{ref,2}$ = 3.58 ppm, CHD_2CN δ_{ref} = 1.95 ppm) or externally (^{31}P: 85% H_3PO_4 δ_{ref} = 0 ppm). All measurements were carried out at ambient temperature unless denoted otherwise. For NMR spectra simulation, the calculated and experimental ^{31}P NMR spectra were transferred to gNMR.[6] The full lineshape iteration procedure of gNMR was applied to match the calculated to the experimental spectrum. The signs of $^{n}J(^{31}P,^{31}P)$ coupling constants were derived from the calculated spectra (see Computational Details, p. S38).

IR spectra of crystalline samples were recorded on a Nicolet 380 FT-IR spectrometer with a Smart Orbit ATR unit at ambient temperature.

Raman spectra of crystalline samples were recorded using a LabRAM HR 800 Horiba Jobin YVON Raman spectrometer equipped with an Olympus BX40 microscope with variable lenses. The samples were excited by a red LASER (633 nm, 17 mW). All measurements were carried out at ambient temperature.

Elemental analyses were obtained using an Elementar vario Micro cube CHNS analyzer.

Melting points (uncorrected) were determined using a Stanford Research Systems EZ Melt at a heating rate of 20 °C/min.

DSC analyses were carried out at a heating rate of 5 °C/min using a Mettler-Toledo DSC 823e.

Mass spectra were recorded on a Thermo Electron MAT 95-XP using crystalline samples.

2 Structure Elucidation

X-Ray Structure Determination: X-Ray quality crystals were selected in Fomblin YR-1800 perfluoroether (Alfa Aesar) at ambient temperature. The samples were cooled to 123(2) K or 173(2) K during measurements. Data was collected on a Bruker D8 Quest diffractometer or a Bruker Kappa Apex II diffractometer using Mo K_α radiation (λ = 0.71073 Å). The structures were solved by direct methods (SHELXTL)[7] and refined by full matrix least squares procedures (SHELXL-2013).[8] Semi-empirical absorption corrections were applied (SADABS).[9] All non-hydrogen atoms were refined anisotropically, hydrogen atoms were included in the refinement at calculated positions using a riding model (with exception of H1 in [Mes*N(H)PCl_2*t*Bu][$GaCl_4$] · CH_2Cl_2).

Compound **2** crystallized as CH_2Cl_2 solvate with three independent molecules in the asymmetric unit. Three of the *t*Bu groups were found to be disordered and were split into two parts each, which were restrained to adopt similar geometries using the SAME command. Additionally, a rigid bond model (SIMU/RIGU) was applied to restrain the ADPs of all atoms within these parts. Moreover, the positions of the dichloromethane molecules were found to be only partially occupied, thus the occupation factor was freely refined. One DCM molecule was disordered over two positions, which were restrained to adopt similar geometries using the SAME command. Additionally, the ADPs of neighbouring atoms were restrained to be similar using a rigid bond restraint (SIMU/RIGU). The ADPs of the C atoms in both parts were made equal using EADP.

The solvent molecule of [Mes*N(H)PCl_2*t*Bu][$GaCl_4$] · CH_2Cl_2 was found to be highly disordered and was therefore split into four parts. All parts were restrained to adopt similar geometries using the SAME command. Moreover, SIMU/RIGU was applied to restrain the ADPs of all atoms within these parts. Additional EADP commands were applied for atom positions that were very close or nearly on top of each other.

Compound **12** crystallized with two molecules in the asymmetric unit. One *t*Bu group was found to be disordered and was split into two parts, which were restrained to adopt similar structures using the SAME/SADI commands. The ADPs of neighbouring atoms within these parts were restrained to be similar using a rigid bond restraint (SIMU/RIGU).

The co-crystallized fluorobenzene molecule of **13** · PhF was found to be disordered and was split into three parts, which were restrained to be planar (FLAT) and adopt similar geometries using the SAME command. The ADPs of corresponding C atoms in the A/B/C parts were made equal. Additionally, the displacement parameters of neighbouring atoms within the PhF molecule were restrained to be similar using a rigid bond model (SIMU/DELU).

Three *t*Bu groups in the structure of **16** were found to be disordered and were split into two parts each. The geometries of the respective A/B parts were restrained to adopt similar geometries using SAME/SADI. The ADPs of corresponding atoms in parts A and B were constrained to adopt the same ADPs using EADP.

Table S1: Crystallographic details of **2**·CH_2Cl_2, **2**·C_7H_8, and [MeCN*t*Bu][$GaCl_4$].

Compound	**2** · 0.6 CH_2Cl_2	**2** · 3 C_7H_8	[MeCN*t*Bu][$GaCl_4$]
Chem. Formula	$C_{36}H_{58}Cl_2NP_3$ · 0.575 CH_2Cl_2	$C_{36}H_{58}Cl_2NP_3$ · 3 C_7H_8	[$C_6H_{12}N$][$GaCl_4$]
Formula weight [g/mol]	717.63	945.04	309.69
Color	colorless	colorless	colorless
Crystal system	triclinic	triclinic	monoclinic
Space group	$P\bar{1}$	$P\bar{1}$	$P2_1/m$
a [Å]	10.5403(6)	11.9493(5)	6.2886(2)
b [Å]	17.107(1)	13.7181(6)	7.2063(2)
c [Å]	34.625(2)	18.5992(9)	14.1421(4)
α [°]	76.292(2)	108.211(2)	90
β [°]	86.441(2)	94.127(2)	101.201(1)
γ [°]	78.729(2)	106.350(2)	90
V [Å^3]	5947.7(6)	2735.9(2)	628.68(3)
Z	6	2	2
$\rho_{calcd.}$ [g/cm^3]	1.202	1.147	1.636
μ [mm^{-1}]	0.388	0.242	2.993
T [K]	123(2)	123(2)	123(2)
Measured reflections	283684	13851	20450
Independent reflections	34681	13851	2309
Reflections with $I > 2\sigma(I)$	24879	10717	1798
R_{int}	0.0782	0.0580	0.0612
F(000)	2305	1020	308
$R_1(R[F^2>2\sigma(F^2)])$	0.0489	0.0523	0.0352
w$R_2(F^2)$	0.1008	0.1000	0.0476
GooF	1.036	1.049	1.097
No. of Parameters	1354	589	69
CCDC #	1939289	1939290	1939291

Table S2: Crystallographic details of [Mes*N(H)PCl_2*t*Bu][$GaCl_4$] · CH_2Cl_2, **12**, and **13** · PhF.

Compound	[Mes*N(H)PCl_2*t*Bu] [$GaCl_4$] · CH_2Cl_2	**12**	**13** · PhF
Chem. Formula	[$C_{22}H_{39}Cl_2NP$][$GaCl_4$] · CH_2Cl_2	$C_{48}H_{58}F_{10}NP_3$	$C_{48}H_{58}P_4F_{10}$ · C_6H_5F
Formula weight [g/mol]	715.86	931.86	1044.92
Color	colorless	yellow	yellow
Crystal system	monoclinic	triclinic	monoclinic
Space group	$P2_1/c$	$P\bar{1}$	$P2_1/n$
a [Å]	16.0225(8)	14.3400(8)	12.05338(4)
b [Å]	11.2599(5)	17.5014(9)	26.4839(8)
c [Å]	18.6925(9)	19.4609(9)	16.5203(5)
α [°]	90	84.018(2)	90
β [°]	97.382(2)	79.207(2)	98.021(2)
γ [°]	90	86.813(2)	90
V [Å^3]	3344.4(3)	4768.3(4)	5222.2(3)
Z	4	4	4
$\rho_{calcd.}$ [g/cm^3]	1.422	1.298	1.329
μ [mm^{-1}]	1.524	0.197	0.220
T [K]	123(2)	123(2)	173(2)
Measured reflections	106721	274725	75106
Independent reflections	9803	27808	15204
Reflections with $I > 2\sigma(I)$	6427	20044	7912
R_{int}	0.1175	0.0942	0.0976
F(000)	1472	1952	2184
$R_1(R[F^2>2\sigma(F^2)])$	0.0446	0.0502	0.0552
w$R_2(F^2)$	0.1147	0.1158	0.1259
GooF	1.045	1.059	1.018
No. of Parameters	355	1187	697
CCDC #	1939292	1939293	1939294

Table S3: Crystallographic details of **16**.

Compound	**16**
Chem. Formula	$C_{72}H_{116}N_2P_6$
Formula weight [g/mol]	1195.48
Color	orange
Crystal system	triclinic
Space group	$P\bar{1}$
a [Å]	10.2980(6)
b [Å]	11.4972(8)
c [Å]	30.013(2)
α [°]	90.254(2)
β [°]	94.051(2)
γ [°]	94.775(2)
V [Å^3]	3532.1(4)
Z	2
$\rho_{calcd.}$ [g/cm^3]	1.124
μ [mm^{-1}]	0.192
T [K]	123(2)
Measured reflections	26372
Independent reflections	26372
Reflections with $I > 2\sigma(I)$	19237
R_{int}	0.1140
$F(000)$	1304
$R_1(R[F^2>2\sigma(F^2)])$	0.0739
$wR_2(F^2)$	0.1381
GooF	1.040
No. of Parameters	824
CCDC #	1939295

3 Syntheses of Starting Materials

3.1 Mes*N(H)PPMes*

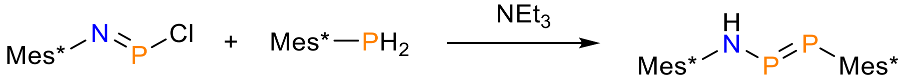

Mes*N(H)PPMes* is prepared according to a modified literature procedure.[10] A colorless solution of Mes*PH_2 (6.67 g, 23.95 mmol) in Et_2O (70 mL) is added to a stirred, red solution of Mes*NPCl (8.12 g, 24.92 mmol) in Et_2O at −80 °C, resulting in an orange mixture. After adding NEt_3 (6.7 mL, 48.33 mmol) at the same temperature, the mixture is warmed to ambient temperature and stirred for 2 hours. The volatiles are subsequently removed *in vacuo* and the yellow crystals are thoroughly dried. Afterwards, the solids are placed in a thimble of a previously evacuated Soxhlet extractor and the product is extracted with *n*-pentane (250 mL) over the course of 25 h. The solvent is again removed *in vacuo* and the residue is recrystallized from hot benzene. Storage at ambient temperature overnight yields yellow needle-shaped crystals. Yield: 10.26 g (18.1 mmol, 75 %).

Mp. 224 °C. CHN calcd. (found) in %: C 76.15 (76.65), H 10.47 (8.55), N 2.47 (2.57). $^{31}P\{^1H\}$ NMR (CD_2Cl_2, 121.5 MHz): δ = 317.1 (d, $^1J(^{31}P,^{31}P)$ = −531 Hz, 1 P, *P*Mes*), 448.5 (d, $^1J(^{31}P,^{31}P)$ = −531 Hz, 1 P, Mes*N(H)*P*). ^1H NMR (CD_2Cl_2, 300.1 MHz): δ = 1.31 (s, 9 H, *p*-*t*Bu), 1.35 (s, 9 H, *p*-*t*Bu), 1.49 (s, 18 H, *o*-*t*Bu), 1.59 (s, 18 H, *o*-*t*Bu), 6.50 (d, $^2J(^1H,^{31}P)$ = 6.8 Hz, 1 H, N*H*), 7.40 (s, 2 H, *m*-H), 7.45 (s, 2 H, *m*-H). $^{13}C\{^1H\}$ NMR (CD_2Cl_2, 75.5 MHz): δ = 31.7 (s, *p*-C(CH_3)$_3$), 31.7 (s, *p*-C(CH_3)$_3$), 33.4 (d, $^5J(^{13}C,^{31}P)$ = 2.8 Hz, *o*-C(CH_3)$_3$, Mes*N), 34.9 (dd, $^4J(^{13}C,^{31}P)$ = 6.6 Hz, $^5J(^{13}C,^{31}P)$ = 2.1 Hz, *o*-C(CH_3)$_3$, PMes*), 35.3 (s, *p*-*C*(CH_3)$_3$), 35.4 (s, *p*-*C*(CH_3)$_3$), 37.4 (s, *o*-*C*(CH_3)$_3$, Mes*N), 39.2 (s, *o*-*C*(CH_3)$_3$, PMes*), 122.5 (s, *m*-C, PMes*), 123.6 (s, *m*-C, Mes*N), 128.9 (s, *ipso*-C, PMes*), 148.8 (s, *p*-C, Mes*N), 149.7 (s, *p*-C, PMes*), 157.2 (s, *o*-C), 157.3 (s,

o-C), *ipso*-C (Mes*N) not observed. Raman (633 nm, 20 s, 20 scans, cm^{-1}): $\tilde{\nu}$ = 3331 (1), 2963 (1), 2903 (1), 2775 (1), 2705 (1), 1590 (1), 1445 (1), 1284 (1), 1243 (1), 1225 (1), 1199 (1), 1143 (1), 1127 (1), 1037 (1), 1024 (1), 923 (1), 882 (1), 822 (1), 804 (1), 751 (1), 689 (1), 608 (10), 567 (1), 525 (1), 255 (1), 135 (1), 121 (1), 99 (1).

Figure S1: NMR and Raman spectra of Mes*N(H)PPMes* (solvent signals marked by asterisks).

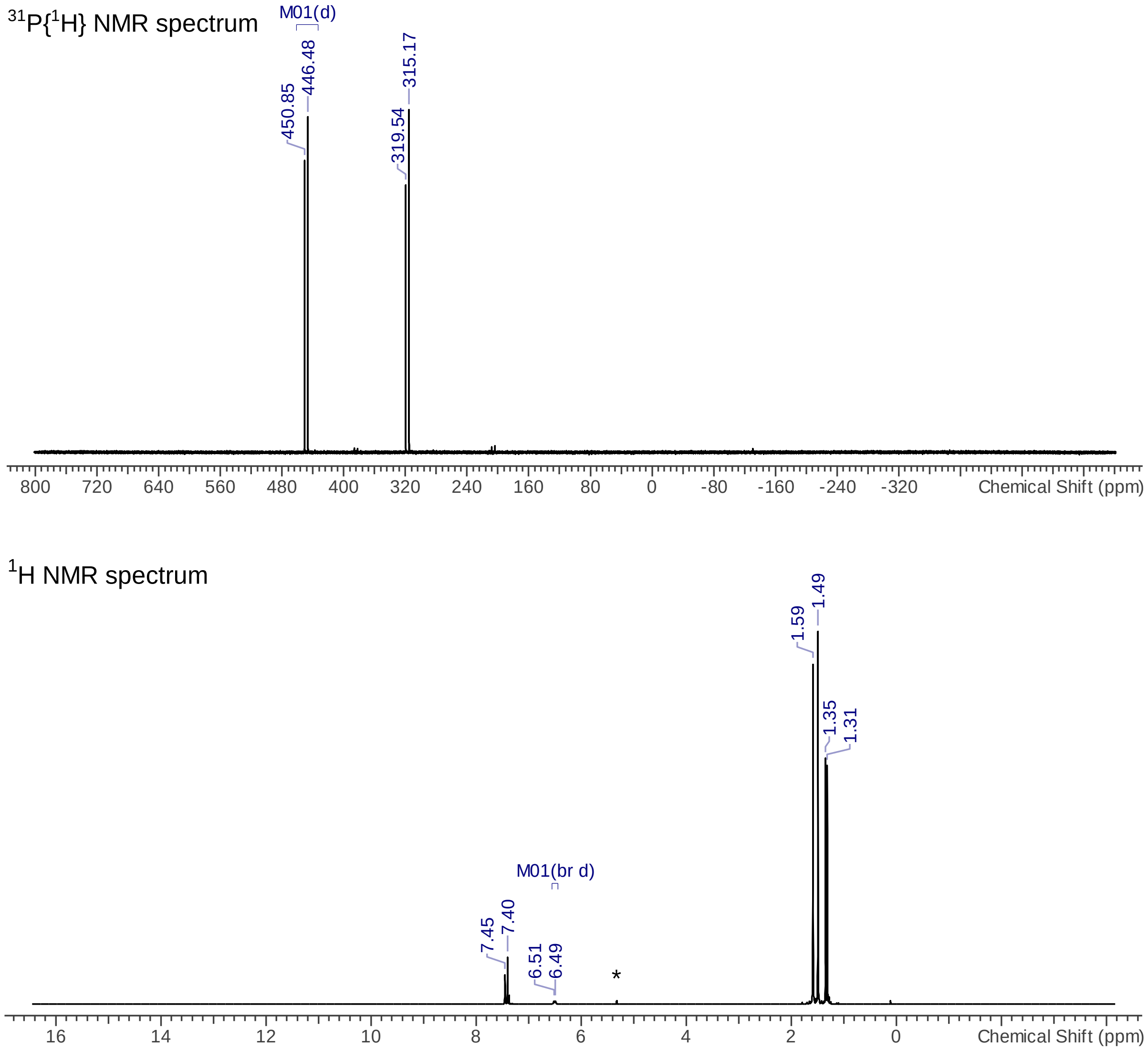

Figure S1 continued.

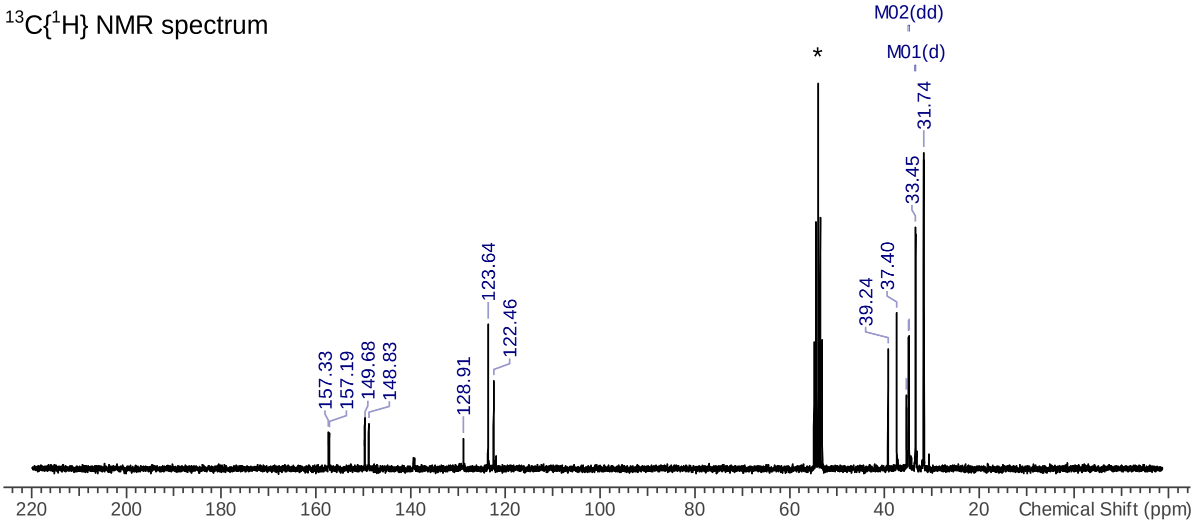

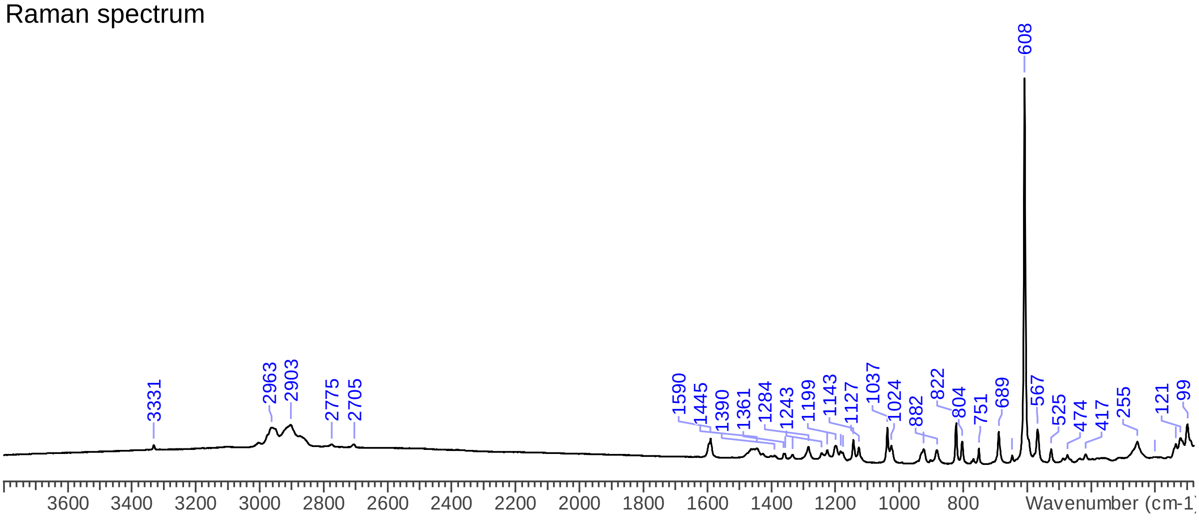

3.2 [Mes*N(μ-PCl)$_2$PMes*] (2)

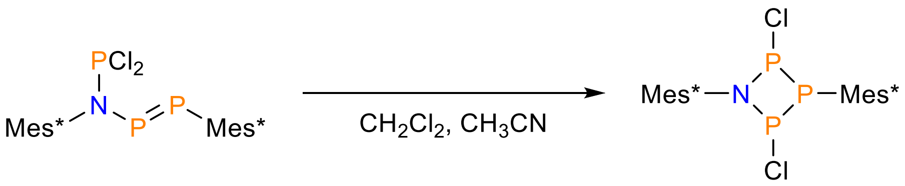

Mes*N(PCl$_2$)PPMes* (1.39 g, 2.07 mmol) is dissolved in CH_2Cl_2 (8 mL) and CH_3CN (16 mL) is added afterwards, resulting in precipitation of the starting material, which slowly dissolves again as the reaction progresses. After storage at ambient temperature overnight, colourless crystals of **2** · 0.6 CH_2Cl_2 are obtained. The

supernatant is removed by syringe and the crystals are dried *in vacuo*, resulting in the removal of approx. 0.4 eq. of CH_2Cl_2. Yield: 1.00 g (1.46 mmol, 70 %).

Mp. 154 °C (decomp. starting at 140 °C). CHN calcd. (found) in %: C 63.41 (63.69), H 8.59 (8.46), N 2.04 (2.04). $^{31}P\{^{1}H\}$ NMR (CD_2Cl_2, 121.5 MHz): δ = −27.7 (t, $^{1}J(^{31}P,^{31}P)$ = −172 Hz, 1 P, *P*Mes*), 206.5 (d, $^{1}J(^{31}P,^{31}P)$ = −172 Hz, 2 P, µ-*P*Cl). ^{1}H NMR (C_6D_6, 300.1 MHz): δ = 1.18 (s, 9 H, *t*Bu), 1.22 (s, 9 H, *t*Bu), 1.29 (s, 9 H, *t*Bu), 1.51 (s, 18 H, *t*Bu), 1.73 (s, 18 H, *t*Bu), 4,28 (s, CH_2Cl_2) 7.36 (d, $^{4}J(^{1}H,^{31}P)$ = 2.6 Hz, 2 H, *m*-H, PMes*), 7.41 (d, *J* = 2.5 Hz, 1 H, *m*-H) , 7.50 (d, *J* = 2.3 Hz, 1 H, *m*-H). $^{13}C\{^{1}H\}$ NMR (CD_2Cl_2, 75.5 MHz): δ = 31.4 (s, $C(CH_3)_3$), 31.6 (s, $C(CH_3)_3$), 34.0 (m, $C(CH_3)_3$), 35.0 (s, $C(CH_3)_3$), 35.1 (s, $C(CH_3)_3$), 35.7 (s, $C(CH_3)_3$), 37.8 (s, $C(CH_3)_3$), 38.9 (s, $C(CH_3)_3$), 39.7 (s, $C(CH_3)_3$), 39.8 (s, $C(CH_3)_3$), 122.8 (s, *m*-C), 125.3 (s, *m*-C), 128.1 (s, *m*-C), 148.7 (s, *o/p*-C), 149.4 (s, *o/p*-C), 149.8 (s, *o/p*-C). IR (ATR, 32 scans, cm^{-1}): $\tilde{\nu}$ = 2958 (s), 2902 (m), 2866 (m), 1597 (w), 1583 (w), 1518 (w), 1468 (m), 1444 (m), 1439 (m), 1412 (m), 1392 (m), 1362 (s), 1263 (m), 1240 (m), 1211 (m), 1201 (m), 1171 (m), 1126 (w), 1099 (s), 1026 (w), 935 (w), 918 (w), 887 (s), 879 (s), 864 (vs), 820 (w), 752 (m), 741 (s), 706 (m), 654 (w), 646 (m), 607 (w), 580 (m), 565 (w), 542 (w). Raman (633 nm, 10 s, 20 scans, cm^{-1}): $\tilde{\nu}$ = 3050 (1), 2963 (7), 2926 (6), 2905 (8), 2874 (3), 2780 (1), 2711 (1), 2113 (1), 1599 (4), 1582 (4), 1478 (2), 1464 (3), 1443 (3), 1413 (1), 1393 (1), 1362 (1), 1292 (2), 1282 (2), 1243 (1), 1203 (2), 1171 (5), 1139 (5), 1127 (3), 1099 (4), 1026 (3), 1017 (2), 936 (2), 925 (2), 893 (1), 870 (1), 820 (6), 767 (1), 750 (1), 705 (2), 646 (1), 586 (1), 567 (8), 543 (2), 517 (1), 498 (3), 474 (6), 454 (6), 429 (2), 412 (5), 395 (2), 375 (3), 353 (3), 284 (2), 261 (5), 201 (3), 179 (8), 166 (10), 142 (8).

Single crystals of **2** · 0.6 CH_2Cl_2 suitable for SC-XRD could be grown from the supernatant as described above.

Figure S2: NMR, IR and Raman spectra of [Mes*N(μ-PCl)$_2$PMes*] (solvent signals marked by asterisks).

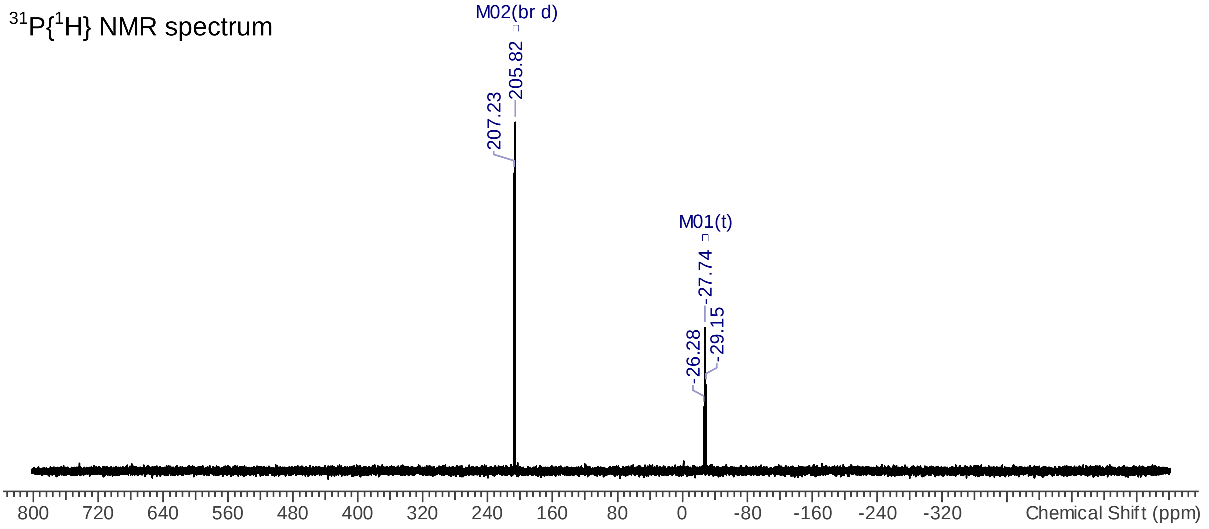

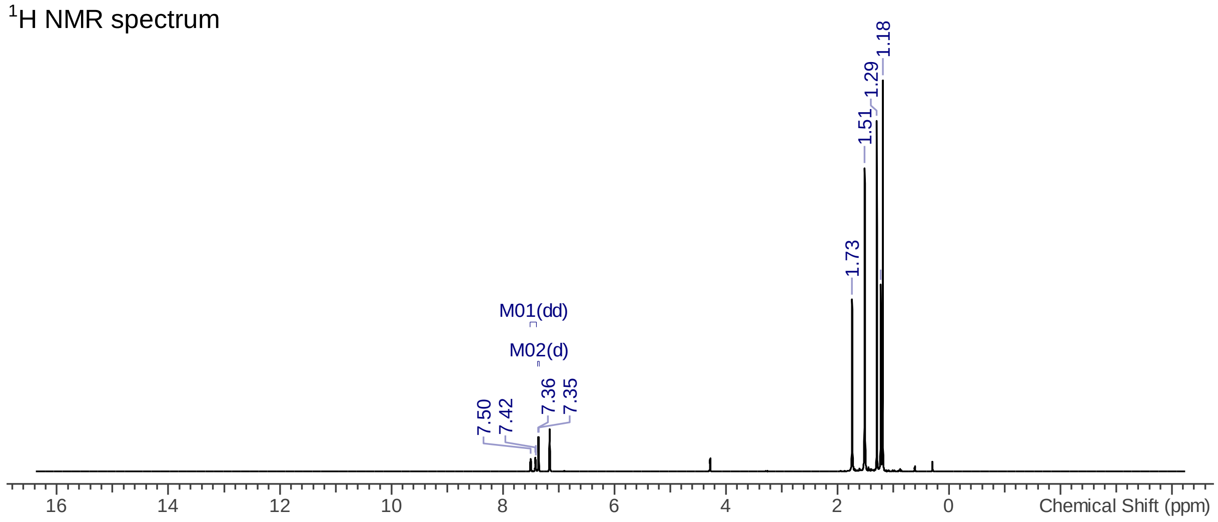

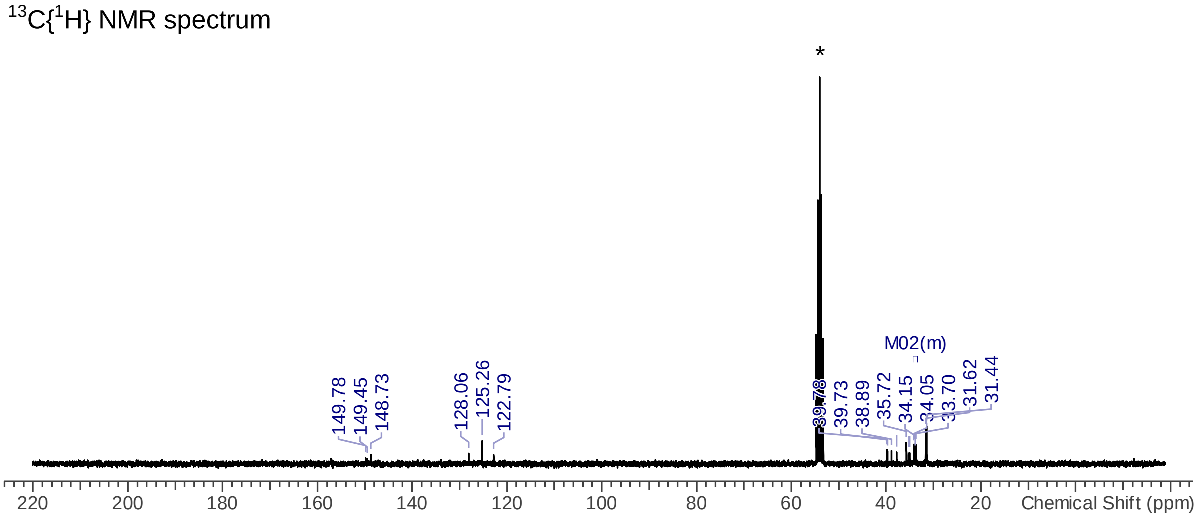

Figure S2 continued.

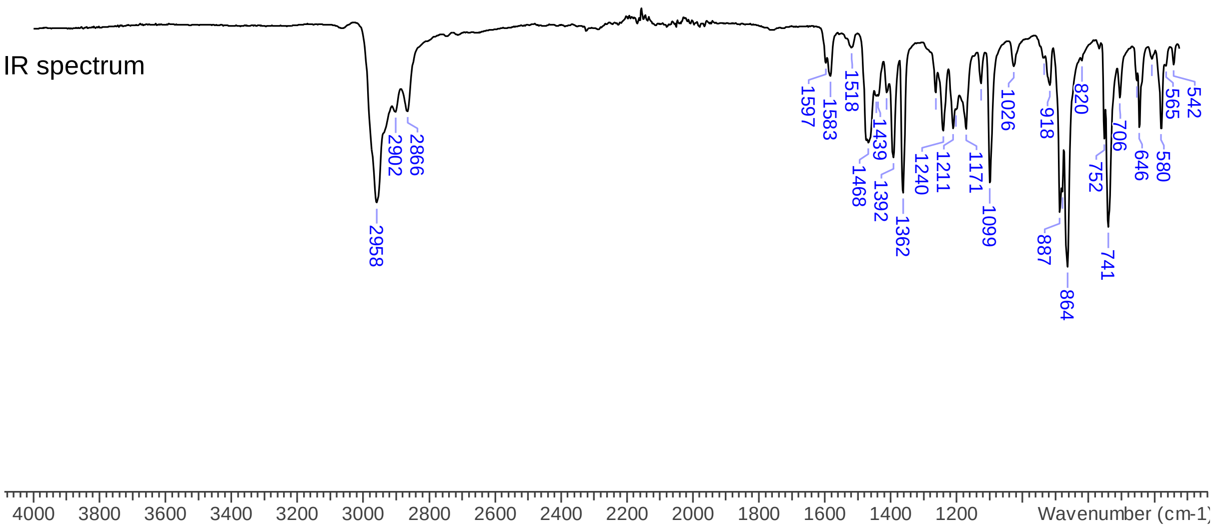

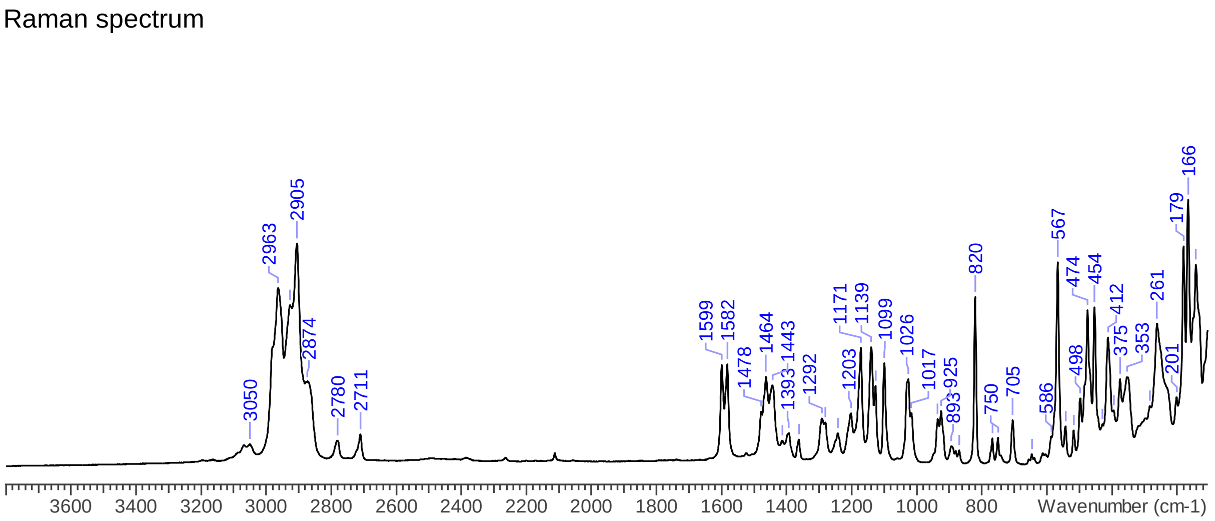

Figure S3: In situ ^{31}P NMR spectra of the isomerization reaction, showing selective formation of the desired ring system.

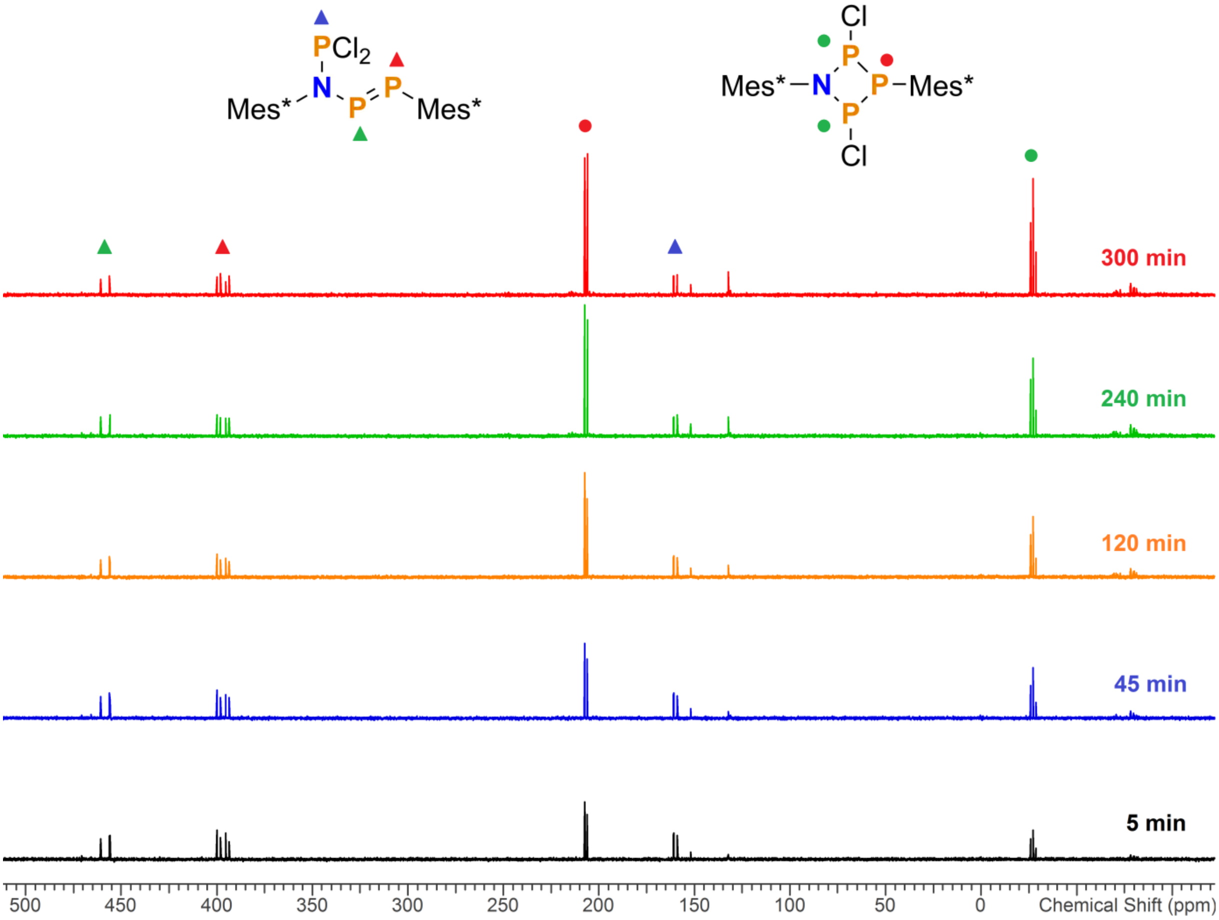

4 Syntheses of Compounds

4.1 Mes*P_6Mes* (5)

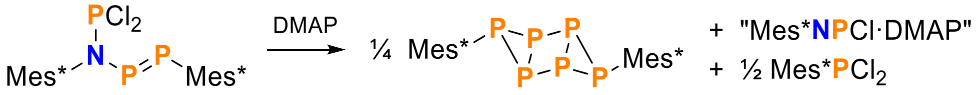

Route 1: Mes*N(PCl_2)PPMes* (0.30 g, 0.45 mmol) and 4-dimethylaminopyridine (0.06 g, 0.47 mmol) are combined and dissolved in THF (5 mL). After storage at ambient temperature for 4 days, the solvent is evaporated *in vacuo* and the residue dissolved in CH_2Cl_2. Concentrating the solution and subsequent crystallization at ambient temperature yields Mes*P_6Mes* as colorless solid. Yield: 40 mg (0.06 mmol, 80 %).

Mp. 212 °C (dec.). CHN calcd. (found) in %: C 63.90 (63.54), H 8.64 (8.55). $^{31}P\{^1H\}$ NMR (CD_2Cl_2, 121.5 MHz): δ = −107.8 (m, 2 P), −96.1 (m, 4 P). ^1H NMR (CD_2Cl_2, 300.1 MHz): δ = 1.16 (s, 9 H, *p*-*t*-Bu), 1.64 (s, 18 H, *o*-*t*-Bu), 7.02 (s, 2 H, *m*-H). Raman (633 nm, 20 s, 20 scans, cm^{-1}): $\tilde{\nu}$ = 2978 (1), 2970 (2), 2960 (2), 2928 (2), 2902 (3), 2865 (1), 2778 (1), 2709 (1), 1590 (2), 1529 (1), 1476 (1), 1463 (1), 1441 (2), 1400 (1), 1392 (1), 1364 (1), 1359 (1), 1283 (1), 1243 (1), 1203 (1), 1185 (1), 1173 (1), 1152 (1), 1129 (2), 1032 (2), 1026 (2), 1018 (2), 933 (1), 917 (1), 894 (1), 820 (5), 774 (1), 746 (1), 588 (1), 579 (1), 566 (7), 538 (10), 485 (1), 477 (1), 444 (3), 434 (1), 417 (7), 395 (2), 383 (1), 362 (1), 316 (1), 263 (2), 252 (2), 203 (1), 129 (3), 119 (2).

The identity of the compound was additionally verified by determination of the unit cell.

Figure S4: NMR and Raman spectra of Mes*P_6Mes* (solvent signals indicated by asterisk).

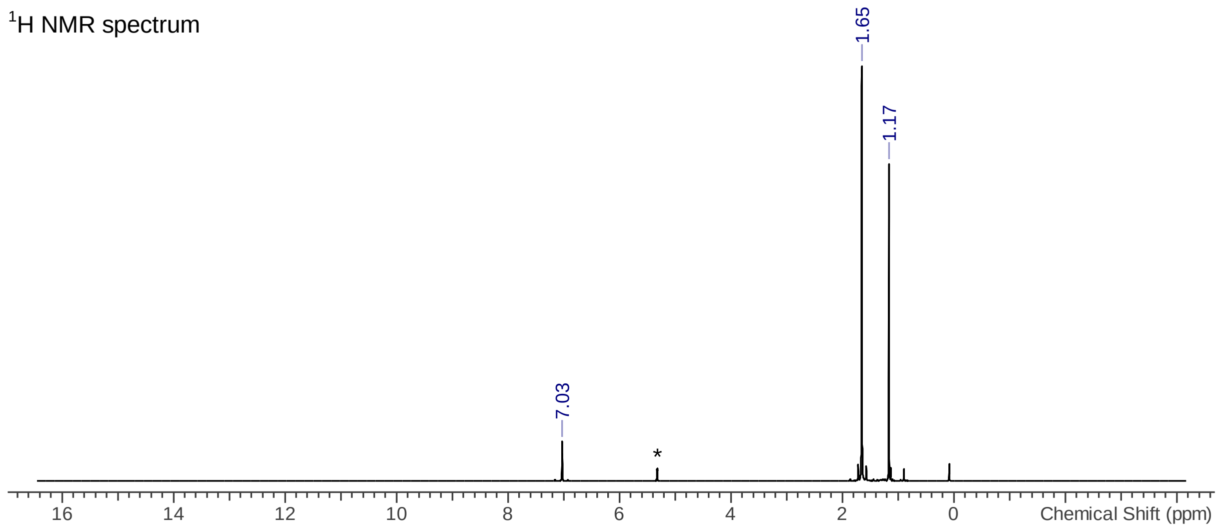

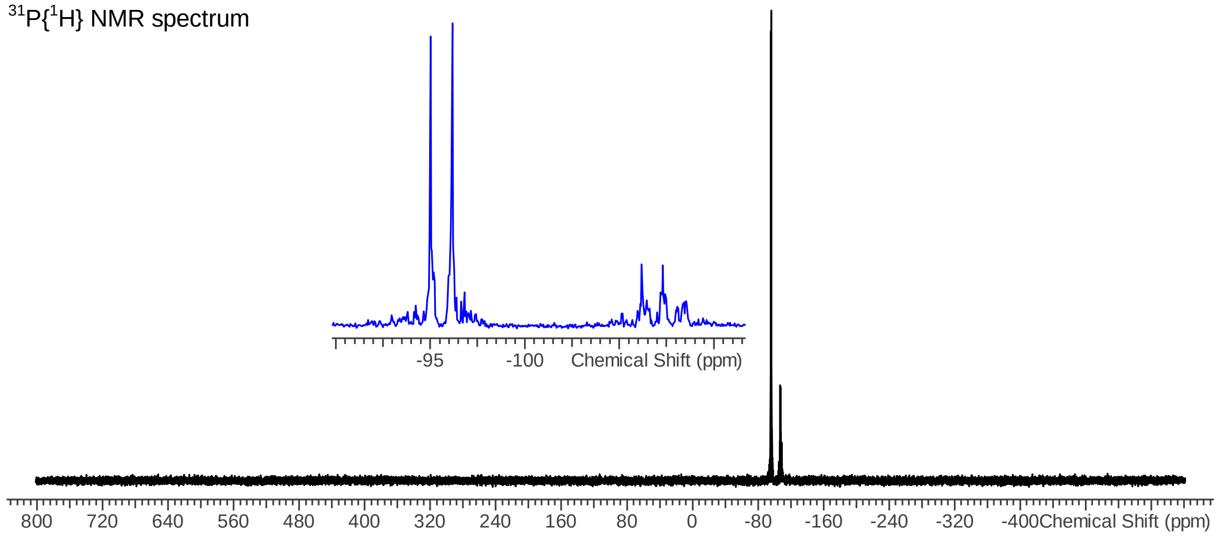

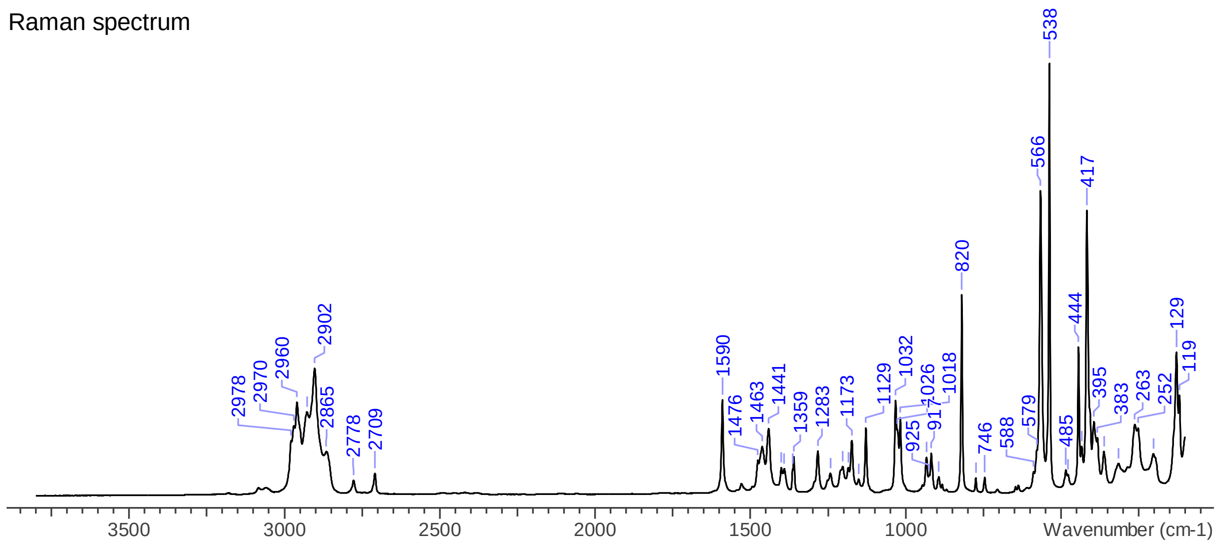

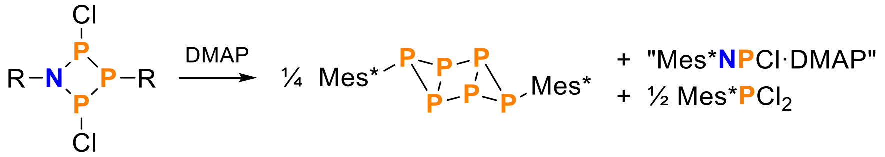

Route 2: [Mes*N(µ-PCl)$_2$PMes*] (0.03 g, 0.05 mmol) and 4-dimethylaminopyridine (0.01 g, 0.09 mmol) are placed in an NMR tube and dissolved in CD_2Cl_2 (0.5 mL). Over the course of the next 4 days, the solution changes from colourless to orange.

$^{31}P\{^1H\}$ NMR (CD_2Cl_2, 121.5 MHz): δ = −107.7 (m, Mes**P*$_6$Mes*), −96.1 (m, Mes**P*$_6$Mes*), 109.7 (s, Mes*N*P*(Cl)DMAP), 153.4 (s, Mes**P*Cl$_2$).

Figure S5: NMR spectrum of the reaction of [Mes*N(µ-PCl)$_2$PMes*] with DMAP.

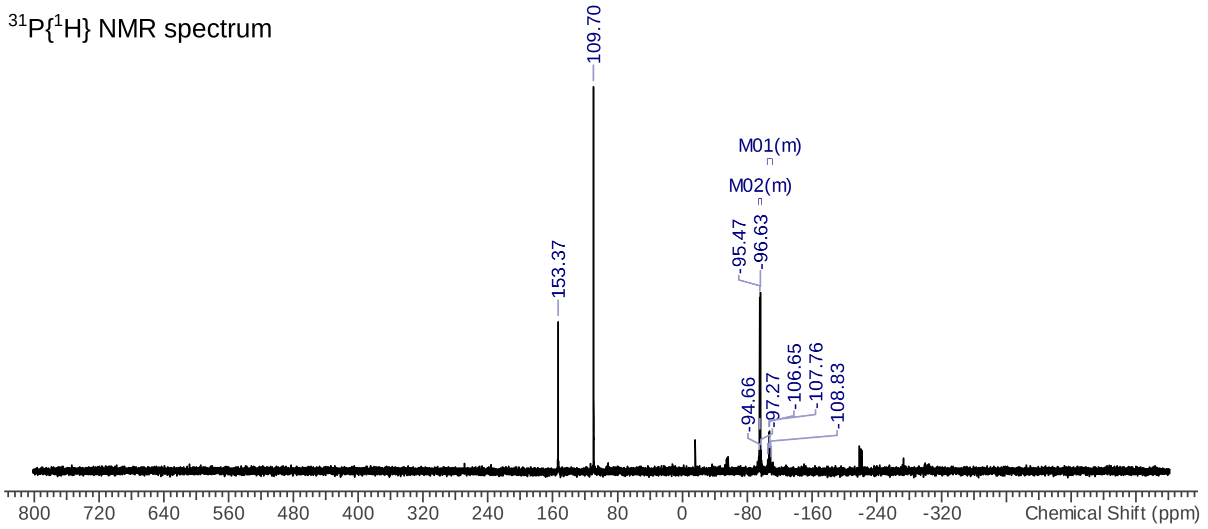

Figure S6: Time-resolved $^{31}P\{^{1}H\}$ NMR spectra of the reaction of [Mes*N(μ-PCl)$_2$PMes*] with DMAP.

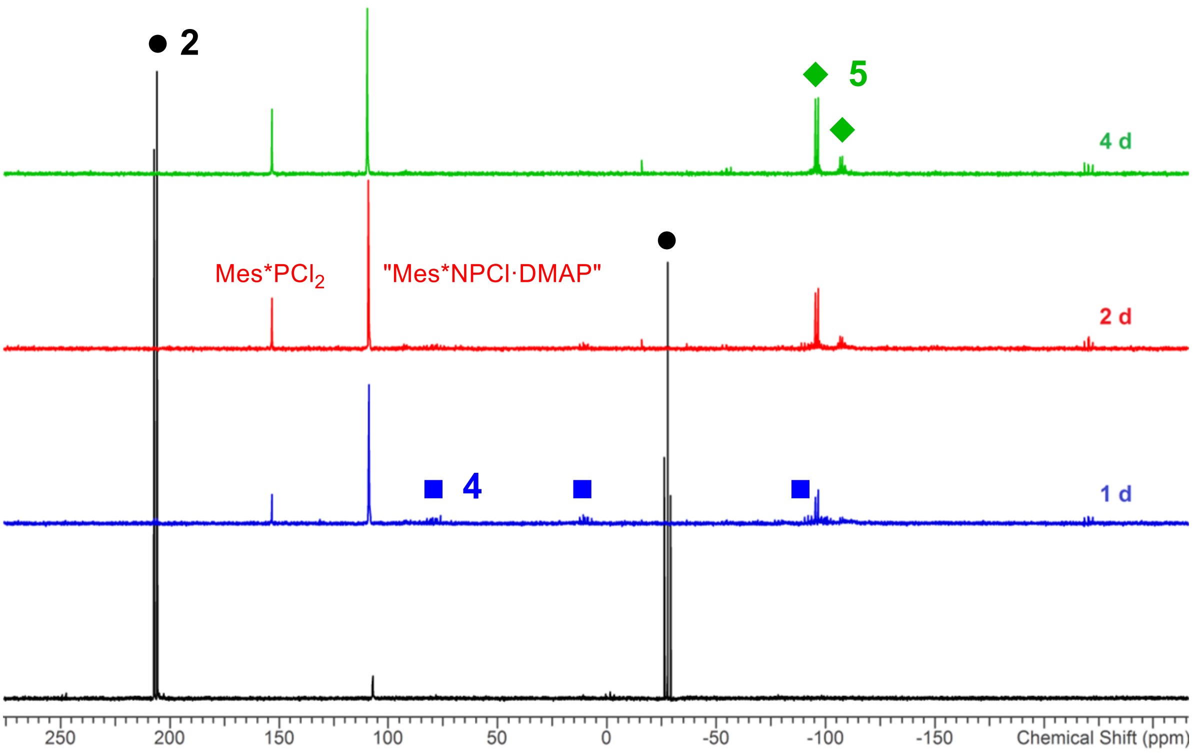

4.2 Mes*NPCl + DMAP

To understand the origin of the signal at 109.7 ppm in the ^{31}P NMR spectrum in Figure S5, Mes*NPCl (which is formally obtained by cycloreversion of the NP_3 ring system **2**) was treated with DMAP:

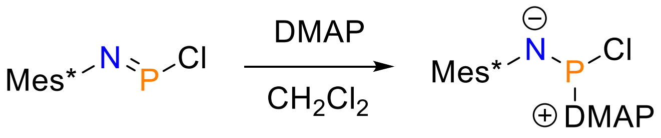

After dissolving Mes*NPCl (0.42 g, 1.30 mmol) and 4-dimethylaminopyridine (0.16 g, 1.29 mmol) in CH_2Cl_2 (4 mL) and letting it react for 5 minutes, the solution is concentrated and eventually stored in the refrigerator overnight. The next day, the supernatant is removed by syringe and solids are dried *in vacuo*. Yield: 0.34 g (0.76 mmol, 59 %).

Mp. 113 °C. CHN calcd. (found) in %: C 67.02 (66.50), H 8.77 (7.66), N 9.38 (9.32). ^{31}P NMR (CD_2Cl_2, 121.5 MHz): δ = 121.0 (s). ^{1}H NMR (CD_2Cl_2, 300.1 MHz): δ = 1.33 (s, 9 H, *p*-*t*Bu), 1.46 (s, 18 H, *o*-*t*Bu), 3.03 (s, 6 H, N(CH_3)$_2$), 6.54 (m, 2 H, *m*-H, DMAP), 7.36 (d, $^{5}J(^{1}H,^{31}P)$ = 2 Hz, 2 H, *m*-H, Mes*), 8.20 (m, 2 H, *o*-H, DMAP). ^{13}C{^{1}H} NMR (CD_2Cl_2, 75.5 MHz): δ = 31.1 (d, $^{5}J(^{13}C,^{31}P)$ = 2.2 Hz, *o*-C(CH_3)$_3$), 31.8 (s, *p*-C(CH_3)$_3$), 35.4 (s, *p*-*C*(CH_3)$_3$), 36.3 (d, $^{4}J(^{13}C,^{31}P)$ = 1.7 Hz, *o*-*C*(CH_3)$_3$), 39.7 (s, N(CH_3)$_2$), 107.1 (s, *m*-C, DMAP), 122.5 (d, $^{4}J(^{13}C,^{31}P)$ = 2.8 Hz, *m*-C, Mes*), 138.1 (s, Mes*), 138.6 (s, Mes*), 140.5 (d, $^{3}J(^{13}C,^{31}P)$ = 12.1 Hz, *o*-C, Mes*), 147.0 (s, *o*-C, DMAP), 155.8 (s, *p*-C, DMAP). IR (ATR, 32 scans, cm^{-1}): $\tilde{\nu}$ = 2958 (m), 2949 (m), 2906 (m), 2870 (m), 1645 (s), 1626 (s), 1599 (m), 1560 (s), 1529 (m), 1491 (s), 1454 (s), 1435 (s), 1392 (s), 1358 (s), 1336 (m), 1313 (m), 1267 (m), 1255 (m), 1244 (m), 1215 (vs), 1132 (m), 1063 (m), 1049 (s), 1001 (vs), 943 (m), 924 (s), 889 (s), 881 (s), 823 (s), 810 (vs), 769 (m), 758 (s), 744 (m), 646 (m), 636 (m), 552 (m). Raman (633 nm, 20 s, 20 scans, cm^{-1}): $\tilde{\nu}$ = 3093 (2), 3079 (2), 2981 (2), 2965 (3), 2926 (3), 2909 (3), 2826 (2), 2774 (1), 2707 (1), 1629 (2), 1560 (2), 1524 (1), 1509 (2), 1466 (2), 1444 (2), 1427 (2), 1316 (2), 1290 (1), 1217 (2), 1199 (1), 1047 (7), 1018 (2), 1004 (1), 944 (3), 924 (1), 821 (4), 763 (5), 705 (1), 657 (1), 565 (2), 436 (1), 391 (1), 249 (1), 190 (2), 126 (4).

According to the analytical data, a weak 1:1 adduct between Mes*NPCl and DMAP is formed. The ^{31}P NMR signal is somewhat broadened, indicating dynamic exchange. However, the resonance does not correspond exactly to the signal observed in Figure S5. While this might be attributed to concentration effects, it hampers an unequivocal assignment of the signal in question.

Attempts to obtain single crystals suitable for SC-XRD remained futile.

Figure S7: NMR, IR and Raman spectra of the reaction of Mes*NPCl with DMAP (solvent signals marked by asterisks).

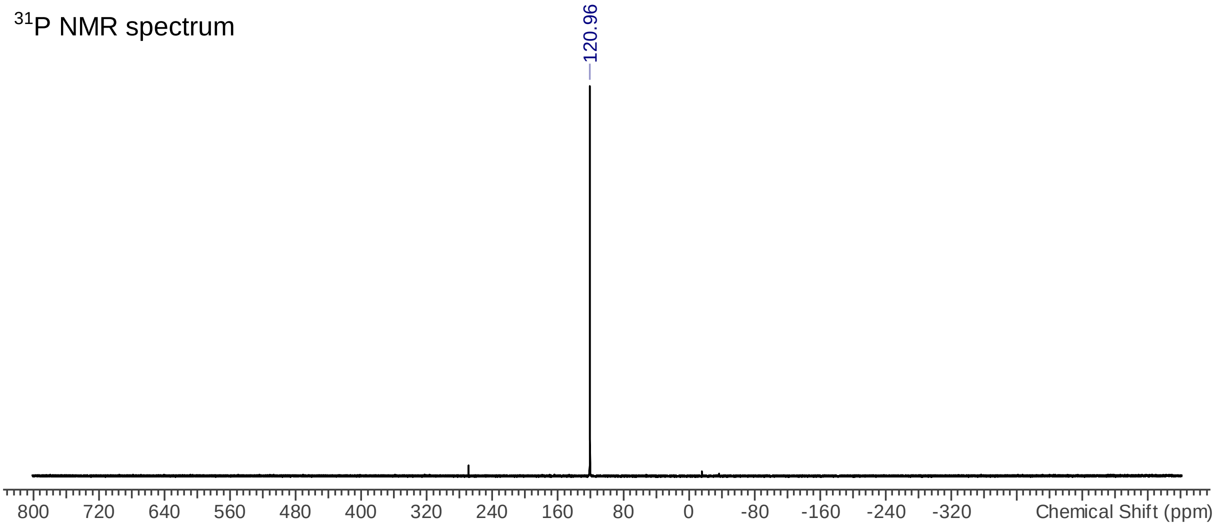

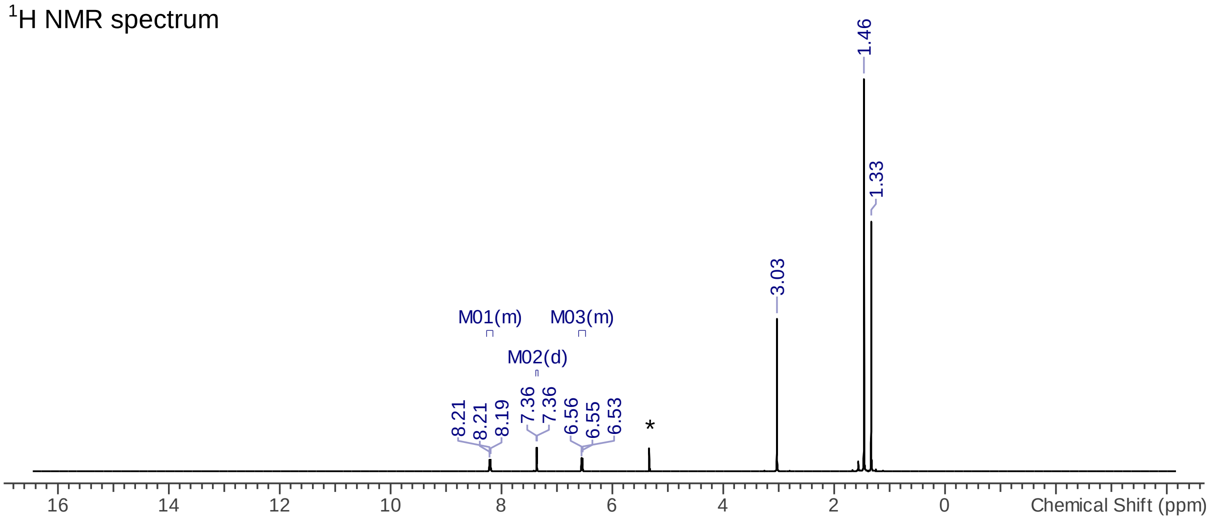

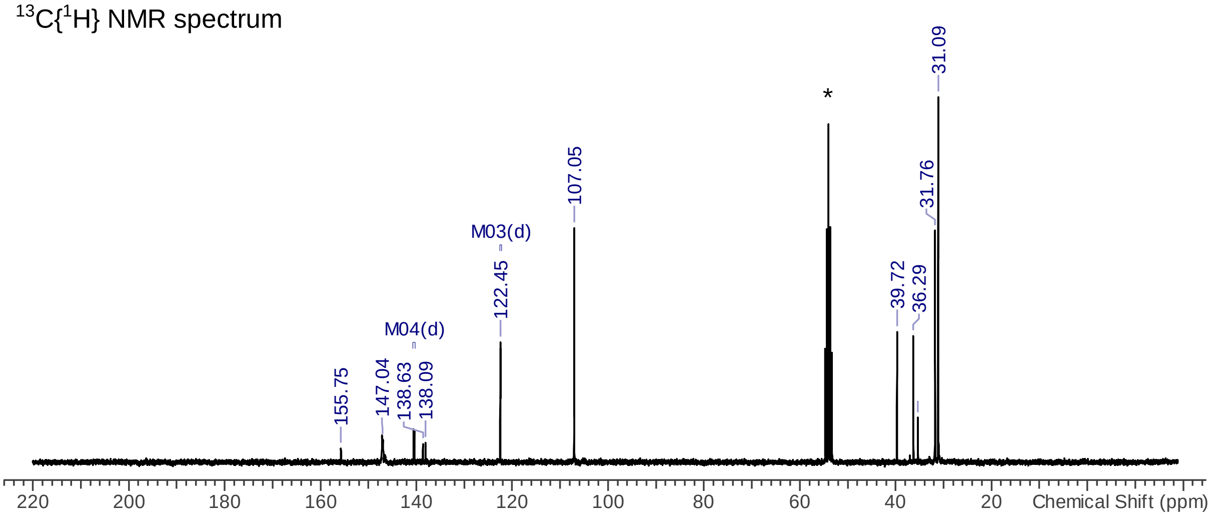

Figure S7 continued.

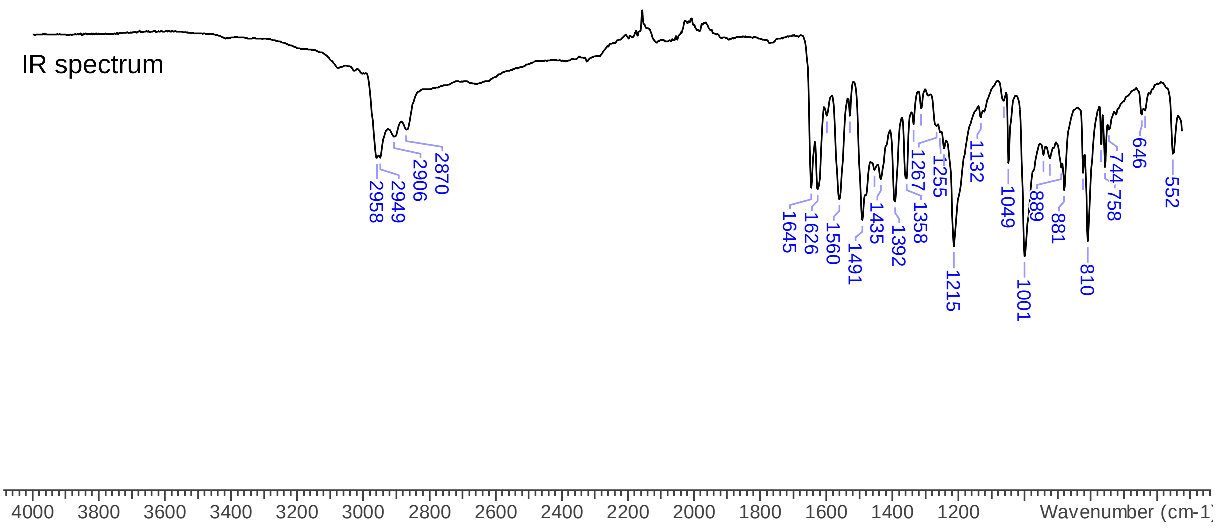

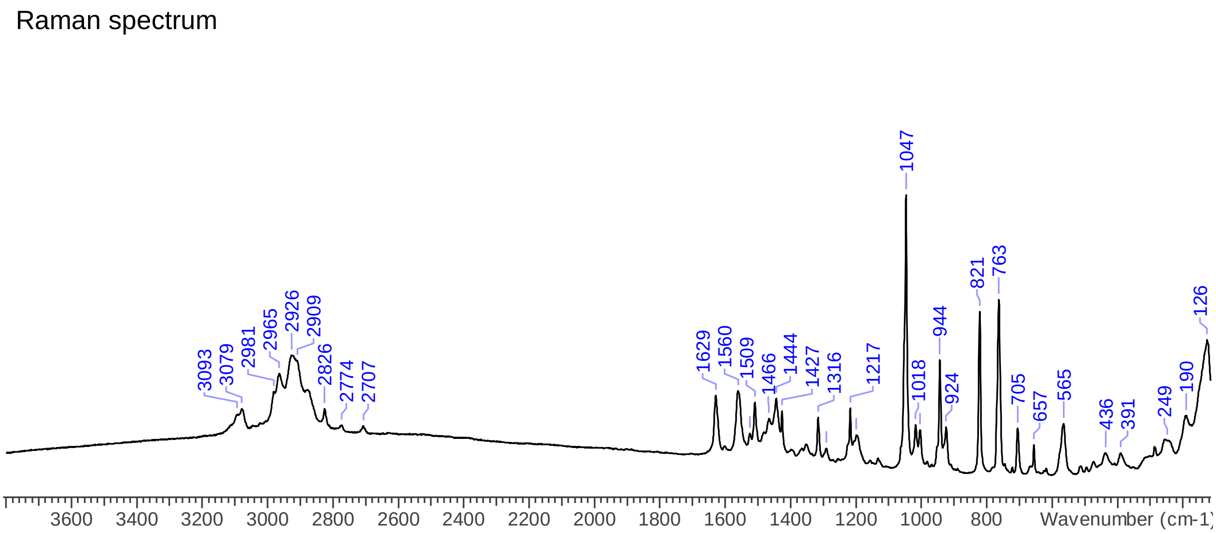

4.3 [Mes*$_2$NP$_3$Cl][GaCl$_4$] (6)

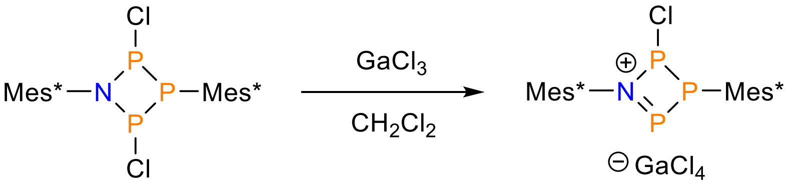

A solution of $GaCl_3$ (0.07 g, 0.39 mmol) in CH_2Cl_2 (1 mL) is added to a stirred, colorless solution of [Mes*N(μ-PCl)$_2$PMes*] (0.25 g, 0.37 mmol) in CH_2Cl_2 (2 mL) at −80 °C, resulting in an instant color change to red. The mixture is subsequently stirred for

another 1.5 h at a maximum temperature of −50 °C, concentrated and eventually stored at −80 °C.

The product could not be isolated and the reaction mixture was used for NMR measurements.

$^{31}P\{^1H\}$ NMR (CD_2Cl_2, 121.5 MHz, 193 K): δ = 114.2 (dd, $^1J(^{31}P,^{31}P)$ = −317 Hz, $^2J(^{31}P,^{31}P)$ = 107 Hz, 1 P, *P*Cl), 175.4 (dd, $^1J(^{31}P,^{31}P)$ = −470 Hz, $^1J(^{31}P,^{31}P)$ = −317 Hz, 1 P, *P*Mes*), 446.4 (dd, $^1J(^{31}P,^{31}P)$ = −470 Hz, $^2J(^{31}P,^{31}P)$ = 107 Hz, 1 P, P^+).

Figure S8: Variable temperature $^{31}P\{^1H\}$ NMR spectra of the reaction of [Mes*N(μ-PCl)$_2$PMes*] with $GaCl_3$.

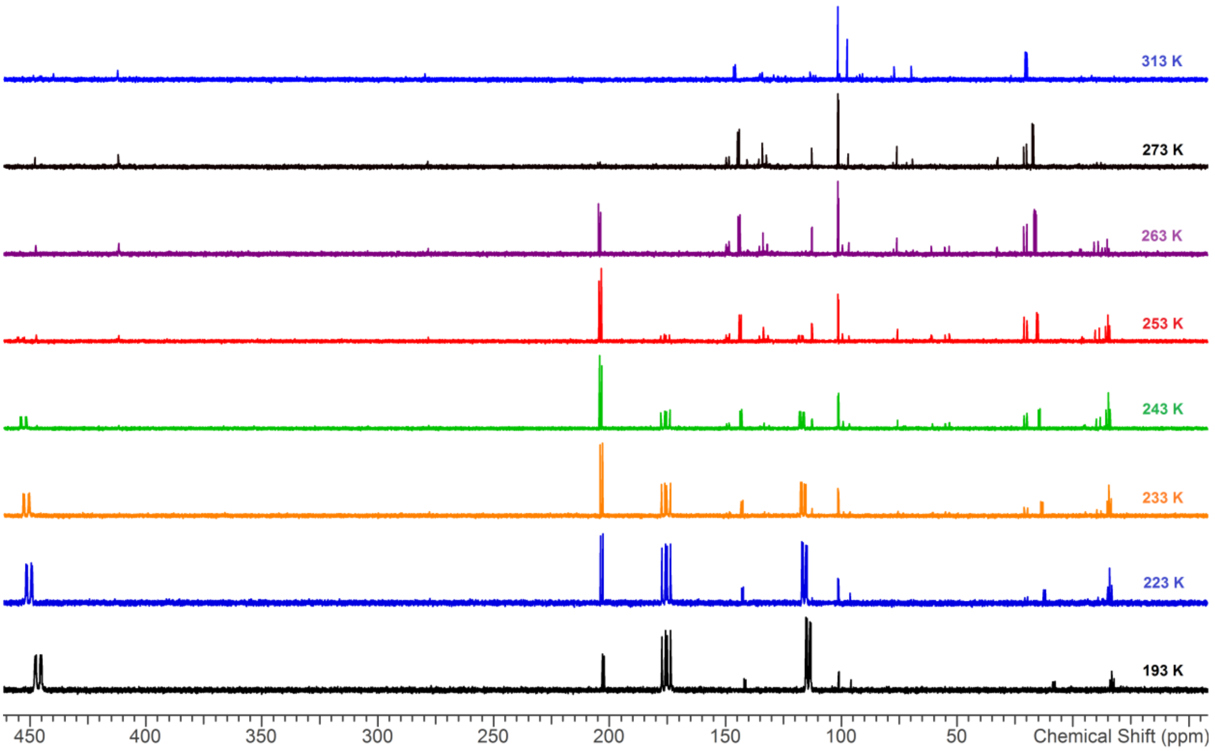

Attempts to crystallize the azatriphosphenium salt **6** from the reaction mixture at temperatures below−40 °C reproducibly led to the formation of a few crystals of [MeCN*t*Bu][$GaCl_4$]. Moreover, a few crystals of a second decomposition product ([Mes*N(H)PCl$_2$*t*Bu][$GaCl_4$], $\delta(^{31}P)$ = 97.1 ppm) could be obtained under the same conditions (*cf.* Structure Elucidation, p. S4).

4.4 [Mes*N(μ-P(C_6F_5))$_2$PMes*] (12)

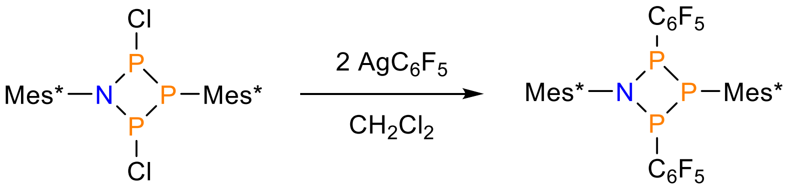

[Mes*N(μ-PCl)$_2$PMes*] (0.30 g, 0.46 mmol) and AgC_6F_5 (0.29 g, 0.91 mmol) are dissolved in CH_2Cl_2 (5 mL) at −80 °C. Afterwards, the flask is wrapped with aluminium foil and the mixture is slowly warmed to ambient temperature and stirred for 24 h. The solvent is then removed *in vacuo*, the residue is dissolved in CH_2Cl_2 again and the insoluble AgCl is filtered off with a G4 frit. The yellow filtrate is concentrated and CH_3CN is added until the solution becomes slightly cloudy. Storage at room temperature overnight yields yellow block-shaped crystals. The supernatant is removed by syringe and the solids are dried *in vacuo*. Yield: 0.20 g (0.21 mmol, 46 %).

Mp. 80 °C (dec.). CHN calcd. (found) in %: C 61.87 (61.16), H 6.27 (5.94), N 1.50 (1.62). ^{31}P{^{1}H} NMR (CD_2Cl_2, 121.5 MHz): δ = −66.8 (m, 1 P, *P*Mes*), 119.6 (dd, ^{1}J(^{31}P,^{31}P) = −133 Hz, 2 P, μ-*P*(C_6F_5)). ^{1}H NMR (CD_2Cl_2, 300.1 MHz): δ = 0.77 (s, 9 H, *t*Bu), 1.21 (s, 9 H, *t*Bu), 1.24 (s, 9 H, *t*Bu), 1.31 (s, 18 H, *o*-*t*Bu), 1.41 (s, 9 H, *t*Bu), 6.96 (d, ^{4}J(^{1}H,^{31}P) = 3 Hz, 1 H, *m*-H, PMes*), 7.36 (s, 1 H, *m*-H), 7.36 (s, 1 H, *m*-H), 7.37 (s, 1 H, *m*-H). ^{13}C{^{1}H} NMR (CD_2Cl_2, 75.5 MHz): δ = 31.3 (s, C(CH_3)$_3$), 31.7 (s, C(CH_3)$_3$), 33.6 (s, C(CH_3)$_3$), 33.9 (m, C(CH_3)$_3$), 35.0 (s, *C*(CH_3)$_3$), 36.8 (s, *C*(CH_3)$_3$), 39.3 (s, *C*(CH_3)$_3$), 40.0 (s, *C*(CH_3)$_3$), 40.0 (s, *C*(CH_3)$_3$), 121.9 (s, *m*-C, Mes*), 125.1 (s, *m*-C, Mes*), 128.3 (s, *m*-C, Mes*), 147.9 (s, $C_{arom.}$), 148.6 (s, $C_{arom.}$), 149.1 (s, $C_{arom.}$), 150.8 (m, $C_{arom.}$), 156.7 (m, $C_{arom.}$). ^{19}F NMR (CD_2Cl_2, 282.4 MHz): δ = −162.7 (br, 2 F), −161.5 (br, 2 F), −150.4 (m, 2 F), −129.4 (br, 2 F), −117.3 (br, 2 F). IR (ATR, 32 scans, cm^{-1}): $\tilde{\nu}$ = 2962 (m), 2904 (w), 2868 (w), 1635 (m), 1601 (w), 1589 (w), 1512 (s), 1458 (vs), 1412 (m), 1390 (m), 1362 (s), 1279 (m), 1244 (m), 1211 (m), 1201 (w), 1171 (m), 1138 (m), 1078 (s), 1047 (w), 1018 (m), 974 (vs), 947 (m), 916 (w), 881 (m), 858 (m), 849 (s), 823 (w), 802 (w), 766 (w), 744 (m), 725 (w), 654 (w), 646 (m), 629 (w), 586 (w), 573 (m). Raman (633 nm, 20 s, 20 scans, cm^{-1}): $\tilde{\nu}$ = 3078 (3), 2965 (6), 2953 (6), 2927 (6), 2905 (7), 2878 (4), 2873 (4),

2870 (4), 2867 (4), 2778 (3), 2708 (3), 1637 (7), 1600 (3), 1589 (5), 1463 (4), 1446 (3), 1371 (3), 1360 (2), 1280 (3), 1241 (2), 1202 (2), 1171 (3), 1138 (3), 1124 (3), 1097 (3), 1029 (4), 1024 (4), 1017 (2), 919 (2), 883 (1), 857 (1), 822 (5), 802 (3), 765 (1), 752 (1), 586 (10), 565 (6), 543 (1), 511 (2), 498 (4), 474 (3), 457 (6), 440 (6), 430 (5), 412 (2), 388 (2), 363 (2), 350 (2), 307 (2), 260 (2), 247 (2), 230 (2), 214 (2), 160 (4), 134 (8), 121 (9). MS (CI pos., m/z): 932 [M +H]$^+$, 876 [M +H, –*iso*-butene]$^+$, 475 [Mes*PP(C_6F_5) +H]$^+$, 457 [Mes*NP(C_6F_5)]$^+$, 261 [Mes*NH_2]$^+$.

Single crystals suitable for SC-XRD were obtained as described above.

Figure S9: NMR, IR and Raman spectra of the reaction of Mes*NPCl with DMAP (solvent signals marked by asterisks).

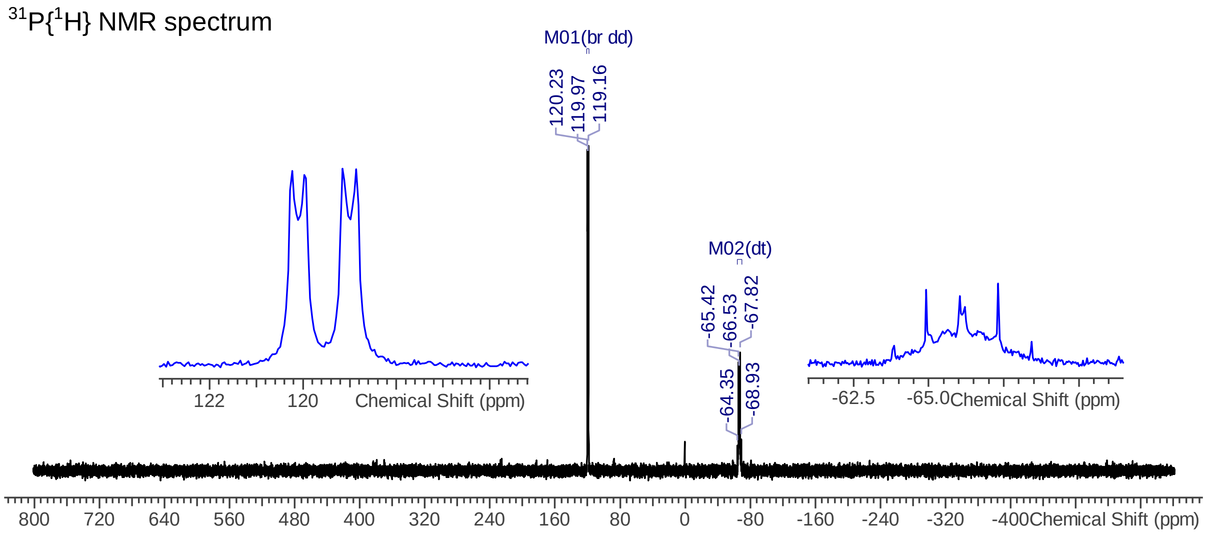

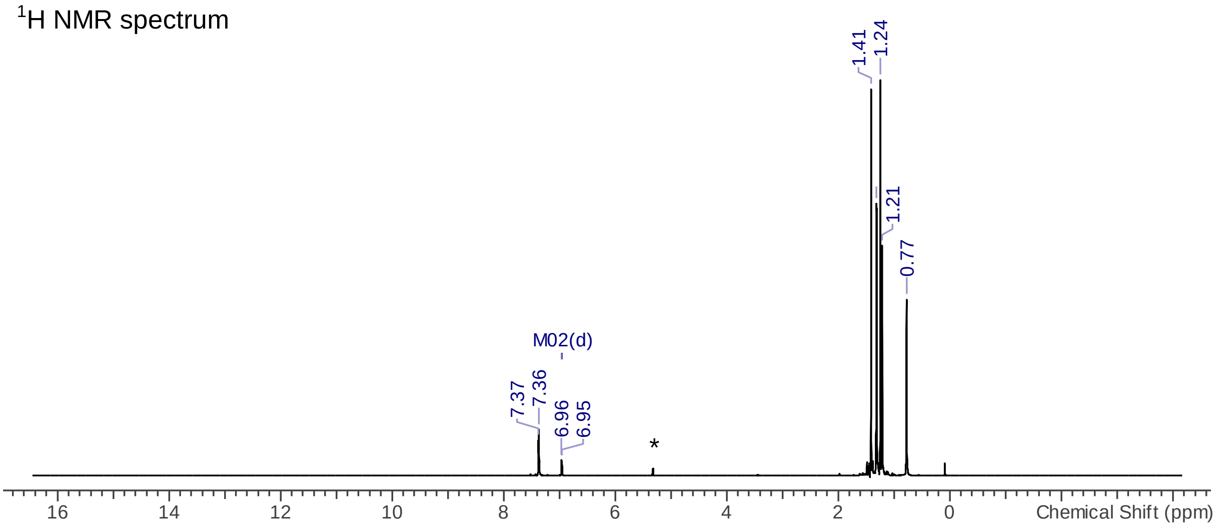

Figure S9 continued.

$^{13}C\{^{1}H\}$ NMR spectrum

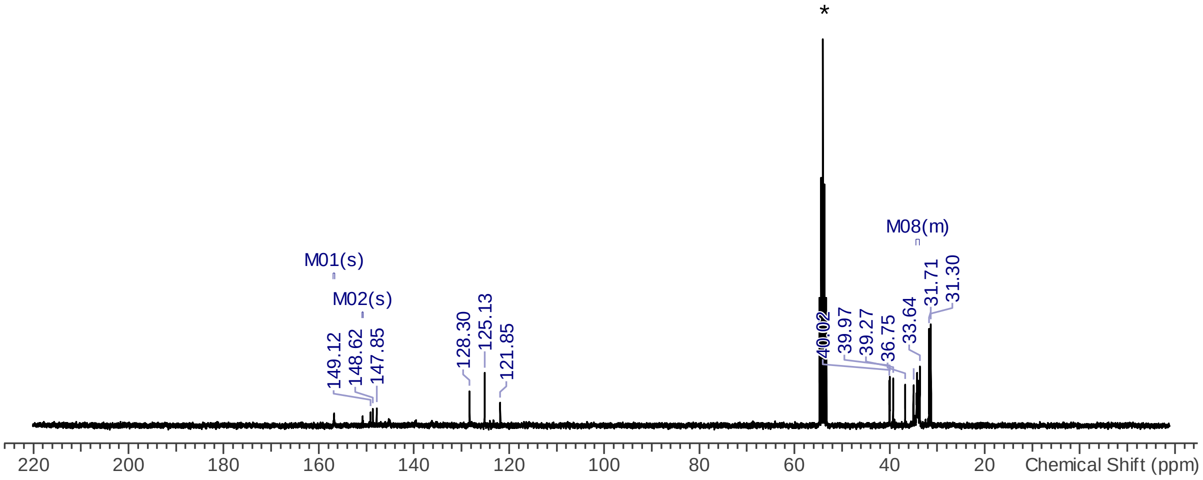

^{19}F NMR spectrum

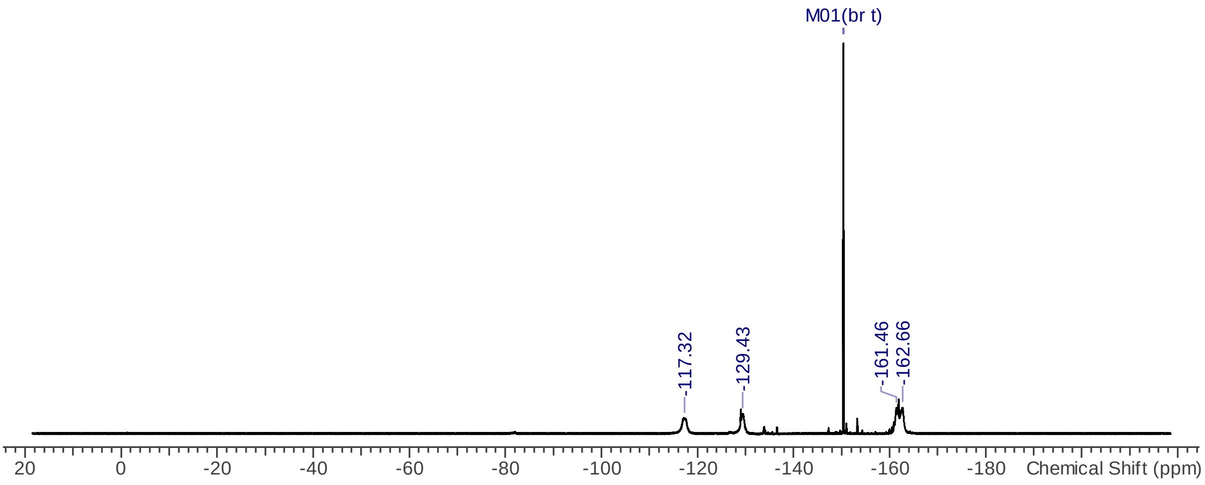

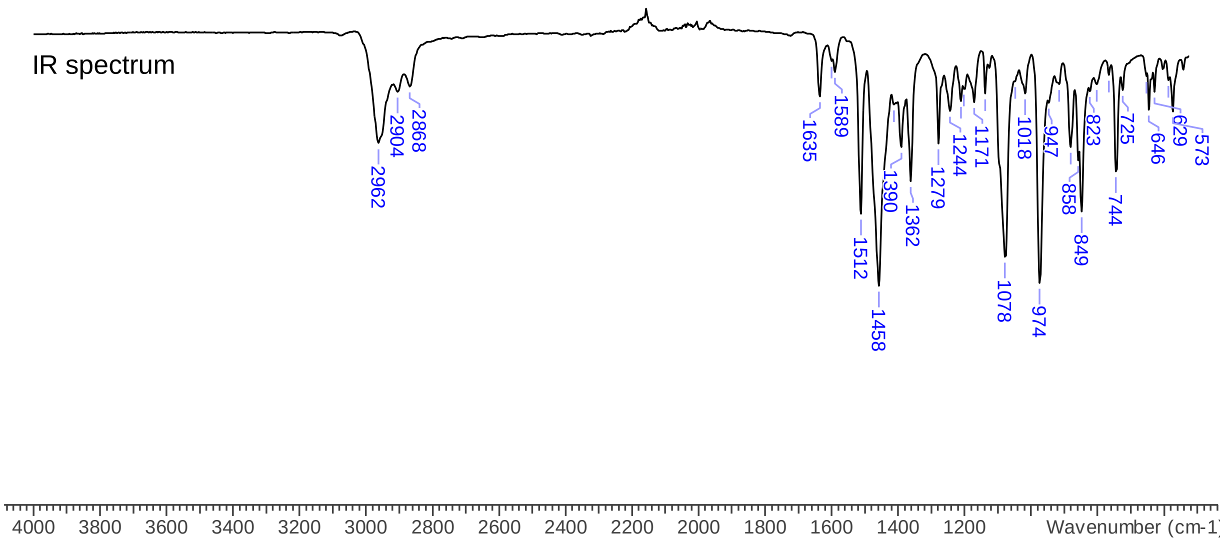

Figure S9 continued.

Raman spectrum

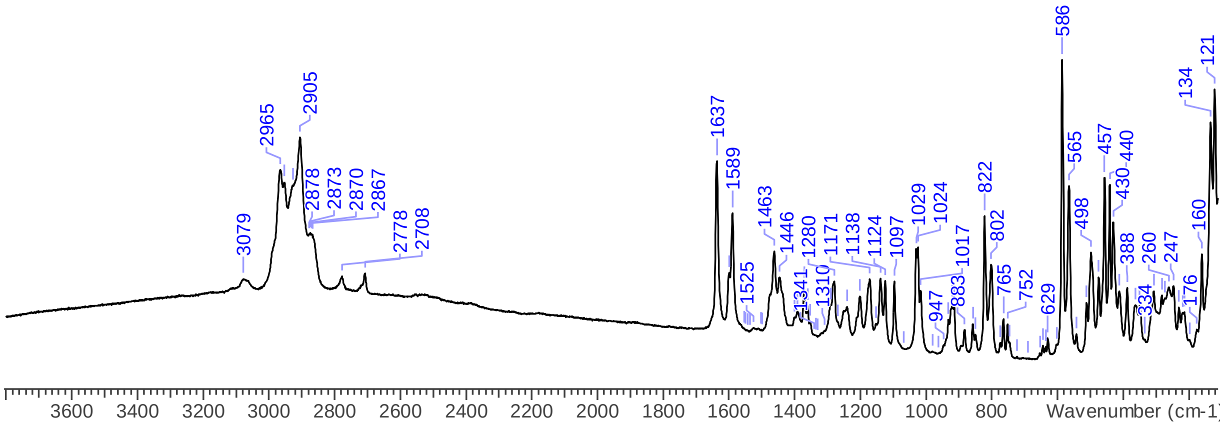

Figure S10: Time-resolved ^{31}P NMR spectra of the dissociation of [Mes*N(μ-P(C_6F_5))$_2$PMes*] (**12**).

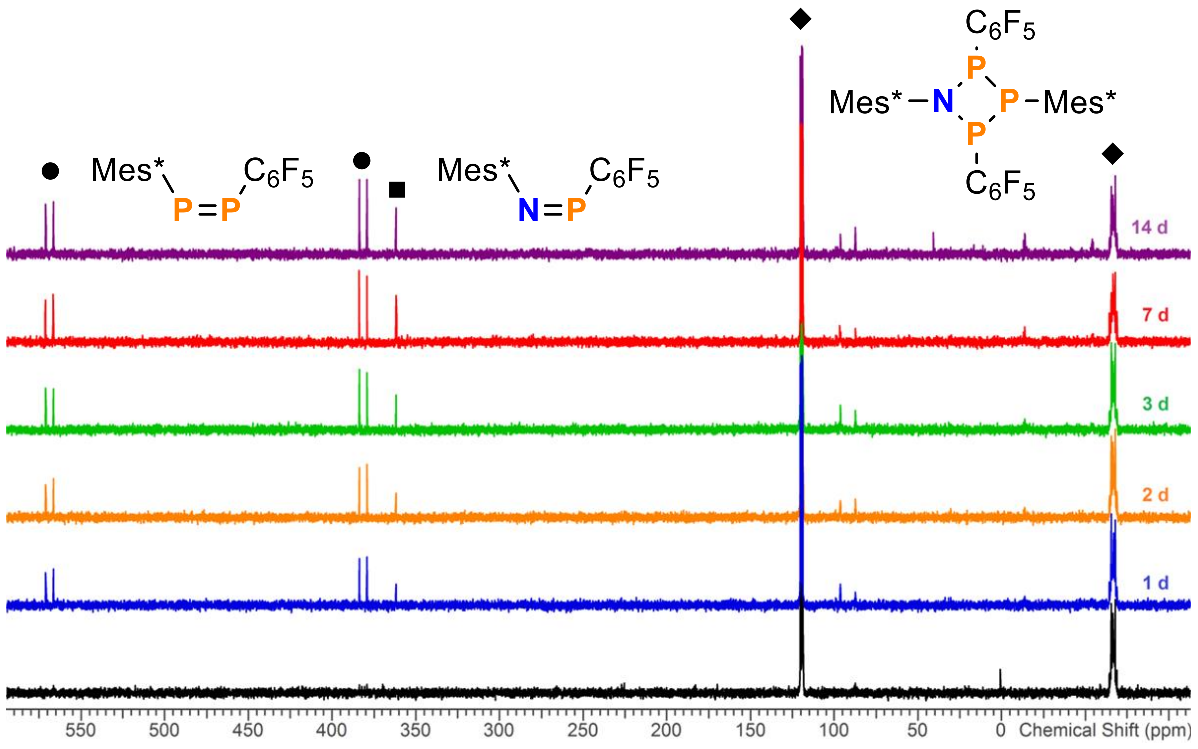

4.5 [(F_5C_6)P(μ-PMes*)]$_2$ (13)

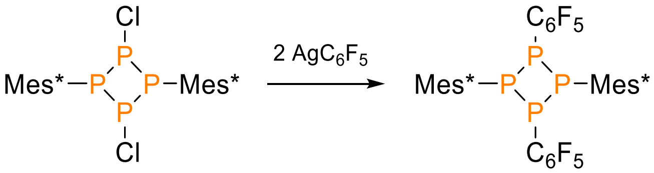

[ClP(μ-PMes*)]$_2$ (206 mg, 0.30 mmol) and AgC_6F_5 (165 mg, 0.60 mmol) are suspended in CH_2Cl_2 (5 mL) at −80 °C. The mixture is stirred and slowly warmed to ambient temperature over a period of five hours. Stirring is continued overnight, and the solvent is removed *in vacuo* the next morning. The solid residue is re-suspended in CH_2Cl_2 (5 mL) and insoluble materials are filtered off. The solvent is evaporated once more. Afterwards, the crude product is crystallized from a minimum of fluorobenzene at ambient temperature. After drying in vacuo, [(F_5C_6)P(μ-PMes*)]$_2$ · ⅓ PhF is obtained. Yield: 130 mg (0.14 mmol, 46 %).

Mp. 169 °C (dec.). CHN calcd. (found) in %: C 61.22 (61.25), H 6.13 (5.81). $^{31}P\{^{1}H\}$ NMR (CD_2Cl_2, 121.5 MHz): δ = −41.2 (m, $^{1}J(^{31}P,^{31}P)$ = −134 Hz, 2 P, *P*Mes*), −15.0 (m, $^{1}J(^{31}P,^{31}P)$ = −134 Hz, 2 P, μ-*P*(C_6F_5)). ^{1}H NMR (CD_2Cl_2, 250.1 MHz): δ = 1.25 (s, 18 H, *o*-*t*Bu), 1.31 (s, 9 H, *p*-*t*Bu), 7.0–7.2 (m, 1 H, PhF), 7.21 (m, 2 H, *m*-H), 7.3−7.4 (m, 0.67 H, PhF). $^{13}C\{^{1}H\}$ NMR (CD_2Cl_2, 125.8 MHz): δ = 31.5 (s, *p*-C(CH_3)$_3$), 34.0 (p, *m*-C(CH_3)$_3$), 35.1 (s, *p*-*C*(CH_3)$_3$), 39.6 (s, *o*-*C*(CH_3)$_3$), 115.8 (d, $J(^{13}C,^{19}F)$ = 21 Hz, PhF) 123.6 (s, *m*-C, Mes*), 124.7 (d, $J(^{13}C,^{19}F)$ = 3 Hz, PhF), 130.6 (d, $J(^{13}C,^{19}F)$ = 8 Hz, PhF), 138.1 (m, $^{1}J(^{13}C,^{19}F)$ = 248 Hz, C_6F_5), 142.6 (m, $^{1}J(^{13}C,^{19}F)$ = 253 Hz, C_6F_5), 148.0 (m, $^{1}J(^{13}C,^{19}F)$ = 249 Hz, C_6F_5), 150.8 (s, *p*-C, Mes*), 157.4 (m, *o*-C, Mes*), 163.5 (d, $^{1}J(^{13}C,^{19}F)$ = 245 Hz, *i*-C, PhF), *ipso*-Cs (Mes*, C_6F_5) not observed. ^{19}F NMR (CD_2Cl_2, 282.4 MHz): δ = −161.8 (m, 2 F, *m*-F), −151.8 (m, 1 F, *p*-F), −127.1 (m, 2 F, *o*-F), −113.9 (s, PhF). IR (ATR, 32 scans, cm^{-1}): $\tilde{\nu}$ = 3090 (vw), 3063 (vw), 2964 (m), 2935 (m), 2904 (w), 2866 (w), 1635 (w), 1593 (m), 1581 (w), 1510 (s), 1495 (m), 1464 (vs), 1392 (m), 1375 (m), 1362 (m), 1279 (m), 1236 (m), 1213 (m), 1176 (w), 1155 (w), 1138 (w), 1122 (m), 1080 (s), 1014 (w), 974 (vs), 943 (w), 918 (w), 893 (m), 876 (m), 818 (m), 804 (m), 754 (s), 741 (m), 723 (w), 685 (m), 650 (w), 644 (w), 627 (w), 596 (m), 582 (m), 575 (w),

538 (w). Raman (633 nm, 40 s, 5 scans, cm^{-1}): $\tilde{\nu}$ = 3072 (1), 2971 (2), 2929 (2), 2907 (2), 2869 (1), 1635 (3), 1592 (4), 1581 (2), 1512 (1), 1495 (1), 1461 (1), 1445 (1), 1397 (1), 1393 (1), 1384 (2), 1377 (3), 1360 (1), 1281 (3), 1241 (1), 1215 (1), 1205 (1), 1183 (2), 1173 (2), 1156 (1), 1138 (1), 1122 (3), 1020 (5), 1011 (7), 1008 (7), 928 (1), 918 (1), 876 (1), 821 (5), 806 (2), 776 (1), 750 (1), 645 (1), 636 (1), 628 (1), 613 (1), 596 (2), 586 (9), 563 (3), 546 (1), 538 (1), 514 (1), 503 (2), 490 (4), 476 (4), 456 (9), 441 (9), 417 (3), 403 (3), 387 (2), 373 (1), 362 (1), 337 (2), 319 (2), 312 (2), 282 (2), 255 (2), 239 (2), 223 (1), 198 (1), 175 (1), 148 (3), 142 (3), 119 (8), 101 (7). MS (CI pos., m/z): 949 [M +H]$^+$, 475 [Mes*PP(C_6F_5) +H]$^+$.

Single crystals suitable of SC-XRD were obtained by crystallization from fluorobenzene.

Figure S11: NMR, IR and Raman spectra of [(F_5C_6)P(μ-PMes*)]$_2$ (solvent signals indicated by asterisks).

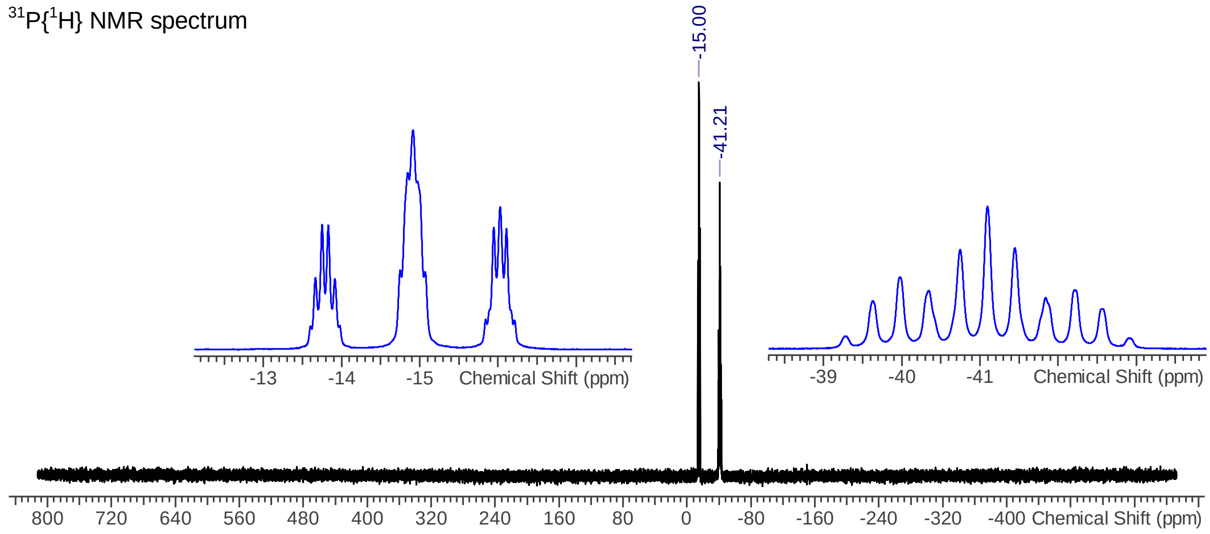

Figure S11 continued.

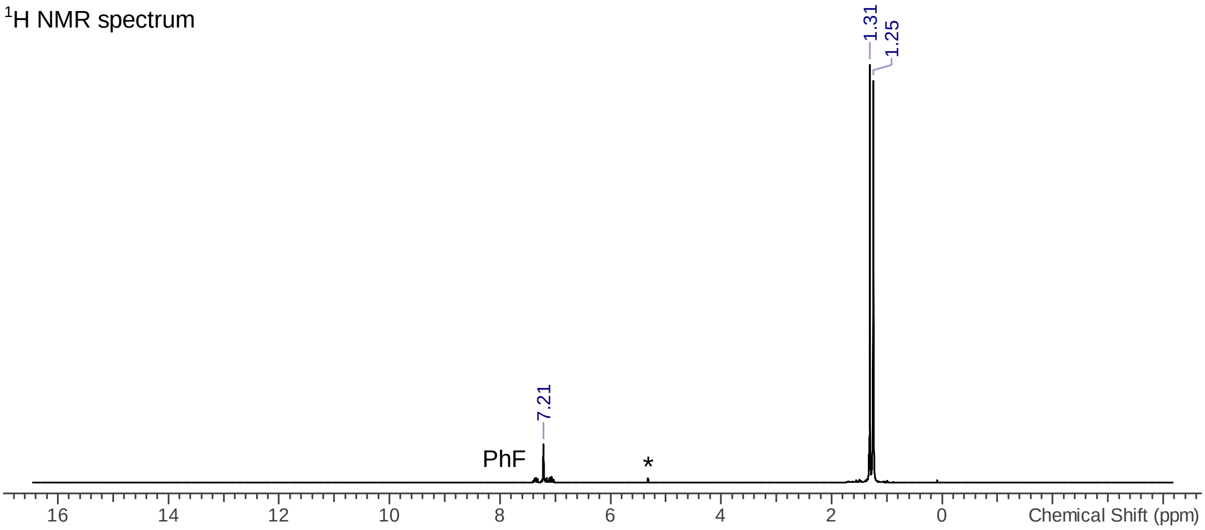

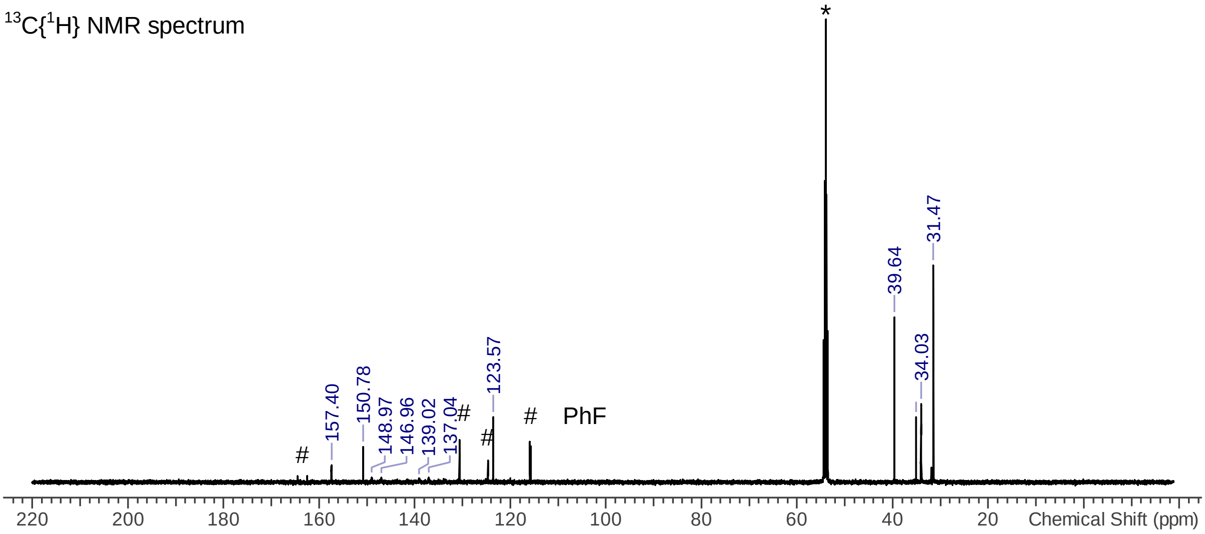

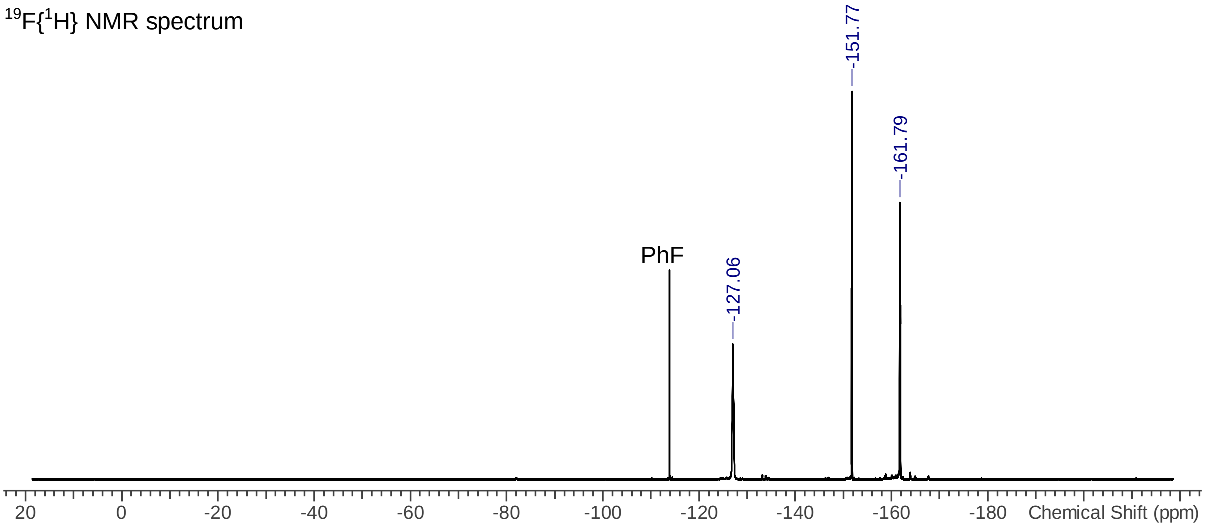

Figure S11 continued.

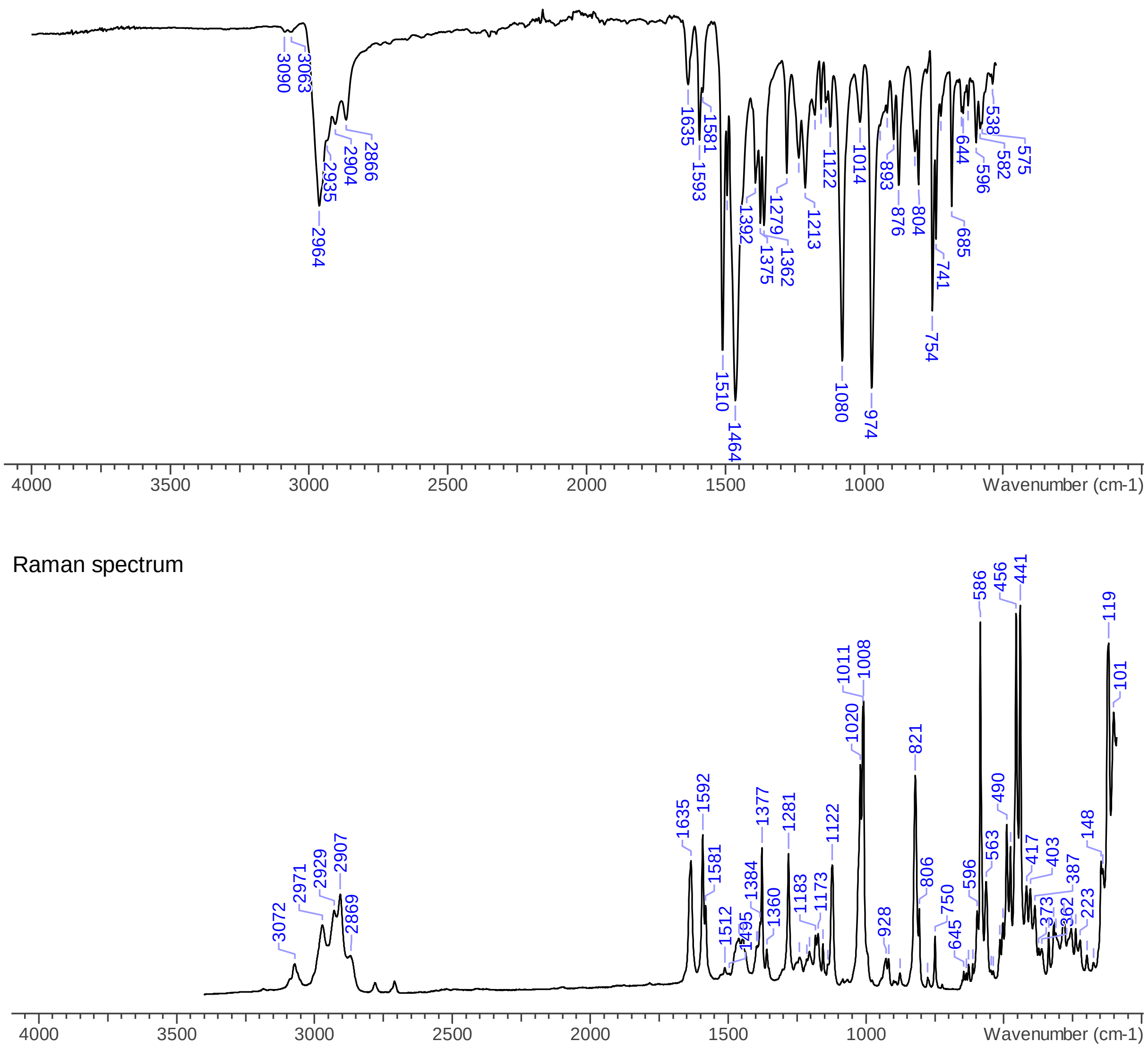

4.6 Mes*$_2$P$_5$N(Mes*)PNMes* (16)

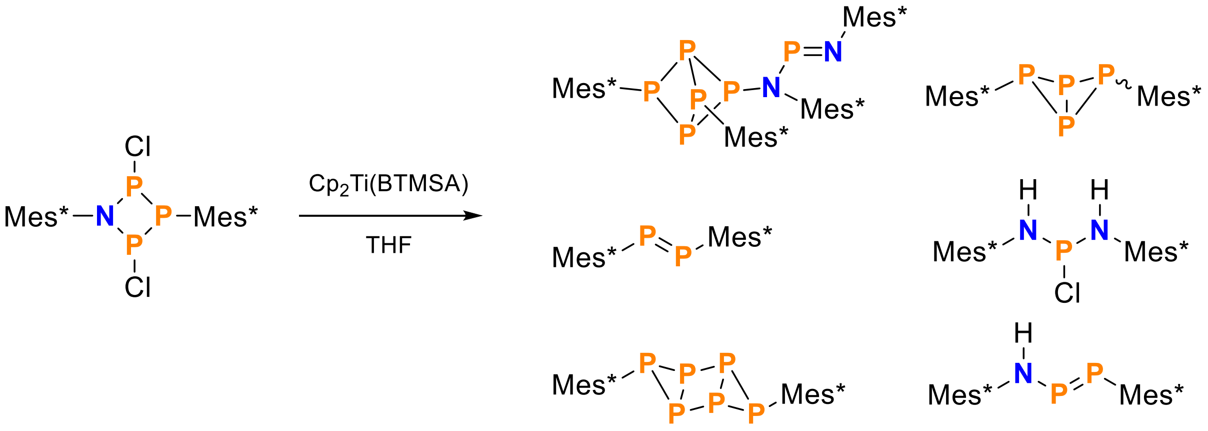

A solution of [Mes*N(μ-PCl)$_2$PMes*] (0.25 g, 0.38 mmol) and Cp$_2$Ti(BTMSA) (0.13 g, 0.38 mmol) in THF (5 mL) is stirred at ambient temperature for 12 h. After removing the volatiles *in vacuo*, the product is extracted with *n*-hexane and the insoluble residue is filtered off. The solvent of the filtrate is evaporated *in vacuo* and the residue is re-dissolved in CH_2Cl_2 and concentrated. Adding CH_3CN until the solution becomes slightly cloudy and subsequent storage in the refrigerator yields a few red crystals, which are washed with cold *n*-hexane.

Only a few small crystals of the product could be isolated. Single crystals were used for SC-XRD (*cf.* pp. S4ff.) and Raman spectroscopy.

Raman (633 nm, 20 s, 20 scans, cm^{-1}): $\tilde{\nu}$ = 3074 (1), 2962 (6), 2923 (7), 2906 (8), 2871 (4), 2778 (1), 2710 (1), 1599 (6), 1462 (4), 1448 (5), 1409 (6), 1390 (4), 1361 (4), 1338 (7), 1302 (4), 1289 (4), 1245 (2), 1202 (4), 1144 (4), 1128 (3), 1110 (2), 1062 (2), 1031 (3), 1002 (2), 932 (4), 923 (4), 854 (2), 837 (3), 823 (9), 798 (2), 780 (2), 767 (2), 748 (2), 732 (1), 704 (2), 644 (1), 568 (7), 556 (7), 505 (2), 498 (3), 456 (3), 443 (4), 417 (4), 412 (4), 407 (4), 387 (5), 375 (5), 355 (6), 285 (5), 254 (7), 188 (3), 175 (4), 135 (10).

Crystallization attempts additionally yielded a few colourless crystals, which were identified as Mes*P$_6$Mes* (**5**, see p. S16).

Some of the red crystals were re-dissolved to obtain a ^{31}P NMR spectrum. Due to the small amounts and instability of the compound, we were unable to record a spectrum

of a pure sample. The ^{31}P NMR signals were assigned on the basis of calculated data and spectrum simulation.

^{31}P NMR (CD_2Cl_2, 121.5 MHz): δ = −25.0 (m, 1 P, *P*Mes*), −21.0 (m, 1 P, *P*Mes*), 26.7 (m, 1 P, *P*NMes*), 200.7 (m, 2 P, bridgehead atoms), 281.3 (m, 1 P, *P*=N). For coupling constants, see Table 2 in the manuscript.

Figure S12: ^{31}P NMR and Raman spectrum of Mes*$_2$P$_5$N(Mes*)PNMes* (product signals indicated by circles, *cf.* Figure 5 in the manuscript).

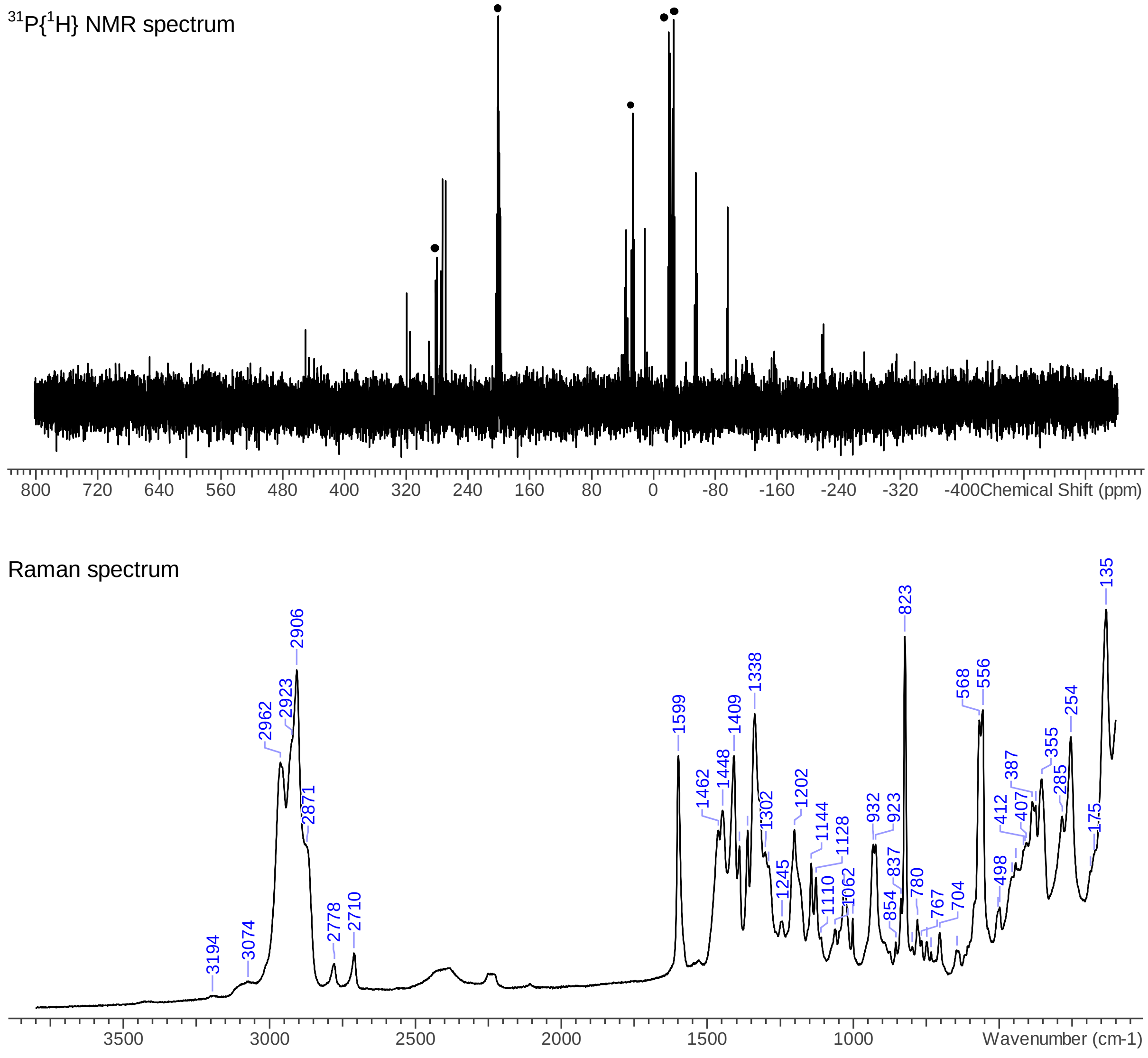

To obtain a better insight into the reaction sequence, *in situ* ^{31}P NMR spectra were recorded after mixing the two reactants.

Figure S13: Time-resolved *in situ* ^{31}P NMR spectra of the reaction of [Mes*N(μ-PCl)$_2$PMes*] (**2**) with Cp_2Ti(BTMSA) in THF-d_8. The signals (except for **16**) were assigned on the basis of literature data.[2,11–15] MesP$_4$Mes* (**15**) and MesPPMes* are reduction products of [Mes*PPCl]$_2$, whereas (Mes*NH)$_2$PCl was previously observed in the reduction of Mes*NPCl. Thus, the mixture of products indicates formal cycloreversion of **2**.

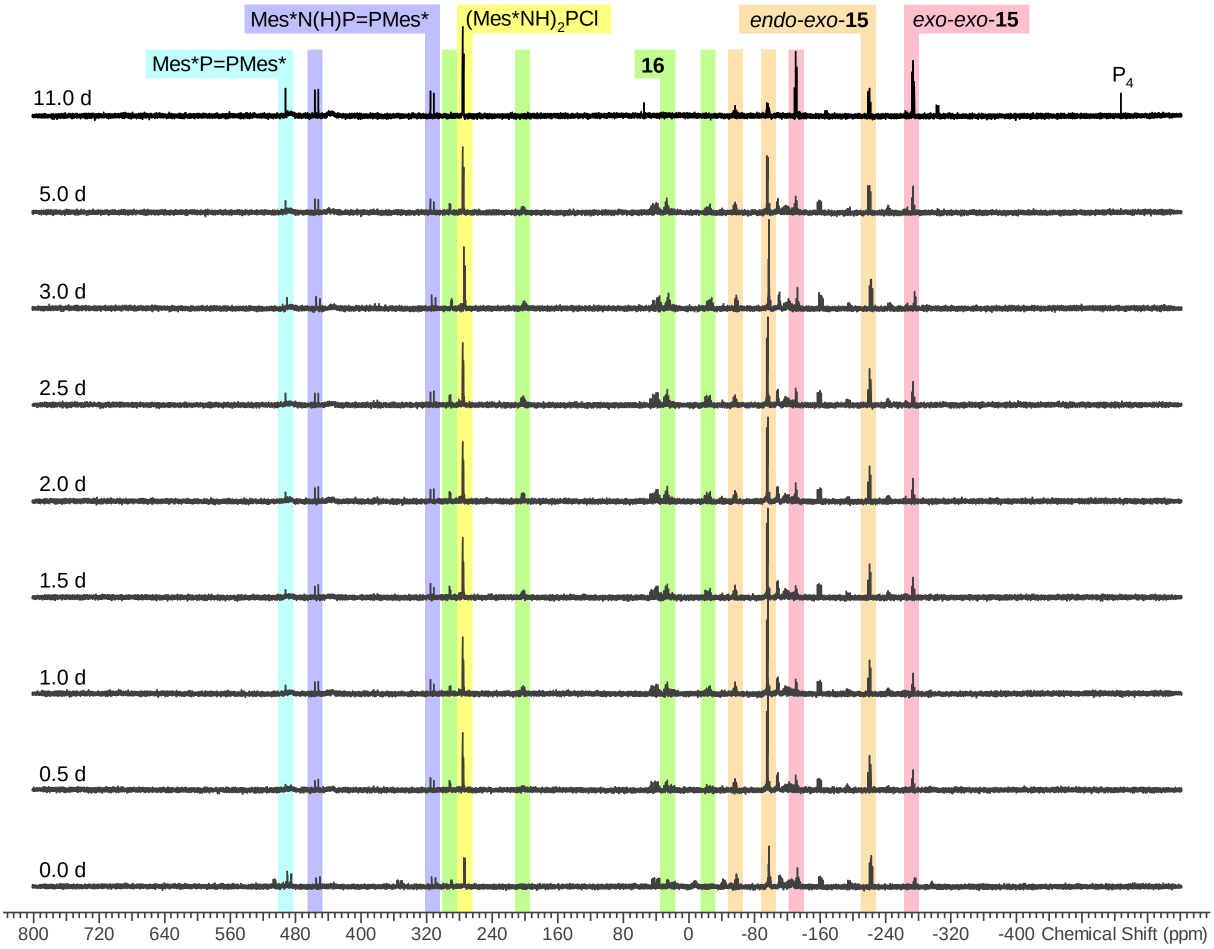

4.7 [Mes*N(μ-PCl)$_2$PMes*] + Mg + Diphenylacetylene

This reaction was performed with the intention to trap the putative biradical Mes*NP$_3$Mes* with diphenylacetylene, by analogy with the reactivity of the biradical [P(μ-NTer)]$_2$.[13]

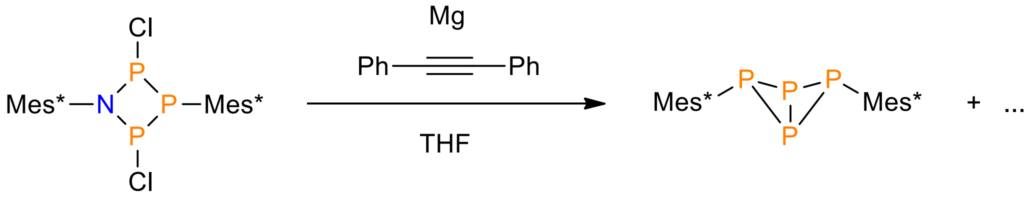

[Mes*N(μ-PCl)$_2$PMes*] (0.10 g, 0.15 mmol), magnesium turnings (0.68 g, 27.89 mmol) and diphenylacetylene (0.07 g, 0.39 mmol) are collectively dissolved in THF (5 mL) and left stirring at ambient temperature for 9 days. The colorless solution begins to turn dark red after 1 day. The solvent is subsequently removed *in vacuo*, the residue is dissolved in *n*-pentane and filtered. The crude filtrate was used for NMR measurements.

^{31}P NMR (CD$_2$Cl$_2$, 121.5 MHz): δ = −273.6 (t, $^{1}J(^{31}P,^{31}P)$ = −177 Hz, 2 P, P (bridgehead), *exo-exo*-Mes*P$_4$Mes*), −131.7 (s), −129.5 (t, $^{1}J(^{31}P,^{31}P)$ = −177 Hz, 2 P, *P*Mes*, *exo-exo*-Mes*P$_4$Mes*).

Figure S14: NMR spectrum of the reaction of [Mes*N(μ-PCl)$_2$PMes*] with Mg and diphenylacetylene.

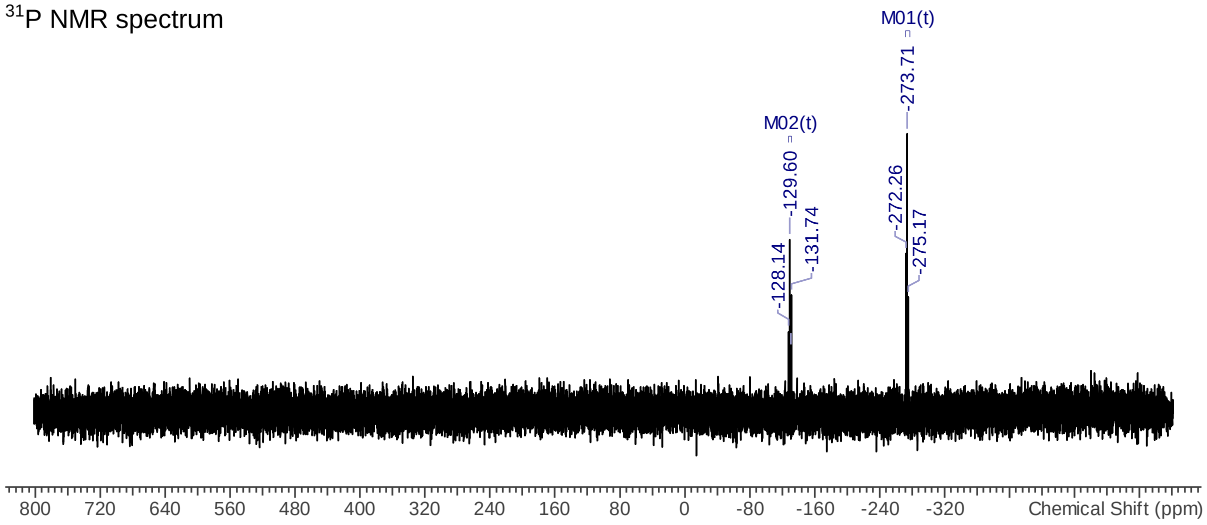

4.8 [Mes*N(μ-PCl)$_2$PMes*] + Mg + Dmp−NC

This reaction was performed with the intention to trap the putative biradical Mes*NP$_3$Mes* with Dmp-NC, by analogy with the reactivity of the biradical [P(μ-NTer)]$_2$.[16]

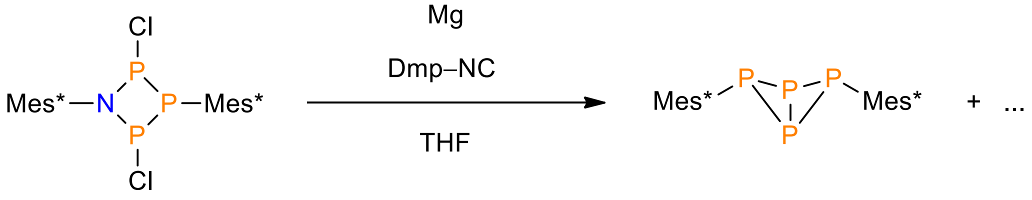

[Mes*N(μ-PCl)$_2$PMes*] (0.10 g, 0.15 mmol), magnesium turnings (0.01 g, 0.25 mmol) and 2,6-dimethylphenyl isocyanide (0.05 g, 0.35 mmol) are collectively dissolved in THF (5 mL) and left stirring for 2 days. The colorless solution begins to turn dark red after 1 day. The solvent is subsequently removed *in vacuo*, the residue is dissolved in *n*-hexane and filtered. The crude filtrate was used for NMR measurements.

^{31}P NMR (CD$_2$Cl$_2$, 121.5 MHz): δ = −273.4 (t, $^{1}J(^{31}P,^{31}P)$ = −177 Hz, 2 P, P (bridgehead), *exo-exo*-Mes*P$_4$Mes*), −128.4 (t, $^{1}J(^{31}P,^{31}P)$ = −177 Hz, 2 P, *P*Mes*, *exo-exo*-Mes*P$_4$Mes*), −120.1 (s), 13.7 (s), 110.0 (d, J = 36 Hz), 114.6 (d, J = 36 Hz), 129.5 (d, J = 10 Hz), 196.7 (d, J = 10 Hz), 203.6 (s), 207.9 (s), 489.0 (s).

Figure S15: NMR spectrum of the reaction of [Mes*N(μ-PCl)$_2$PMes*] with Mg and Dmp−NC.

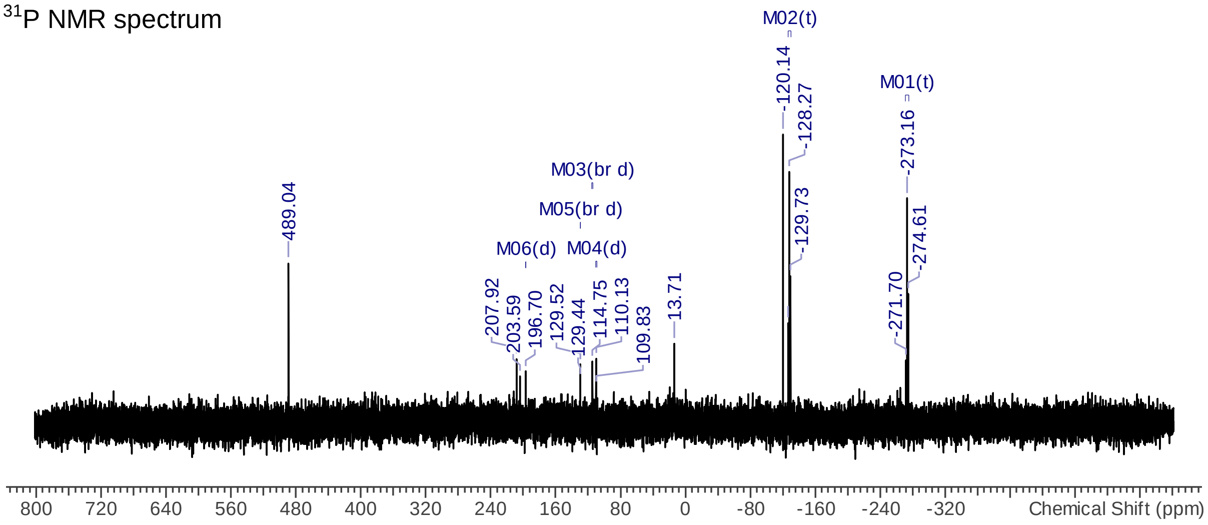

5 Additional spectroscopic data

5.1 ^{31}P NMR data of 12, 13, Mes*PPC$_6$F$_5$, and Mes*NPC$_6$F$_5$

The following tables contain experimental ^{31}P NMR data in comparison with calculated values (GIAO method, PBE0-D3/def2-SVP; in brackets).

Table S4. ^{31}P NMR data of [Mes*N(μ-P(C$_6$F$_5$))$_2$PMes*] (**12**).

	***δ* [ppm]**	***J* [Hz]**
A	−66.8 (−77.9)	
X	+119.6 (+74.4)	J_{AX} = −133 (−129)

Table S5. ^{31}P NMR data of [(F$_5$C$_6$)P(μ-PMes*)]$_2$ (**13**).

	***δ* [ppm]**	***J* [Hz]**
A	−41.2 (−59.0)	
X	−15.0 (−46.1)	J_{AX} = −134 (−124)

Table S6. ^{31}P NMR data of (*Z*)-Mes*P=PC$_6$F$_5$.

	***δ* [ppm]**	***J* [Hz]**
A	+381.2 (+373.3)	
X	+568.7 (+525.8)	J_{AX} = −557 (−507)

Table S7. ^{31}P NMR data of (*Z*)-Mes*N=PC$_6$F$_5$.

	***δ* [ppm]**
A	+361.7 (+372.8)

6 Computational Details

6.1 General remarks

Computations were carried out using Gaussian09[17] or ORCA 4.1.1[18] and the standalone version of NBO 6.0.[19–22]

Structure optimizations were done using the hybrid DFT functional PBE0[23–25] in conjunction with Grimme's dispersion correction D3(BJ)[26,27] and the def2-SVP basis set[28] (notation PBE0-D3/def2-SVP). All structures were fully optimized and confirmed as minima by frequency analyses. Calculated frequencies were scaled by 0.950 (as derived from Truhlar's Reduced Scale Factor Optimization model).[29] Partial charges were determined by Natural Population analysis using the NBO program. Chemical shifts and coupling constants were derived by the GIAO method.[30–34] The calculated absolute shifts ($\sigma_{calc,X}$) were referenced to the experimental absolute shift of 85 % H_3PO_4 in the gas phase ($\sigma_{ref,1}$ = 328.35 ppm),[35] using PH_3 ($\sigma_{ref,2}$ = 594.45 ppm) as a secondary standard:[36]

$$\begin{aligned}\delta_{calc,X} &= (\sigma_{ref,1} - \sigma_{ref,2}) - (\sigma_{calc,X} - \sigma_{calc,PH_3}) \\ &= \sigma_{calc,PH_3} - \sigma_{calc,X} - 266.1\ \text{ppm}\end{aligned}$$

At the PBE0-D3/def2-SVP level of theory, σ_{calc,PH_3} amounts to +629.17 ppm. Spin-spin coupling constants were calculated in a two-step process, using a modified basis set for computation of the Fermi contact term.[37]

For structure optimizations involving biradicals, the pure DFT method PBE[23,24] was applied (PBE-D3/def2-SVP), as pure DFT methods are more robust towards multi-determinantal systems.[38–40] All thermal corrections to the total energy were computed at the same level. More accurate estimates of the electronic energy were obtained by single-point DLPNO-CCSD(T)/def2-TZVP[41–43] computations (notation

DLPNO-CCSD(T)/def2-TZVP//PBE-D3/def2-SVP). The T_1 diagnostic was evaluated in each case to ensure reliable results. (Empirically, CCSD(T) results with T_1 values smaller than 0.02 are considered reliable.)[39]

Biradical characters of the N_2P_2, NP_3 and P_4 biradicals discussed in the manuscript were estimated using CAS(6,4)/def2-TZVP[44–52] single-point calculations at the PBE-D3/def2-TZVP geometries. The biradical character was estimated using the β scale introduced by Salem[53] and later refined by Miliordos *et al.*[54]

$$\beta = \frac{2c_2^2}{c_1^2 + c_2^2}$$

where c_1 and c_2 refer to the coefficients of the configurations with the largest and second-largest weights in the CI expansion.

It should be emphasized that all computations were carried out for single, isolated gas phase molecules. There may be significant differences between gas phase, solution and solid state.

6.2 NMR data of [$Mes^*_2NP_3Cl$][$GaCl_4$] (6)

When investigating the theoretical structure of the azatriphosphenium cation of **6**, it became clear that there are two possible minimum structures with similar energy. Both structures differ in the arrangement of one Mes* substituent (rotation about a P–C single bond). Thus, it can be expected that the system is highly dynamic in solution and a fast exchange between both isomers is observed in the NMR spectrum. Consequently, the predicted ^{31}P NMR shifts were calculated as a weighted average of both isomers using a Boltzmann distribution at 203 K (Table S8).

Table S8. Calculated ^{31}P NMR data of both isomers of the azatriphosphenium cation $[Mes^*{}_2NP_3Cl]^+$.

Isomer	ΔG_{203} [kJ/mol]	x_i	δ_A [ppm]	δ_M [ppm]	δ_X [ppm]	J_{AM} [Hz]	J_{AX} [Hz]	J_{MX} [Hz]
#1	0.0	0.677	107.9	119.8	423.5	−259	+60	−442
#2	1.2	0.323	101.2	139.9	483.0	−301	+89	−442
weighted average	–	–	105.7	126.3	442.7	−273	+69	−442
exptl.	–	–	114.1	175.4	446.4	−273	+107	−470

6.3 NMR data of Mes*₂P₅N(Mes*)PNMes* (16)

NMR shifts and coupling constants of **16** were computed using a single-point PBE0-D3/def2-TZVP calculation at the PBE0-D3/def2-SVP geometry (σ_{calc,PH_3} = +572.04 ppm at PBE0-D3/def2-TZVP).

6.4 Isomers of P_5H_3

In total, eight different structures of P_5H_3 were considered (Figure S16). For more details on electronic energies, see Table S9.

Figure S16: Isomers of P_5H_3 considered in this study ($\Delta G°$ in kJ/mol, DLPNO-CCSD(T)/def2-TZVP//PBE0-D3/def2-TZVP).

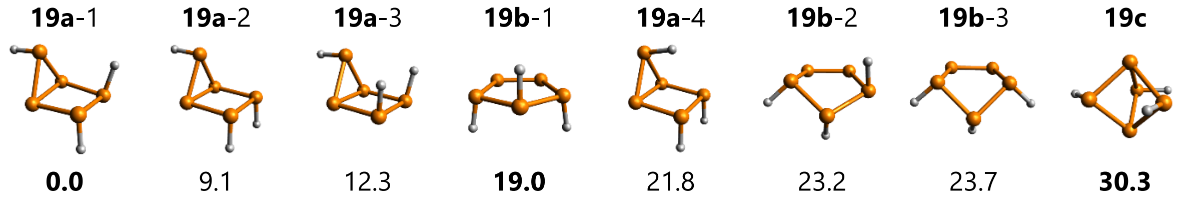

6.5 Summary of calculated data

Table S9. Summary of calculated data, including electronic energies and thermal corrections.

Compd.	PG	Opt. method	E_{tot}[a]	ΔG[b]	$E_{CCSD(T)}$[c]	T_1
**6** (isomer 1)	C_1	PBE0-D3 def2-SVP	−2941.9236	0.8269[d] 0.7910		
**6** (isomer 2)	C_1		−2941.9227	0.8265[d] 0.7901		
**7**	C_1		−3228.3943	0.7893		
**8**	C_1		−3228.4118	0.7848		
**11**	C_1		−2941.8968	0.7893		
**12**	C_1		−3935.3242	0.8724	−3935.1238	0.0104
**13**	C_1		−4221.8076	0.8645		
Mes*PPC_6F_5	C_1		−2110.8739	0.4220	−2110.6603	0.0108
Mes*NPC_6F_5	C_1		−1824.3870	0.4246	−1824.4157	0.0109
**16**	C_1		−4964.5067	1.6087		
Mes*$_2NP_3$ bicyclic	C_1	PBE-D3 def2-SVP	−2481.8703	0.7574	−2481.3739	0.0103
Mes*$_2NP_3$ biradical	C_1		−2481.8724	0.7614	−2481.3726	0.0099
$H_2N_2P_2$ bicyclic	C_{2v}	PBE-D3 def2-TZVP	−793.0273	0.0051	−792.3069	0.0183
$H_2N_2P_2$ biradical	D_{2h}		−793.0521	0.0056	−792.3364	0.0117
H_2NP_3 bicyclic	C_S		−1079.5487	−0.0016	−1078.5637	0.0179
H_2NP_3 biradical	C_S		−1079.5348	−0.0020	−1078.5480	0.0158
H_2P_4 bicyclic	C_{2v}		−1366.0784	−0.0068	−1364.8288	0.0173
H_2P_4 biradical	C_{2h}		−1366.0352	−0.0098	−1364.7806	0.0168

[a] Total SCF energy in a.u.; [b] thermal correction to Gibbs energy in a.u. (298 K unless stated otherwise); [c] single-point DLPNO-CCSD(T)/def2-TZVP energy; [d] 203 K.

Table S9 continued.

Compd.	PG	Opt. method	E_{tot}[a]	ΔG[b]	$E_{CCSD(T)}$[c]	T_1
**19a**-1	C_1	PBE0-D3 def2-TZVP	−1708.0318	0.0016	−1706.3308	0.0170
**19a**-2	C_S		−1708.0277	0.0013	−1706.3271	0.0170
**19a**-3	C_S		−1708.0255	0.0004	−1706.3248	0.0174
**19a**-4	C_S		−1708.0226	0.0011	−1706.3220	0.0176
**19b**-1	C_S		−1708.0196	0.0008	−1706.3227	0.0188
**19b**-2	C_1		−1708.0178	0.0007	−1706.3211	0.0186
**19b**-3	C_S		−1708.0184	0.0012	−1706.3214	0.0181
**19c**	C_{3h}		−1708.0212	0.0033	−1706.3209	0.0175

[a] Total SCF energy in a.u.; [b] thermal correction to Gibbs energy in a.u. (298 K unless stated otherwise); [c] single-point DLPNO-CCSD(T)/def2-TZVP energy.

6.6 Optimized structures (.xyz-files)

6.6.1 PH_3

```
4
PH3 @ PBE0-D3/def2-SVP
P           0.00000         0.00000         0.13012
H           0.00000         1.19618        -0.65060
H           1.03592        -0.59809        -0.65060
H          -1.03592        -0.59809        -0.65060
```

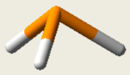

```
4
PH3 @ PBE0-D3/def2-TZVP
P           0.00000         0.00000         0.12903
H           0.00000         1.18991        -0.64515
H           1.03049        -0.59496        -0.64515
H          -1.03049        -0.59496        -0.64515
```

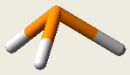

6.6.2 [Mes*$_2$NP$_3$Cl]$^+$ (cation of salt 6)

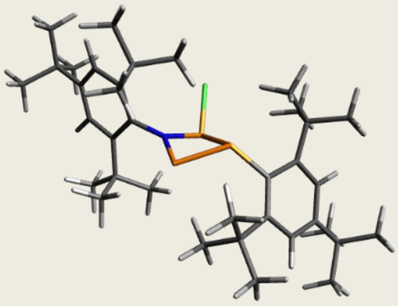

```
99
cation 6, isomer 1 @ PBE0-D3/def2-SVP
P          -0.26072       -0.66816        1.74732
P           1.12999        0.79310        1.00527
P          -0.16924        0.37181       -0.79228
N          -1.24776       -0.18953        0.47889
Cl         -1.05629        2.09189       -1.47710
C           6.95894       -2.15143       -1.13732
C           4.19814       -2.64560        2.62392
C           7.57663        0.25542       -1.29471
C           6.45030       -0.76342       -1.52766
C           2.03650       -2.77352        1.45651
C           6.06508       -0.78065       -3.01490
C           4.68586       -1.14749        0.29535
C           3.04298       -1.79028        2.07572
C           5.22594       -0.35178       -0.71529
C           3.52169       -0.81657        0.98415
C           2.50981       -1.03244        3.30240
C           4.62655        0.88544       -0.93152
C          -2.21685       -3.37310        0.62391
C           2.86027        0.39529        0.62682
C          -3.21290       -3.67903       -1.61150
C          -1.02135       -2.59991       -1.44769
C           3.49182        1.33138       -0.24973
C          -2.38740       -2.70992       -0.75287
C           4.38193        3.66140       -0.31716
C           3.11281        2.80911       -0.52924
C          -3.14165       -1.37383       -0.53690
C           2.64266        2.94498       -1.98519
C           2.05450        3.43595        0.38709
C          -2.66721       -0.30674        0.27488
C          -4.44666       -1.25833       -1.01296
C          -2.93886        0.83356        3.18392
C          -3.52622        0.70898        0.76473
C          -7.48529       -1.35778       -0.91706
C          -5.28863       -0.19173       -0.69137
C          -6.53529       -0.05093       -2.84109
C          -4.82213        0.73383        0.23619
C          -3.18605        1.67743        1.92150
C          -6.68154       -0.11001       -1.31311
C          -1.99488        2.60541        1.65784
C          -4.36853        2.60034        2.24814
C          -7.44658        1.12970       -0.84876
H           7.82566       -2.41574       -1.76037
H           7.28859       -2.18985       -0.08785
H           6.19431       -2.92826       -1.29172
H           4.58192       -3.36770        1.89019
H           8.46966       -0.03174       -1.87033
H           3.83441       -3.22977        3.48219
H           2.54783       -3.41524        0.72395
H           7.85668        0.30041       -0.23136
H           1.57548       -3.41661        2.22148
H           5.19066       -2.07404        0.55180
H           6.93174       -1.07466       -3.62631
H           5.03436       -2.02123        2.97128
H           5.25379       -1.49961       -3.20570
H           1.22240       -2.28091        0.89590
```

```
H          7.29028          1.26881         -1.61280
H          2.03955         -1.72654          4.01450
H          5.73159          0.20738         -3.36559
H         -1.73200         -4.35607          0.51826
H         -2.66573         -4.62801         -1.70509
H         -0.67865         -3.60523         -1.73532
H          3.34116         -0.52986          3.81912
H          1.78029         -0.23382          3.09490
H          5.08331          1.55682         -1.65774
H         -3.19110         -3.52114          1.11216
H          5.20336          3.39136         -0.99257
H         -1.59532         -2.77595          1.31011
H          4.74946          3.56823          0.71589
H         -0.23374         -2.18665         -0.80257
H         -4.18791         -3.91122         -1.16101
H         -3.37831         -3.29221         -2.62812
H         -1.07605         -1.99452         -2.36451
H          4.14554          4.71994         -0.50025
H          3.41438          2.61929         -2.69701
H          2.25532          3.25193          1.45435
H         -4.83727         -2.03930         -1.66042
H          1.73820          2.34844         -2.17561
H          1.02559          3.12821          0.15413
H          2.07486          4.52682          0.24264
H          2.39876          3.99471         -2.20913
H         -3.79718          0.17887          3.39612
H         -7.01000         -2.28464         -1.27114
H         -2.05139          0.18419          3.10100
H         -6.02939         -0.94244         -3.24071
H         -2.77661          1.48636          4.05550
H         -7.59256         -1.42827          0.17611
H         -8.49373         -1.31464         -1.35629
H         -1.05347          2.07870          1.46414
H         -5.95607          0.83301         -3.14883
H         -7.52671          0.00887         -3.31577
H         -5.26470          2.04281          2.55577
H         -5.49552          1.51968          0.56156
H         -4.08735          3.25320          3.08693
H         -1.82695          3.23870          2.54220
H         -2.19203          3.26592          0.80278
H         -7.62033          1.12439          0.23816
H         -4.63224          3.25084          1.40128
H         -8.43210          1.15918         -1.33594
H         -6.92023          2.05969         -1.11292
```

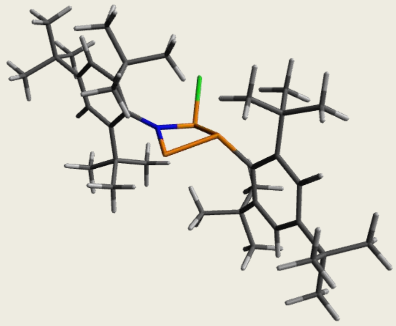

```
99
cation 6, isomer 2 @ PBE0-D3/def2-SVP
P          1.16827         -0.57070          0.39743
P          0.02761          0.17067         -1.23060
P         -0.56369          0.46608          1.42885
C          2.90929         -0.10383          0.37652
C          3.43379          1.21650          0.23512
C          3.76642         -1.24900          0.27955
C          4.74306          1.32742         -0.23255
C          5.06472         -1.03356         -0.17739
C          5.56048          0.22924         -0.50033
H          5.15554          2.31927         -0.38399
H          5.72703         -1.88849         -0.28838
```

C	-2.69414	0.11277	-0.38665
C	-3.28783	-1.16883	-0.56222
C	-3.48326	1.29798	-0.32240
C	-4.68293	-1.23082	-0.48188
C	-4.86515	1.12439	-0.24751
C	-5.49166	-0.11992	-0.27428
H	-5.16120	-2.19874	-0.58094
H	-5.49414	2.00675	-0.16986
C	2.72548	2.50659	0.70803
C	1.49785	2.88892	-0.13229
C	2.34878	2.32954	2.18790
C	3.66421	3.72083	0.64255
H	1.70976	2.83289	-1.21066
H	0.61627	2.27296	0.08945
H	1.19342	3.92117	0.09715
H	3.25268	2.20188	2.80147
H	1.80750	3.21501	2.55503
H	1.71202	1.45284	2.36611
H	3.14876	4.58899	1.07799
H	4.58664	3.56482	1.21953
H	3.93502	3.98666	-0.39006
C	3.36394	-2.69147	0.67925
C	2.64253	-2.70200	2.03874
C	2.51211	-3.36348	-0.41181
C	4.60892	-3.57318	0.86502
H	3.26442	-2.23152	2.81497
H	1.66494	-2.19276	2.03615
H	2.44206	-3.74030	2.34259
H	3.03261	-3.33823	-1.38097
H	2.33418	-4.41751	-0.14847
H	1.52759	-2.89244	-0.54106
H	4.29551	-4.54948	1.26161
H	5.13356	-3.76888	-0.08099
H	5.32200	-3.13580	1.57935
C	6.97268	0.37063	-1.06073
C	7.06650	-0.43661	-2.36488
C	7.33176	1.82610	-1.36173
C	7.97575	-0.18132	-0.03558
H	6.86051	-1.50524	-2.20460
H	6.35340	-0.06229	-3.11514
H	8.07939	-0.35374	-2.78750
H	7.33210	2.44888	-0.45411
H	8.34369	1.87434	-1.78939
H	6.64399	2.27627	-2.09392
H	9.00163	-0.07903	-0.42093
H	7.91298	0.36860	0.91569
H	7.80688	-1.24734	0.17668
C	-2.54082	-2.47294	-0.93554
C	-1.87998	-2.29039	-2.31165
C	-1.51088	-2.92340	0.10540
C	-3.50706	-3.65681	-1.09426
H	-2.61678	-1.98377	-3.06893
H	-1.08298	-1.52636	-2.31657
H	-1.41242	-3.23092	-2.64132
H	-1.99171	-3.12561	1.07213
H	-1.02929	-3.85305	-0.23448
H	-0.71045	-2.19644	0.27822
H	-2.92732	-4.54866	-1.37218
H	-4.03444	-3.89017	-0.15793
H	-4.25161	-3.49312	-1.88648

```
C          -2.95408          2.75278         -0.42023
C          -2.30027          3.23436          0.88264
C          -1.99781          2.90386         -1.61162
C          -4.10321          3.73941         -0.69505
H          -2.96568          3.06336          1.74217
H          -1.34309          2.74277          1.09702
H          -2.09676          4.31466          0.82354
H          -2.47544          2.56790         -2.54403
H          -1.71791          3.96069         -1.73696
H          -1.05866          2.34854         -1.49535
H          -3.67610          4.73900         -0.86026
H          -4.67773          3.47021         -1.59336
H          -4.79650          3.82598          0.15359
C          -7.00854         -0.21445         -0.12500
C          -7.50169         -1.66065         -0.17798
C          -7.40780          0.38943          1.22986
C          -7.67847          0.57315         -1.26100
H          -7.27892         -2.13690         -1.14506
H          -7.06101         -2.27472          0.62234
H          -8.59333         -1.68383         -0.04678
H          -7.11714          1.44747          1.31030
H          -8.49908          0.33382          1.36370
H          -6.93422         -0.15564          2.06063
H          -8.77377          0.51006         -1.17091
H          -7.40634          1.63887         -1.24121
H          -7.39389          0.16892         -2.24441
Cl         -1.52716         -0.81918          2.69288
N          -1.27076          0.19218         -0.15010
```

6.6.3 [Mes*$_2$P$_4$Cl]$^+$ (cation of salt 7)

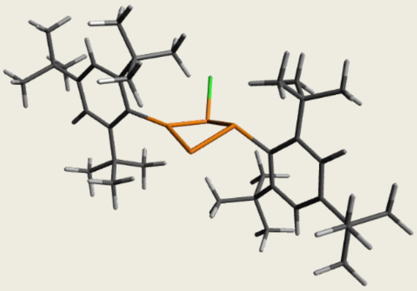

```
99
cation 7 @ PBE-D3/def2-SVP
P           1.37242         -0.68557          0.33181
P           0.45156          0.25473         -1.41130
P          -1.24299          0.21600         -0.26464
P          -0.22594          0.28552          1.63508
C           3.10097         -0.12804          0.41606
C           3.60842          1.20545          0.33680
C           3.98163         -1.24554          0.23112
C           4.88720          1.36668         -0.19660
C           5.24905         -0.98410         -0.28777
C           5.70139          0.30099         -0.58129
H           5.27807          2.37291         -0.30692
H           5.91890         -1.82083         -0.47178
C          -3.01032          0.14618         -0.50494
C          -3.61204         -1.14298         -0.63470
C          -3.79122          1.33719         -0.37207
C          -4.99636         -1.20474         -0.46693
C          -5.16273          1.16405         -0.21915
C          -5.78704         -0.08617         -0.21538
H          -5.48177         -2.17223         -0.53973
H          -5.78665          2.04626         -0.10213
C           2.92984          2.44625          0.95991
C           1.64734          2.89121          0.24660
C           2.65693          2.13013          2.43952
C           3.85846          3.66940          0.94104
H           1.80736          2.99849         -0.83693
```

H	0.80426	2.20930	0.40620
H	1.32467	3.86778	0.63831
H	3.60106	1.94496	2.97275
H	2.14717	2.97571	2.92665
H	2.03027	1.23833	2.57328
H	3.36776	4.49237	1.48074
H	4.81675	3.47420	1.44279
H	4.06182	4.02600	-0.07976
C	3.64910	-2.70986	0.62252
C	2.92538	-2.75946	1.97961
C	2.84387	-3.43431	-0.47082
C	4.94290	-3.51821	0.81924
H	3.52401	-2.26461	2.75912
H	1.92761	-2.29327	1.97349
H	2.77140	-3.80704	2.27925
H	3.35987	-3.36524	-1.44042
H	2.74613	-4.50065	-0.21466
H	1.82995	-3.02958	-0.59318
H	4.68506	-4.50080	1.24002
H	5.47286	-3.70843	-0.12490
H	5.63524	-3.02508	1.51756
C	7.06903	0.49839	-1.22894
C	7.07890	-0.23216	-2.58074
C	7.38615	1.97449	-1.47086
C	8.15085	-0.09314	-0.31208
H	6.89506	-1.31075	-2.46700
H	6.30955	0.17348	-3.25540
H	8.05805	-0.11116	-3.06892
H	7.44446	2.54460	-0.53109
H	8.36302	2.06466	-1.96787
H	6.63952	2.45322	-2.12292
H	9.14551	0.04967	-0.76135
H	8.14781	0.39907	0.67235
H	8.01213	-1.17244	-0.15114
C	-2.86435	-2.42790	-1.05195
C	-2.27640	-2.20918	-2.45723
C	-1.77509	-2.84480	-0.05430
C	-3.81577	-3.62742	-1.15445
H	-3.07394	-1.97815	-3.17887
H	-1.54912	-1.38734	-2.49388
H	-1.76256	-3.12149	-2.79672
H	-2.19227	-2.98989	0.95171
H	-1.32426	-3.79464	-0.37964
H	-0.93833	-2.13336	0.02892
H	-3.24169	-4.50669	-1.47940
H	-4.27783	-3.87687	-0.18786
H	-4.61174	-3.46847	-1.89596
C	-3.23406	2.77358	-0.47280
C	-2.36297	3.14121	0.73796
C	-2.44634	2.92577	-1.78388
C	-4.36456	3.81011	-0.52297
H	-2.89634	2.95417	1.68224
H	-1.41307	2.59491	0.77081
H	-2.10720	4.21088	0.69764
H	-3.08858	2.71432	-2.65164
H	-2.07203	3.95627	-1.87848
H	-1.57383	2.26096	-1.84291
H	-3.92340	4.80635	-0.66916
H	-5.05621	3.63089	-1.35890
H	-4.94218	3.84750	0.41230

```
C          -7.29209        -0.17821         0.01689
C          -7.79311        -1.62236        -0.00937
C          -7.61273         0.42775         1.39226
C          -8.01738         0.61356        -1.08251
H          -7.62766        -2.09968        -0.98734
H          -7.31316        -2.23897         0.76595
H          -8.87579        -1.63950         0.18179
H          -7.31511         1.48477         1.45773
H          -8.69532         0.37547         1.58345
H          -7.09839        -0.12031         2.19629
H          -9.10593         0.55312        -0.93212
H          -7.74169         1.67836        -1.07733
H          -7.78869         0.20811        -2.07979
Cl         -1.01726        -1.14544         2.87670
```

6.6.4 $[Mes*_2P_4Cl]^+$ (cation of salt 8)

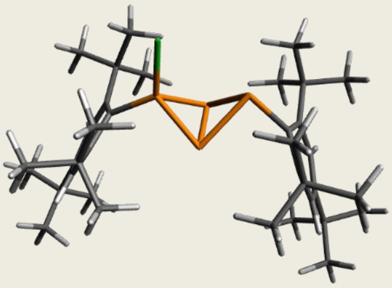

```
99
cation 8 @ PBE-D3/def2-SVP
P          -1.34806        -1.59268         0.22052
P           0.21961        -0.44395         1.18616
P           0.22056        -0.77968        -1.04068
P           1.58867        -1.98849         0.28097
Cl         -1.29831        -3.60725         0.52613
C          -2.88602        -0.69298         0.08894
H          -3.20386        -2.56091         2.31145
H          -3.20265        -3.13727        -1.49237
C          -3.31969         0.03840         1.24051
C          -3.32262        -0.33383        -1.22135
C           2.98010        -0.76862         0.10227
C          -2.69435        -1.81683         2.94059
C          -2.69504        -2.60439        -2.30935
C          -3.97206         1.24367         1.00335
C          -3.23757        -0.40582         2.71644
C          -3.97743         0.89267        -1.35235
C          -3.24227        -1.18971        -2.50218
H           3.42163        -2.66434         2.18398
H           3.44836        -3.19549        -1.32474
C           3.39820        -0.01919         1.23941
C           3.41503        -0.39045        -1.19545
H          -1.60914        -1.90350         2.79246
H          -2.87399        -2.10348         3.98718
H          -1.60950        -2.64428        -2.14409
H          -2.87451        -3.18058        -3.22882
H          -4.28160         1.83592         1.86463
C          -4.26084         1.72839        -0.27509
C          -2.40681         0.58948         3.53803
C          -4.68485        -0.42549         3.25147
H          -4.28450         1.20252        -2.34919
C          -2.41925        -0.47464        -3.58266
C          -4.69212        -1.36866        -3.00108
C           2.95425        -1.96949         2.90012
C           2.98309        -2.74109        -2.21419
C           3.96907         1.23217         1.00815
C           3.43669        -0.52938         2.69952
C           3.99056         0.87785        -1.34128
C           3.46144        -1.30405        -2.44205
C          -4.91432         3.09666        -0.43838
```

H	-2.40269	0.29051	4.59709
H	-2.81033	1.61047	3.48144
H	-1.36198	0.62574	3.19344
H	-5.30872	-1.12402	2.67386
H	-5.16409	0.56198	3.21910
H	-4.68169	-0.75829	4.30025
H	-2.41111	-1.07373	-4.50566
H	-2.83250	0.51334	-3.83028
H	-1.37537	-0.32799	-3.26571
H	-5.31211	-1.86578	-2.23997
H	-5.17286	-0.41633	-3.26063
H	-4.69240	-1.99652	-3.90466
H	1.86237	-2.08441	2.84697
H	3.24635	-2.30871	3.90544
H	3.27825	-3.35790	-3.07632
H	4.25740	1.83542	1.87043
C	4.20993	1.74035	-0.27077
C	2.65869	0.39510	3.64499
C	4.92422	-0.52765	3.11077
H	4.28877	1.19385	-2.33972
C	2.68429	-0.69943	-3.61875
C	4.95089	-1.41628	-2.83135
C	-5.14246	3.45500	-1.90700
C	-3.99340	4.15425	0.18981
C	-6.27108	3.09211	0.28332
C	4.75683	3.15584	-0.44076
H	3.02770	1.42988	3.60513
H	2.76082	0.04634	4.68388
H	1.58476	0.41670	3.40358
H	5.51298	-1.18399	2.45240
H	5.02600	-0.89621	4.14294
H	5.36920	0.47565	3.06934
H	3.04682	0.30526	-3.87881
H	2.79475	-1.33435	-4.51099
H	1.60877	-0.61701	-3.39835
H	5.05792	-2.07196	-3.70899
H	5.39171	-0.44337	-3.08649
H	-5.83479	2.75548	-2.39991
H	-4.20071	3.47485	-2.47661
H	-5.59006	4.45698	-1.97596
H	-4.44332	5.15334	0.08734
H	-3.01144	4.17019	-0.30718
H	-3.82761	3.97331	1.26220
H	-6.76027	4.07138	0.16924
H	-6.16737	2.89847	1.36116
H	-6.94064	2.32611	-0.13633
C	4.95324	3.52273	-1.91203
C	6.11026	3.26925	0.27681
C	3.75695	4.14272	0.18064
H	5.31671	4.55779	-1.99046
H	5.69869	2.87764	-2.40157
H	6.02456	3.07070	1.35541
H	6.51792	4.28509	0.15945
H	6.83975	2.55886	-0.14086
H	3.60529	3.95202	1.25354
H	2.77687	4.07706	-0.31649
H	4.12502	5.17480	0.07440
H	5.53935	-1.84696	-2.00727
H	4.01207	3.45920	-2.47961
H	1.89115	-2.83616	-2.13294

6.6.5 [Mes*$_2$NP$_3$Cl]$^+$ (cation of salt 11)

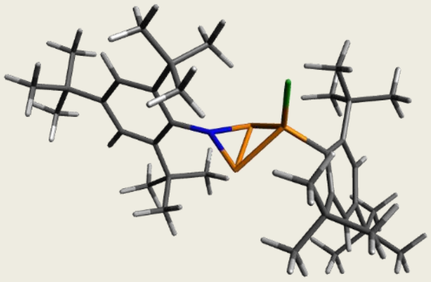

```
99
cation 11 @ PBE0-D3/def2-SVP
P          -1.13077        -1.48924         0.17099
P          -0.09820         0.27308         1.14619
P          -0.01653         0.05342        -1.04852
Cl         -0.77647        -3.49785         0.41483
C          -2.83425        -0.91954         0.03952
H          -2.74888        -2.86826         2.20980
H          -2.57461        -3.29764        -1.63784
C          -3.40829        -0.30695         1.19882
C          -3.30274        -0.58389        -1.26656
C           2.66463         0.22121         0.08731
C          -2.44550        -2.04297         2.86861
C          -2.21037        -2.62931        -2.43022
C          -4.25382         0.77904         0.98713
C          -3.30360        -0.79783         2.65944
C          -4.15679         0.51704        -1.37541
C          -3.07538        -1.37920        -2.57036
H           2.80742        -2.58166        -2.01319
H           1.30807         2.59339        -1.67417
C           3.54170        -0.89634         0.03457
C           3.16757         1.54916         0.06233
H          -1.36785        -1.86122         2.74677
H          -2.57313        -2.39331         3.90343
H          -1.14622        -2.41134        -2.25890
H          -2.24995        -3.19548        -3.37241
H          -4.68114         1.27343         1.85992
C          -4.59385         1.25769        -0.28042
C          -2.79502         0.31414         3.58699
C          -4.73637        -1.19084         3.07677
H          -4.50021         0.80029        -2.36846
C          -2.48199        -0.49102        -3.67231
C          -4.46999        -1.87207        -3.01143
C           2.22526        -2.72714        -1.09104
C           1.08215         2.91054        -0.64471
C           4.90493        -0.64332        -0.09609
C           3.07106        -2.35620         0.13504
C           4.55251         1.70660        -0.06948
C           2.32471         2.83150         0.25949
C          -5.45249         2.51108        -0.41716
H          -2.78885        -0.04197         4.62795
H          -3.42616         1.21298         3.54994
H          -1.76783         0.61887         3.33421
H          -5.13237        -1.98305         2.42388
H          -5.43255        -0.34197         3.03795
H          -4.72911        -1.57076         4.10965
H          -2.39680        -1.06460        -4.60728
H          -3.10352         0.39060        -3.88159
H          -1.47437        -0.13312        -3.41060
H          -4.92704        -2.50385        -2.23518
H          -5.15943        -1.04383        -3.22334
H          -4.37523        -2.47226        -3.92904
H           1.30838        -2.13007        -1.17801
H           1.91755        -3.78242        -1.04456
H           0.72511         3.94985        -0.69750
H           5.58803        -1.48741        -0.14821
C           5.44102         0.64200        -0.16431
```

C	2.32248	-2.56104	1.46022
C	4.24569	-3.34295	0.15495
H	4.95817	2.71146	-0.10114
C	1.92801	2.93446	1.74031
C	3.13726	4.09756	-0.06187
C	-5.70811	2.87606	-1.87937
C	-4.71576	3.67727	0.26039
C	-6.80358	2.27624	0.27563
C	6.94798	0.83262	-0.32829
H	2.98057	-2.31290	2.30632
H	2.00902	-3.60972	1.56934
H	1.42216	-1.94025	1.54403
H	4.83269	-3.31484	-0.77463
H	3.85105	-4.36412	0.25968
H	4.92293	-3.16334	1.00257
H	2.82334	2.98072	2.37730
H	1.33005	3.84197	1.91963
H	1.33319	2.07503	2.08601
H	2.49178	4.97795	0.06874
H	3.99420	4.23140	0.61181
H	-6.27392	2.09331	-2.40724
H	-4.77099	3.05913	-2.42724
H	-6.30429	3.79856	-1.93063
H	-5.31285	4.59831	0.18044
H	-3.74123	3.86085	-0.21768
H	-4.53926	3.48772	1.32966
H	-7.43528	3.17238	0.18090
H	-6.68895	2.06554	1.34917
H	-7.34133	1.43134	-0.18053
C	7.34328	2.30940	-0.34718
C	7.38629	0.19140	-1.65354
C	7.67169	0.14896	0.84147
H	8.43301	2.39889	-0.46631
H	6.87686	2.85043	-1.18474
H	7.16608	-0.88616	-1.68034
H	8.47137	0.31319	-1.79480
H	6.87602	0.66213	-2.50769
H	7.47116	-0.93216	0.87622
H	7.36219	0.58172	1.80502
H	8.76020	0.27999	0.74283
H	3.50307	4.10065	-1.09951
H	7.07253	2.81932	0.59015
H	0.21413	2.34269	-0.26977
N	1.25338	-0.00601	0.11737

6.6.6 [Mes*N(μ-P(C_6F_5))$_2$PMes*] (12)

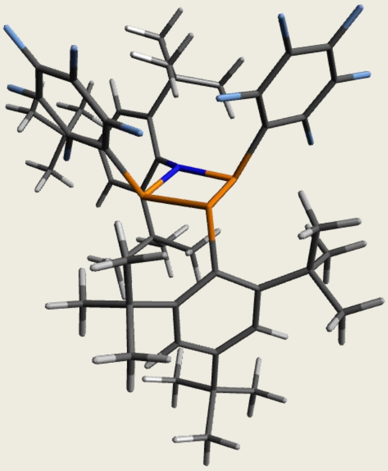

```
120
compound 12 @ PBE0-D3/def2-SVP
C          -0.49336          4.97812          -2.19602
C          -2.49209          1.55714          -2.68131
C           3.87637          2.31345          -1.99448
C          -0.02782          3.67264          -2.07291
C          -1.13761          5.57467          -1.11515
C          -4.72525          0.78823          -2.01565
C           0.87872         -3.30854          -3.22372
C          -4.94483         -3.94010          -3.42807
C           6.56940         -1.97359          -1.90343
C          -3.24698          0.75707          -1.60854
C          -3.40363         -5.53634          -2.30681
C          -0.18242          2.92064          -0.90122
C           4.06635          2.18922          -0.47697
C          -3.50384         -1.71870          -1.92267
C           5.51932          2.57539          -0.12391
C          -2.65880         -0.66206          -1.60745
C           1.75781         -1.58423          -1.74284
C          -3.06522         -3.04032          -2.03314
C          -4.07970         -4.17105          -2.18178
C          -1.31560          4.85890           0.06605
C           3.19606          3.23260           0.22564
C          -1.69779         -3.24239          -1.98328
C           0.70484         -2.68412          -1.82290
C          -1.27371         -0.94233          -1.36793
C          -0.76826         -2.22597          -1.70182
C           4.80885         -0.18137          -0.49993
C          -0.82766          3.55835           0.15974
C           5.17511         -3.79213          -0.94595
C          -3.17285          1.40046          -0.22215
C           5.92241         -2.49179          -0.61845
C           3.87571          0.73111           0.00937
C           4.94472         -1.47941          -0.02274
C           1.02559         -3.74193          -0.75648
C          -4.96604         -4.17812          -0.92604
C           7.02926         -2.78216           0.40603
C           2.95755          0.26958           0.99151
C           4.20228         -1.79996           1.11296
C           3.24883         -0.94891           1.67372
C          -1.50439         -0.46805           2.05049
C          -2.48981         -1.44360           1.85096
C          -3.72645         -1.40068           2.48582
C          -1.77955          0.51027           3.00842
C           2.75312         -1.36987           3.07987
C           2.08842         -2.75252           3.06426
C          -3.98335         -0.38481           3.40330
C          -3.00357          0.56766           3.67275
C           1.81084         -0.38476           3.77009
C           4.01215         -1.42781           3.97109
H          -2.75196          1.17667          -3.68078
H           4.08638          3.34535          -2.31824
H          -2.75619          2.62606          -2.63968
H           4.55663          1.64761          -2.54528
H           0.65074         -2.57196          -4.00947
H          -4.89582          0.35705          -3.01322
H           2.84839          2.06222          -2.29168
```

H	-4.32556	-3.92248	-4.33813
H	-1.40342	1.47018	-2.58999
H	5.81400	-1.72104	-2.66349
H	-5.05994	1.83644	-2.04705
H	-2.75802	-5.59310	-3.19680
H	1.92325	-3.63287	-3.35299
H	7.22762	-2.74647	-2.32853
H	1.54812	-0.75561	-2.43294
H	0.23854	-4.18530	-3.38880
H	-5.68836	-4.74602	-3.53474
H	5.70681	3.62060	-0.41573
H	7.18671	-1.08053	-1.72279
H	-5.49346	-2.98798	-3.37695
H	6.25936	1.94807	-0.63869
H	-4.56392	-1.51432	-2.05506
H	5.46059	0.15345	-1.30550
H	3.55138	4.23772	-0.05017
H	-4.16723	-6.32351	-2.40007
H	-5.36710	0.26258	-1.29265
H	2.73504	-2.00360	-2.02117
H	4.39514	-3.61863	-1.70279
H	2.13843	3.19740	-0.06570
H	-3.50484	2.44995	-0.26183
H	5.87302	-4.54604	-1.34290
H	-1.31237	-4.24695	-2.14492
H	-2.79219	-5.77008	-1.42196
H	1.88792	-1.18429	-0.72999
H	5.69561	2.48372	0.95876
H	-2.16668	1.36131	0.19350
H	3.25529	3.14928	1.32113
H	-3.83874	0.86672	0.47344
H	7.75292	-3.50619	-0.00157
H	4.68812	-4.22076	-0.05771
H	2.06076	-4.09737	-0.87657
H	0.35817	-4.61331	-0.82742
H	-5.69842	-5.00000	-0.97296
H	7.57341	-1.86121	0.66570
H	-5.52274	-3.23541	-0.81770
H	-4.35759	-4.30318	-0.01783
H	0.92585	-3.31597	0.25154
H	6.62114	-3.20390	1.33672
H	4.38611	-2.75761	1.60291
H	1.17949	-2.74360	2.44562
H	2.75806	-3.52877	2.66711
H	0.79348	-0.39047	3.35995
H	2.18891	0.64787	3.74550
H	4.74473	-2.16362	3.61256
H	4.51074	-0.44697	4.00342
H	1.80734	-3.04755	4.08746
H	1.70495	-0.67037	4.82809
H	3.73082	-1.70499	4.99932
F	-0.33100	5.65211	-3.32098
F	0.55465	3.14301	-3.13998
F	-1.58574	6.80945	-1.21307
F	-1.93460	5.42145	1.08879
F	-1.02499	2.93896	1.31050
F	-2.27029	-2.45793	1.02688
F	-4.64191	-2.32462	2.24526
F	-0.87730	1.41346	3.35072
F	-5.14271	-0.33536	4.02847

```
F          -3.23696         1.51112         4.56839
N          -0.49411        -0.03857        -0.55898
P           0.65295         1.23435        -0.93773
P           1.31231         1.13211         1.22529
P           0.01864        -0.69982         0.98964
```

6.6.7 [$(F_5C_6)P(\mu\text{-}PMes^*)]_2$ (13)

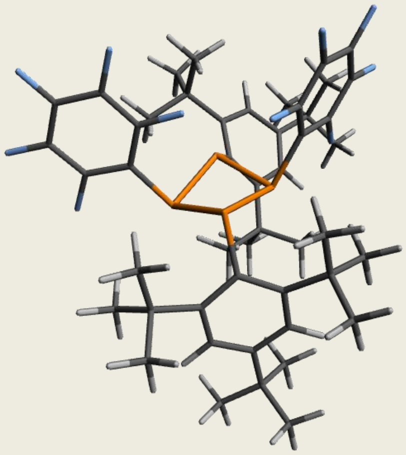

```
120
compound 13 @ PBE0-D3/def2-SVP
C           5.96582        -2.86728        -3.39152
C          -1.04525         5.39154        -1.63441
C          -5.77256        -3.17599        -2.39913
C           0.11592        -3.09707        -3.52274
C          -4.67809         1.66991        -1.61197
C           4.41615         1.80844        -3.30538
C          -1.31684         4.02571        -1.63528
C           2.07182         2.35492        -2.83498
C           4.73013        -4.55718        -2.04754
C          -3.92713        -4.66090        -1.65567
C          -0.27579         5.93238        -0.60790
C           5.11648        -3.08044        -2.13160
C           4.05723        -0.78253        -2.25811
C           3.29528         1.61972        -2.26826
C          -1.10584        -1.59733        -2.02340
C           3.89354        -2.16767        -2.15979
C          -5.01124        -3.68726        -1.17523
C           2.59084        -2.64166        -2.08585
C           0.11236        -2.51120        -2.09407
C           3.00536         0.11032        -2.08222
C           1.47528        -1.80675        -1.92868
C          -0.84837         3.16924        -0.63308
C          -4.63317        -1.20619        -0.63848
C          -4.66734         1.23140        -0.14230
C          -6.12602         1.06684         0.33796
C           1.71393        -0.41791        -1.75272
C          -4.36691        -2.54724        -0.38630
C          -5.99560        -4.43206        -0.26064
C           0.20804         5.10921         0.40739
C           5.93630        -2.72001        -0.88166
C          -4.01185        -0.15724         0.05213
C          -4.08513         2.35767         0.71407
C           3.76371         2.27940        -0.95982
C          -0.07919         3.74758         0.38332
C          -0.06433        -3.65481        -1.08600
C          -3.56483        -2.81756         0.72331
C          -2.98515        -0.48394         0.97806
C          -2.91851        -1.82449         1.46058
C          -2.38086        -2.26699         2.84455
C           1.42927        -0.43993         2.24327
C          -1.31191        -3.36209         2.74595
C          -3.60031        -2.83906         3.60016
C           2.53611        -1.28471         2.09022
C          -1.86485        -1.12701         3.72385
C           1.50419         0.51666         3.25803
C           3.67275        -1.17951         2.88379
C           2.62588         0.63173         4.07790
C           3.71885        -0.20885         3.88174
```

H	5.39247	-3.11317	-4.29857
H	0.22864	-2.29829	-4.27183
H	4.20200	1.26522	-4.23823
H	-5.11843	-2.59820	-3.07019
H	-6.17440	-4.02637	-2.97083
H	-5.20399	0.94616	-2.25143
H	6.85872	-3.51187	-3.36538
H	1.72742	1.88759	-3.76986
H	-5.19657	2.63651	-1.71240
H	4.14691	-4.87929	-2.92401
H	4.50819	2.87848	-3.54544
H	-0.83796	-3.61416	-3.71336
H	6.31109	-1.82706	-3.48439
H	-3.65799	1.78609	-2.00176
H	0.92838	-3.82060	-3.67747
H	2.33196	3.40370	-3.04766
H	-3.22090	-4.16041	-2.33541
H	-6.62454	-2.53856	-2.11842
H	-4.38218	-5.50388	-2.19940
H	-0.98797	-0.70515	-2.65288
H	5.63830	-5.17757	-2.00779
H	5.39786	1.48194	-2.93390
H	-5.37232	-0.94820	-1.39551
H	-1.99291	-2.14252	-2.37698
H	5.05547	-0.38694	-2.44129
H	1.22229	2.38043	-2.13792
H	-6.67721	0.32304	-0.25343
H	2.41875	-3.71559	-2.13500
H	-6.65740	2.02794	0.25203
H	4.14128	-4.77155	-1.14279
H	-6.48558	-5.25386	-0.80721
H	-3.34876	-5.07701	-0.81781
H	-4.75169	3.23206	0.65449
H	-1.34795	-1.28515	-0.99845
H	6.81057	-3.38393	-0.78869
H	-3.09900	2.70300	0.37729
H	4.06251	3.32273	-1.15158
H	6.30353	-1.68375	-0.92618
H	-1.02168	-4.16568	-1.26635
H	-6.77728	-3.75148	0.11015
H	0.73475	-4.40635	-1.16317
H	-6.15647	0.74855	1.39129
H	4.63580	1.74652	-0.55049
H	-5.48763	-4.86600	0.61367
H	5.32720	-2.81712	0.02994
H	2.97387	2.27945	-0.19678
H	-4.00916	2.07222	1.77398
H	-0.07483	-3.26771	-0.05855
H	-3.46214	-3.85227	1.05603
H	-1.67042	-4.23099	2.17498
H	-4.02150	-3.72491	3.10548
H	-0.40254	-2.99197	2.25176
H	-4.39933	-2.08600	3.67735
H	-0.90935	-0.70563	3.39195
H	-2.59316	-0.30549	3.79571
H	-1.03834	-3.71439	3.75284
H	-3.30335	-3.13189	4.61971
H	-1.69191	-1.50683	4.74261
F	-1.50200	6.17169	-2.59829
F	-2.03950	3.55166	-2.63997

```
F          -0.00649         7.22230        -0.59450
F           0.93971         5.62650         1.37796
F           0.41305         3.01404         1.36644
F           2.52975        -2.22292         1.15023
F           0.49791         1.33875         3.50170
F           4.70397        -1.98730         2.69642
F           2.66388         1.54232         5.03484
F           4.78654        -0.09632         4.64758
P          -1.22547         1.36278        -0.81588
P           0.88322         0.66758        -0.52033
P          -1.60884         0.74674         1.34214
P           0.08499        -0.71972         0.99917
```

6.6.8 Mes*PPC$_6$F$_5$

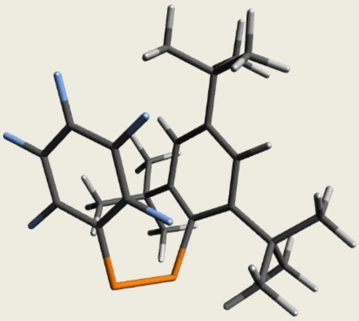

```
60
Mes*PPC6F5 @ PBE0-D3/def2-SVP
C          -0.08436         0.89647         1.63454
C          -0.47066        -0.44022         1.52400
C          -1.36842        -0.78093         0.47345
C          -2.07793         0.24441        -0.21091
C          -1.62976         1.55935        -0.04584
C          -0.59082         1.90619         0.81518
H           0.62583         1.17014         2.41316
H          -2.12217         2.34772        -0.60743
C          -3.38866        -0.00365        -0.99828
C          -3.21040        -0.74998        -2.32730
C          -4.34650        -0.79130        -0.08689
C          -4.09098         1.31863        -1.33458
H          -2.58999        -0.17530        -3.02741
H          -2.74760        -1.73906        -2.20584
H          -4.19785        -0.90667        -2.78919
H          -4.49708        -0.26655         0.86878
H          -5.32583        -0.90446        -0.57779
H          -3.97479        -1.80265         0.13636
H          -5.05733         1.09755        -1.81195
H          -4.29229         1.92060        -0.43588
H          -3.51187         1.93013        -2.04259
C          -0.11854         3.35061         0.98940
C           1.41355         3.41072         1.01917
C          -0.60848         4.24982        -0.14757
C          -0.67392         3.88266         2.31971
H           1.83919         2.80603         1.83319
H           1.84455         3.05716         0.07312
H           1.74845         4.44847         1.17219
H          -1.70420         4.35090        -0.15294
H          -0.19011         5.26067        -0.02889
H          -0.29021         3.86365        -1.12741
H          -0.36167         4.92736         2.47855
H          -1.77412         3.84764         2.32714
H          -0.31311         3.28768         3.17244
C          -0.01736        -1.42446         2.62909
C           0.26978        -0.66086         3.93269
C          -1.13706        -2.41976         2.95541
C           1.25556        -2.19015         2.25824
H           1.17522        -0.04043         3.86731
H          -0.57263        -0.01501         4.22310
H           0.44073        -1.38515         4.74315
```

```
H          -1.34943        -3.11595         2.12572
H          -0.84490        -3.04600         3.81244
H          -2.07534        -1.90155         3.20507
H           1.58168        -2.80974         3.10867
H           1.09957        -2.85910         1.40388
H           2.07608        -1.49968         2.01417
P          -1.54151        -2.51951        -0.12212
P           0.08769        -2.94371        -1.28203
C           1.17728        -1.45608        -1.25996
C           0.82464        -0.23452        -1.83622
C           2.45698        -1.55114        -0.70906
C           1.65924         0.87553        -1.77541
C           3.30556        -0.45179        -0.61868
C           2.90449         0.76566        -1.16234
F           2.89099        -2.70557        -0.22297
F           4.48830        -0.56015        -0.03850
F           3.70059         1.81603        -1.09315
F           1.28769         2.03057        -2.30441
F          -0.31767        -0.10658        -2.48942
```

6.6.9 Mes*NPC$_6$F$_5$

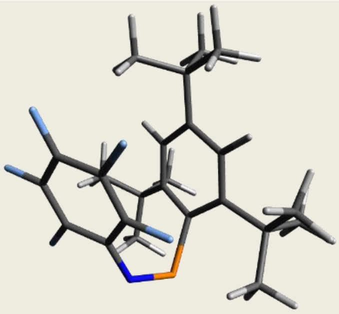

```
60
Mes*NPC6F5 @ PBE0-D3/def2-SVP
C           0.08650        -0.46365        -1.78571
C          -0.29843         0.83122        -1.43422
C          -1.29323         0.99174        -0.42865
C          -2.00280        -0.14958         0.03670
C          -1.55162        -1.41454        -0.35586
C          -0.47375        -1.60455        -1.21513
H           0.85945        -0.58688        -2.54437
H          -2.06229        -2.28905         0.03627
C          -3.33022        -0.10185         0.82175
C          -3.55624        -1.37292         1.65493
C          -3.48110         1.08098         1.77891
C          -4.43978        -0.00219        -0.24102
H          -3.71252        -2.26584         1.03362
H          -2.71064        -1.56173         2.33133
H          -4.46416        -1.25261         2.26466
H          -3.50551         2.05124         1.25471
H          -4.44846         1.00950         2.30072
H          -2.69062         1.10330         2.54142
H          -5.43299         0.00501         0.23660
H          -4.33396         0.91947        -0.83359
H          -4.39686        -0.85420        -0.93606
C           0.05634        -2.99063        -1.58210
C           1.57390        -3.03585        -1.35535
C          -0.58359        -4.08866        -0.73114
C          -0.25098        -3.26385        -3.06169
H           2.10616        -2.28529        -1.95801
H           1.82564        -2.85733        -0.30057
H           1.96969        -4.02518        -1.63358
H          -1.66747        -4.16859        -0.90516
H          -0.13894        -5.06247        -0.98566
H          -0.41601        -3.91219         0.34238
H           0.12010        -4.25872        -3.35522
H          -1.33516        -3.23260        -3.25003
H           0.22578        -2.52054        -3.71838
```

```
C          0.27985        1.96301       -2.30892
C         -0.45091        1.86285       -3.66141
C          0.05294        3.37394       -1.77149
C          1.78969        1.79995       -2.54098
H         -0.29627        0.88006       -4.13060
H         -1.53497        2.00438       -3.53228
H         -0.08127        2.63600       -4.35406
H          0.62009        3.56844       -0.85055
H          0.39224        4.11026       -2.51703
H         -1.01599        3.59014       -1.60068
H          2.16155        2.63752       -3.15087
H          2.34535        1.80475       -1.59447
H          2.03890        0.87675       -3.08165
P         -1.31463        2.55352        0.59805
C          0.80883        1.29549        1.57980
C          0.49764        0.02745        2.09035
C          2.10785        1.47187        1.08136
C          1.38616       -1.03698        1.99750
C          3.00489        0.41496        0.97864
C          2.65168       -0.84610        1.45129
F          2.46621        2.65727        0.59976
F          4.19172        0.60705        0.42554
F          3.49212       -1.86371        1.34394
F          1.02595       -2.24078        2.41774
F         -0.68889       -0.18730        2.64836
N         -0.08389        2.34688        1.57385
```

6.6.10 Mes*$_2$P$_5$N(Mes*)PNMes* (16)

```
196
compound 16 @ PBE-D3/def2-SVP
P          3.46746        0.07519       -0.39547
P          2.17230        0.23939        1.45528
P          1.49998       -0.89775       -1.06364
C          3.31332        1.81823       -1.05322
P          1.59518       -1.89836        0.98165
C          3.31051       -2.64632        0.95700
P          0.63273        0.92775       -0.09438
N         -1.03329        0.50747        0.29531
P         -1.49884       -1.15485        0.19632
N         -3.04381       -1.26923        0.36160
C          3.40357        2.91517       -0.14824
C          2.64086        4.04771       -0.42542
C          4.39686        3.02490        1.03220
H          2.63595        4.85747        0.30682
C          1.89164        4.19396       -1.59555
C          2.09855        3.23756       -2.58538
C          0.93462        5.37292       -1.75201
H          1.66041        3.38893       -3.57171
C          2.83197        2.06481       -2.36326
C          3.13847        1.19543       -3.60157
C          5.34055        1.82915        1.17823
H          6.12556        2.07585        1.91033
H          5.83757        1.58873        0.22676
H          4.84667        0.91999        1.54649
C          3.69406        3.28113        2.37014
H          4.44033        3.46472        3.15930
H          3.08724        2.41491        2.67009
```

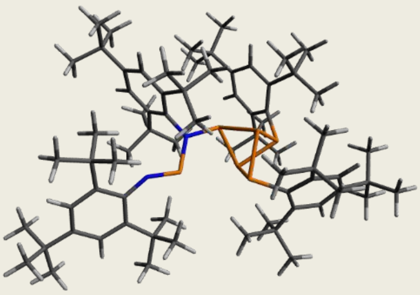

H	3.03235	4.15857	2.32430
C	5.29952	4.23398	0.70787
H	6.06471	4.35036	1.49193
H	4.73234	5.17335	0.64917
H	5.81277	4.09107	-0.25539
C	-0.18469	5.21104	-0.71108
H	-0.72446	4.26179	-0.84490
H	-0.91294	6.03387	-0.79393
H	0.21725	5.21494	0.31328
C	1.67367	6.69667	-1.51338
H	2.09085	6.75969	-0.49768
H	0.98372	7.54624	-1.63849
H	2.50285	6.82008	-2.22709
C	0.31076	5.41824	-3.14651
H	1.07166	5.56394	-3.92895
H	-0.40001	6.25605	-3.21173
H	-0.24230	4.49571	-3.37511
C	3.82288	2.12106	-4.62727
H	4.73826	2.56384	-4.20576
H	3.17036	2.94382	-4.95090
H	4.10064	1.54260	-5.52256
C	1.86408	0.62028	-4.23094
H	2.11232	0.06084	-5.14696
H	1.15314	1.41361	-4.50445
H	1.35248	-0.07013	-3.54347
C	4.13115	0.06096	-3.33572
H	5.03976	0.42722	-2.83388
H	4.43439	-0.38792	-4.29443
H	3.70971	-0.75353	-2.73199
C	3.78903	-3.29561	-0.20911
C	5.16301	-3.24235	-0.47652
C	2.95433	-4.17575	-1.16559
H	5.51905	-3.67563	-1.41051
C	6.07726	-2.65125	0.38742
C	5.59473	-2.28100	1.64486
C	7.54725	-2.43815	0.03312
H	6.30775	-1.95388	2.40432
C	4.24233	-2.31014	1.98083
C	3.91092	-2.11542	3.47943
C	1.48594	-4.33568	-0.77225
H	1.37185	-4.63558	0.28022
H	1.03548	-5.12636	-1.39266
H	0.88638	-3.43026	-0.94475
C	3.02657	-3.68524	-2.61737
H	4.06376	-3.59095	-2.97045
H	2.53592	-2.70736	-2.73005
H	2.50972	-4.39612	-3.28149
C	3.56899	-5.58877	-1.08624
H	4.61688	-5.60708	-1.41609
H	3.00105	-6.28025	-1.72877
H	3.53493	-5.97080	-0.05450
C	7.82820	-0.92827	0.05742
H	8.87968	-0.72450	-0.20163
H	7.63191	-0.49745	1.05040
H	7.18316	-0.40343	-0.66365
C	7.88330	-2.97078	-1.36011
H	8.94278	-2.77797	-1.58776
H	7.28062	-2.47919	-2.13903
H	7.72055	-4.05710	-1.43326
C	8.44495	-3.14869	1.05519

H	9.50690	-3.00178	0.80162
H	8.24239	-4.23063	1.07052
H	8.29200	-2.76372	2.07416
C	4.63685	-3.25952	4.21727
H	5.72620	-3.21750	4.07804
H	4.29015	-4.23874	3.85303
H	4.43043	-3.20054	5.29779
C	4.42249	-0.77294	4.01733
H	5.49921	-0.63853	3.83920
H	4.25524	-0.71535	5.10445
H	3.88950	0.06721	3.54849
C	2.43000	-2.25417	3.84029
H	2.33154	-2.29811	4.93621
H	1.99156	-3.17745	3.43324
H	1.82495	-1.39812	3.50947
C	-1.90755	1.63719	0.44064
C	-2.02610	2.25761	1.71260
C	-2.62871	2.13756	-0.68307
C	-2.89360	3.34994	1.82973
H	-2.98302	3.83724	2.79473
C	-3.65766	3.83131	0.77627
C	-3.50171	3.20410	-0.45488
H	-4.09481	3.57088	-1.28897
C	-1.19639	1.92046	2.97676
C	-0.84229	0.44035	3.17822
C	0.08688	2.76396	2.90623
C	-1.95136	2.32005	4.25866
H	-0.12867	0.05276	2.44685
H	-1.73889	-0.19213	3.14809
H	-0.37983	0.31686	4.16921
H	0.73985	2.55547	3.76868
H	-0.15743	3.83760	2.90423
H	0.66138	2.56241	1.99075
H	-1.37728	1.97939	5.13273
H	-2.94562	1.84989	4.30225
H	-2.07463	3.40587	4.37116
C	-4.63409	4.99931	0.91904
C	-4.68905	5.53060	2.35166
C	-6.04174	4.53074	0.52300
C	-4.19012	6.14120	-0.00641
H	-5.01681	4.75575	3.06152
H	-5.40662	6.36294	2.41315
H	-3.71153	5.91136	2.68512
H	-6.07509	4.17071	-0.51589
H	-6.76295	5.35857	0.61412
H	-6.37757	3.70729	1.17122
H	-3.18370	6.49471	0.26564
H	-4.88556	6.99256	0.06842
H	-4.15881	5.82333	-1.05917
C	-2.62060	1.60621	-2.14284
C	-3.78291	0.61764	-2.32130
C	-2.84665	2.77143	-3.12966
C	-1.31516	0.95346	-2.60783
H	-3.83458	0.27968	-3.36893
H	-4.74247	1.09228	-2.06622
H	-3.67396	-0.26282	-1.67889
H	-3.87003	3.16961	-3.10433
H	-2.67933	2.41170	-4.15561
H	-2.15148	3.60278	-2.94212
H	-0.45083	1.61519	-2.44160

H	-1.37965	0.75934	-3.68890
H	-1.10393	-0.01214	-2.13530
C	-4.15857	-2.06593	0.17780
C	-4.19096	-3.18784	-0.71600
C	-5.37549	-1.70481	0.85230
C	-5.43233	-3.74556	-1.04199
H	-5.45547	-4.55526	-1.76405
C	-6.63378	-3.33603	-0.47859
C	-6.55767	-2.34813	0.50262
H	-7.47524	-2.05499	1.00867
C	-2.94090	-3.87384	-1.30148
C	-2.22286	-2.99448	-2.33744
C	-2.04065	-4.32479	-0.13873
C	-3.28507	-5.16897	-2.05446
H	-1.29459	-3.47916	-2.67852
H	-1.94383	-1.99933	-1.97351
H	-2.87556	-2.84518	-3.21063
H	-1.15046	-4.84194	-0.52368
H	-2.59629	-5.03053	0.49729
H	-1.68856	-3.51445	0.51115
H	-2.34726	-5.63793	-2.38903
H	-3.89397	-4.98695	-2.95237
H	-3.81082	-5.89513	-1.41686
C	-7.98523	-3.93856	-0.85999
C	-7.85415	-5.00254	-1.95004
C	-8.62115	-4.58843	0.37752
C	-8.90153	-2.82142	-1.38083
H	-7.41179	-4.59101	-2.87012
H	-8.84850	-5.39866	-2.20673
H	-7.23464	-5.85067	-1.62055
H	-7.97929	-5.39187	0.77029
H	-9.60148	-5.02384	0.12489
H	-8.77770	-3.85902	1.18591
H	-9.06288	-2.04098	-0.62245
H	-9.88709	-3.22818	-1.65962
H	-8.46458	-2.33924	-2.26879
C	-5.43268	-0.64453	1.96224
C	-6.79627	-0.59866	2.66296
C	-5.19142	0.74186	1.36182
C	-4.40091	-0.97550	3.04764
H	-7.07365	-1.56808	3.10496
H	-7.60490	-0.27771	1.98906
H	-6.74659	0.13767	3.47960
H	-5.20762	1.51068	2.14990
H	-5.97984	0.98260	0.63190
H	-4.22576	0.79402	0.85308
H	-3.39145	-1.04126	2.63104
H	-4.63748	-1.94002	3.52354
H	-4.40501	-0.19905	3.82928

6.6.11 Mes*$_2$NP$_3$ (17, 18)

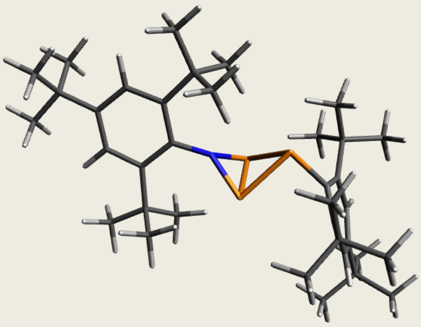

```
98
Mes*2NP3 bicyclic isomer (18) @ PBE-D3/def2-SVP
P           1.04007          1.51650          0.74659
P           0.22573         -0.60147          1.04449
P           0.08417          0.27926         -0.91970
C           2.78805          0.95213          0.36279
H           2.67142          2.19210          2.96447
H           2.42483          3.73422         -0.33748
C           3.47052          0.07399          1.26868
C           3.30440          1.11640         -0.96183
C          -2.62962         -0.15030          0.15567
C           2.44277          1.19611          3.39440
C           2.08622          3.40023         -1.33879
C           4.42325         -0.81316          0.73725
C           3.36136          0.10083          2.82127
C           4.26738          0.19339         -1.42174
C           3.00197          2.30651         -1.91872
H          -2.68114          3.06481         -1.46529
H          -1.57328         -2.52142         -2.05211
C          -3.49878          1.00008          0.22961
C          -3.20250         -1.44826         -0.08139
H           1.36842          0.98695          3.23528
H           2.59929          1.26345          4.49063
H           1.02876          3.08524         -1.26246
H           2.10903          4.28363         -2.00940
H           4.90839         -1.52629          1.41982
C           4.79043         -0.82841         -0.61864
C           2.91112         -1.26816          3.37540
C           4.78384          0.41875          3.35744
H           4.61922          0.28783         -2.45687
C           2.40360          1.81386         -3.25460
C           4.36224          3.00065         -2.20118
C          -2.11921          3.00946         -0.51081
C          -1.24110         -2.82012         -1.03754
C          -4.86654          0.82678         -0.03277
C          -3.01036          2.42448          0.60654
C          -4.59142         -1.52518         -0.31768
C          -2.42717         -2.79471         -0.04738
C           5.76228         -1.89745         -1.14396
H           2.89327         -1.24757          4.48453
H           3.59322         -2.08401          3.06462
H           1.89340         -1.52906          3.02177
H           5.13880          1.39637          2.97330
H           5.52807         -0.34724          3.06644
H           4.76663          0.46882          4.46570
H           2.25217          2.66805         -3.94623
H           3.06666          1.08438         -3.76027
H           1.42181          1.32292         -3.09959
H           4.81936          3.36433         -1.25884
H           5.08979          2.32502         -2.69019
H           4.20849          3.87233         -2.87022
H          -1.21027          2.40677         -0.68039
H          -1.78978          4.03526         -0.24434
H          -0.83003         -3.84862         -1.10489
H          -5.52217          1.70407          0.00070
C          -5.44313         -0.41665         -0.32830
C          -2.27827          2.39715          1.96791
```

```
C          -4.18454        3.41588        0.77583
H          -5.02730       -2.50749       -0.51448
C          -1.97496       -3.07968        1.40366
C          -3.31757       -3.99403       -0.45257
C           6.03360       -1.74382       -2.65024
C           5.14266       -3.29225       -0.89843
C           7.10759       -1.78936       -0.39069
C          -6.94785       -0.51762       -0.62915
H          -2.96656        2.05113        2.76586
H          -1.92899        3.41652        2.23284
H          -1.39935        1.73156        1.94796
H          -4.74078        3.58465       -0.16815
H          -3.77799        4.39676        1.09412
H          -4.90394        3.08595        1.55209
H          -2.85790       -3.18148        2.06634
H          -1.39054       -4.02217        1.45407
H          -1.34104       -2.27421        1.82112
H          -2.70781       -4.91847       -0.40966
H          -4.17607       -4.14007        0.23176
H           6.51689       -0.77451       -2.88734
H           5.10071       -1.81906       -3.24453
H           6.71580       -2.54730       -2.99360
H           5.82304       -4.08963       -1.26296
H           4.17444       -3.39308       -1.42902
H           4.95578       -3.47357        0.17869
H           7.81829       -2.56072       -0.75346
H           6.98308       -1.93598        0.70072
H           7.57013       -0.79363       -0.54595
C          -7.38757       -1.96262       -0.92144
C          -7.27387        0.35031       -1.86615
C          -7.74940       -0.00094        0.58720
H          -8.47405       -1.98809       -1.14076
H          -6.85884       -2.38442       -1.79994
H          -7.00680        1.41326       -1.70260
H          -8.35847        0.30598       -2.09800
H          -6.71518       -0.00432       -2.75586
H          -7.50540        1.05409        0.82201
H          -7.53193       -0.60632        1.49034
H          -8.83973       -0.05784        0.38714
H          -3.70527       -3.89744       -1.48649
H          -7.20599       -2.63226       -0.05657
H          -0.39245       -2.17091       -0.75159
N          -1.24087        0.12156        0.30265
```

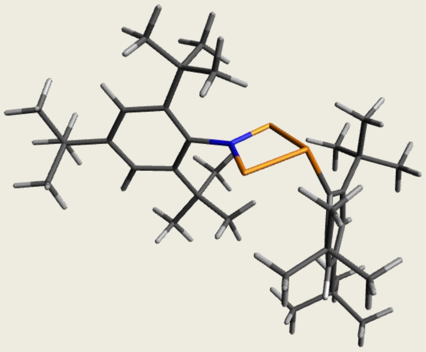

```
98
Mes*2NP3 biradical (17) @ PBE-D3/def2-SVP
P          -1.14625       -2.23249        0.33012
P           0.34071       -1.03321        1.48271
P           0.14383       -1.55223       -1.35441
C          -2.63559       -1.07497        0.25033
H          -2.80242       -2.98536        2.44898
H          -3.08538       -3.55551       -1.20382
C          -3.04953       -0.35817        1.41974
C          -3.21542       -0.73563       -1.01121
C           2.38711       -0.25124       -0.16247
C          -2.32532       -2.22948        3.10392
C          -2.70979       -3.05290       -2.11722
C          -3.73882        0.85335        1.23408
```

C	-2.92281	-0.82473	2.89941
C	-3.89611	0.49659	-1.11489
C	-3.29090	-1.63604	-2.27834
H	0.02096	1.96310	1.06775
H	2.64495	-2.99624	1.82222
C	2.41060	1.17247	-0.32366
C	3.58017	-1.01480	0.00969
H	-1.22803	-2.28037	2.95176
H	-2.49806	-2.54366	4.15370
H	-1.60163	-3.08861	-2.10350
H	-3.01699	-3.66977	-2.98648
H	-4.00000	1.43981	2.12658
C	-4.10613	1.35031	-0.02614
C	-2.11416	0.19199	3.73529
C	-4.36834	-0.90436	3.46317
H	-4.27643	0.79044	-2.10139
C	-2.62517	-0.95072	-3.49201
C	-4.80063	-1.84268	-2.58237
C	-0.12209	1.84055	-0.02344
C	2.64366	-3.34447	0.77145
C	3.65840	1.80156	-0.16825
C	1.20346	2.04075	-0.79772
C	4.78315	-0.29133	0.15008
C	3.66765	-2.56644	-0.08888
C	-4.76444	2.73382	-0.15154
H	-2.05089	-0.14128	4.79177
H	-2.58208	1.19618	3.72725
H	-1.08237	0.29733	3.34322
H	-4.97507	-1.63192	2.88706
H	-4.89049	0.07067	3.42849
H	-4.34171	-1.23683	4.52149
H	-2.71324	-1.59200	-4.39324
H	-3.09936	0.02302	-3.72479
H	-1.54893	-0.76576	-3.30164
H	-5.30912	-2.33855	-1.73090
H	-5.32642	-0.88943	-2.78008
H	-4.91972	-2.48568	-3.47877
H	-0.60435	0.86412	-0.21045
H	-0.84799	2.61117	-0.35375
H	2.91620	-4.41970	0.76768
H	3.71100	2.89192	-0.25810
C	4.84835	1.10609	0.09859
C	0.97635	1.73366	-2.29813
C	1.52361	3.54988	-0.69017
H	5.70922	-0.84915	0.30962
C	3.47773	-2.94355	-1.57903
C	5.06077	-3.08082	0.34060
C	-5.06227	3.10206	-1.61508
C	-3.81054	3.79869	0.43554
C	-6.09408	2.74218	0.63637
C	6.15979	1.88774	0.29248
H	1.89728	1.92192	-2.88566
H	0.16612	2.37666	-2.70126
H	0.67527	0.67854	-2.45740
H	1.75450	3.85161	0.35159
H	0.63631	4.12685	-1.01749
H	2.36530	3.85890	-1.34041
H	4.22565	-2.42909	-2.21544
H	3.60075	-4.03869	-1.71354
H	2.46536	-2.67005	-1.94029

```
H          5.06926       -4.18660        0.27541
H          5.87519       -2.71496       -0.31479
H         -5.78663        2.40222       -2.07858
H         -4.14118        3.10418       -2.23221
H         -5.50475        4.11733       -1.66567
H         -4.26779        4.80796        0.37366
H         -2.85126        3.81939       -0.12031
H         -3.57513        3.59767        1.49958
H         -6.58413        3.73533        0.56281
H         -5.93581        2.52370        1.71128
H         -6.79412        1.98056        0.23726
C          7.35478        0.96055        0.57333
C          5.99990        2.85271        1.48932
C          6.46250        2.69873       -0.98818
H          8.27339        1.56489        0.71480
H          7.20434        0.36139        1.49387
H          5.17322        3.57342        1.32963
H          6.93118        3.43620        1.64382
H          5.78373        2.29375        2.42210
H          5.65465        3.42130       -1.21867
H          6.57481        2.02741       -1.86347
H          7.40444        3.27358       -0.87016
H          5.30140       -2.80405        1.38717
H          7.54340        0.26152       -0.26640
H          1.61255       -3.27873        0.37928
N          1.10023       -0.89589       -0.06366
```

6.6.12 $H_2N_2P_2$

```
6
H2N2P2 bicyclic isomer @ PBE-D3/def2-TZVP
P          1.03801       -0.00000       -0.31033
N          0.00000        1.15012        0.64009
P         -1.03801        0.00000       -0.31033
N         -0.00000       -1.15012        0.64009
H          0.00000       -2.07551        0.17436
H          0.00000        2.07551        0.17436
```

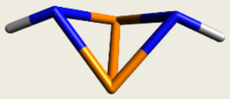

```
6
H2N2P2 biradical @ PBE-D3/def2-TZVP
P          0.00000        1.31765       -0.00000
N          0.00000        0.00000        1.09579
P         -0.00000       -1.31765       -0.00000
N          0.00000        0.00000       -1.09579
H         -0.00000       -0.00000       -2.10903
H         -0.00000       -0.00000        2.10903
```

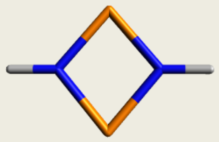

6.6.13 H_2NP_3

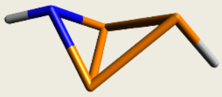

```
6
H2NP3 bicyclic isomer @ PBE-D3/def2-TZVP
P           0.19768        -0.54191         1.06474
P           0.19768         1.42683        -0.00000
P           0.19768        -0.54191        -1.06474
N          -1.28008        -0.71476         0.00000
H          -1.57021        -1.70687         0.00000
H           1.63529         1.56510        -0.00000
```

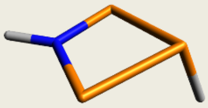

```
6
H2NP3 biradical @ PBE-D3/def2-TZVP
P           0.00040        -0.35134         1.44514
P           0.00040         1.32943        -0.00000
P           0.00040        -0.35134        -1.44514
N           0.14460        -1.25315         0.00000
H           0.37020        -2.25294         0.00000
H          -1.40044         1.62377        -0.00000
```

6.6.14 H_2P_4

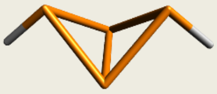

```
6
H2P4 bicyclic isomer @ PBE-D3/def2-TZVP
P           1.09411        -0.00000        -0.65521
P           0.00000         1.43413         0.67424
P          -1.09411         0.00000        -0.65521
P          -0.00000        -1.43413         0.67424
H           0.00000        -2.50997        -0.28535
H           0.00000         2.50997        -0.28535
```

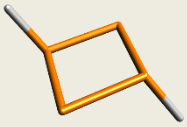

```
6
H2P4 biradical @ PBE-D3/def2-TZVP
P           0.00000         0.00000         1.68005
P           0.00000         1.31022         0.00000
P           0.00000         0.00000        -1.68005
P          -0.00000        -1.31022         0.00000
H           1.08514        -2.24539        -0.00000
H          -1.08514         2.24539        -0.00000
```

6.6.15 P_5H_3 (19)

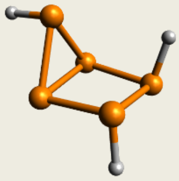

```
8
compound 19a-1 @ PBE0-D3/def2-TZVP
P           1.43947         1.10036         0.12936
P          -0.65556         1.15947        -0.65468
P          -0.59109        -1.07825        -0.76163
P           1.36559        -1.11265         0.31833
H           2.13450        -1.30169        -0.86204
H           1.16628         1.44132         1.48181
P          -1.58894        -0.07256         0.91180
H          -2.84262        -0.08511         0.23236
```

```
8
compound 19a-2 @ PBE0-D3/def2-TZVP
P          -0.35188         1.35530         1.13165
P          -0.35188        -0.88286         1.11524
P          -0.35188        -0.88286        -1.11524
P          -0.35188         1.35530        -1.13165
H          -1.76427         1.43691        -1.25370
H          -1.76427         1.43691         1.25370
P           1.53982        -0.97647        -0.00000
H           1.54401        -2.40010        -0.00000
```

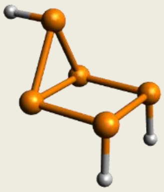

```
8
compound 19a-3 @ PBE0-D3/def2-TZVP
P          -0.41574         1.39301         1.11879
P          -0.41574        -0.85060         1.12308
P          -0.41574        -0.85060        -1.12308
P          -0.41574         1.39301        -1.11879
H           0.96946         1.58122        -1.37420
H           0.96946         1.58122         1.37420
P           1.44755        -1.12597         0.00000
H           1.29214        -2.54507         0.00000
```

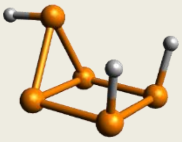

```
8
compound 19a-4 @ PBE0-D3/def2-TZVP
P          -0.35516         1.35182         1.13103
P          -0.35516        -0.88026         1.11890
P          -0.35516        -0.88026        -1.11890
P          -0.35516         1.35182        -1.13103
H          -1.76692         1.46295        -1.25298
H          -1.76692         1.46295         1.25298
P           1.52602        -1.15196        -0.00000
H           1.95330         0.20685        -0.00000
```

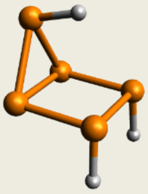

```
8
compound 19b-1 @ PBE0-D3/def2-TZVP
P           0.00178         1.54377         1.00408
P           0.00178         1.54377        -1.00408
P           0.00178        -0.52909         1.75008
P           0.00178        -0.52909        -1.75008
P          -0.09389        -1.83216        -0.00000
H           1.40612        -0.54937        -1.96453
H          -1.51056        -1.85912        -0.00000
H           1.40612        -0.54937         1.96453
```

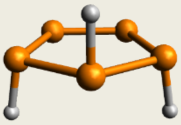

```
8
compound 19b-2 @ PBE0-D3/def2-TZVP
P        -1.40400        -1.17849         0.07914
P        -1.62762         0.82065         0.17237
P         0.76967        -1.62184        -0.18951
P         0.29577         1.64729        -0.42690
P         1.79241         0.23820         0.32142
H         0.41768         2.65480         0.56549
H         1.39417         0.17710         1.68445
H         0.79458        -1.41909        -1.59772
```

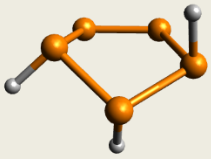

```
8
compound 19b-3 @ PBE0-D3/def2-TZVP
P        -0.29986         1.48081         1.00909
P        -0.29986         1.48081        -1.00909
P        -0.29986        -0.65176         1.54262
P        -0.29986        -0.65176        -1.54262
P         0.97570        -1.54237         0.00000
H         0.66569        -0.57852        -2.57905
H         2.02468        -0.57900         0.00000
H         0.66569        -0.57852         2.57905
```

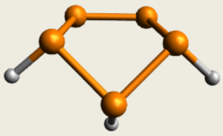

```
8
compound 19c @ PBE0-D3/def2-TZVP
P         0.00000         1.71865         0.00000
P         1.48840        -0.85933         0.00000
P        -1.48840        -0.85933         0.00000
P         0.00000         0.00000         1.43917
P         0.00000         0.00000        -1.43917
H        -2.44810         0.20262         0.00000
H         1.39953         2.01881         0.00000
H         1.04858        -2.22143         0.00000
```

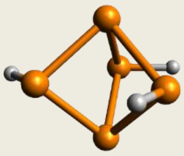

7 References

[1] C. B. Fischer, S. Xu, H. Zipse, *Chem. Eur. J.* **2006**, *12*, 5779–5784.

[2] J. Bresien, A. Hinz, A. Schulz, T. Suhrbier, M. Thomas, A. Villinger, *Chem. Eur. J.* **2017**, *23*, 14738–14742.

[3] J. Bresien, K. Faust, A. Schulz, A. Villinger, *Angew. Chem. Int. Ed.* **2015**, *54*, 6926–6930.

[4] U. Rosenthal, V. V. Burlakov, P. Arndt, W. Baumann, A. Spannenberg, *Organometallics* **2003**, *22*, 884–900.

[5] M. Kuprat, M. Lehmann, A. Schulz, A. Villinger, *Organometallics* **2010**, *29*, 1421–1427.

[6] P. H. M. Budzelaar, *gNMR for Windows*, IvorySoft, **2006**.

[7] G. M. Sheldrick, *SHELXTL-2014: Program for the Solution of Crystal Structures*, University of Göttingen, Germany, **2014**.

[8] G. M. Sheldrick, *SHELXL-2013: Program for the Refinement of Crystal Structures*, University of Göttingen, Germany,**2013**.

[9] G. M. Sheldrick, *SADABS Version 2*, University of Göttingen, Germany, **2004**.

[10] A. H. Cowley, N. C. Norman, M. Pakulski, G. Becker, M. Layh, E. Kirchner, M. Schmidt, *Inorg. Synth.* **1990**, *27*, 235–240.

[11] M. Yoshifuji, I. Shima, N. Inamoto, K. Hirotsu, T. Higuchi, *J. Am. Chem. Soc.* **1981**, *103*, 4587–4589.

[12] M. Yoshifuji, I. Shima, N. Inamoto, K. Hirotsu, T. Higuchi, *J. Am. Chem. Soc.* **1982**, *104*, 6167.

[13] A. Hinz, R. Kuzora, U. Rosenthal, A. Schulz, A. Villinger, *Chem. Eur. J.* **2014**, *20*, 14659–14673.

[14] E. Niecke, R. Rüger, B. Krebs, *Angew. Chem. Int. Ed. Engl.* **1982**, *21*, 544–545.

[15] J. Bresien, K. Faust, C. Hering-Junghans, J. Rothe, A. Schulz, A. Villinger, *Dalton Trans.* **2016**, *45*, 1998–2007.

[16] A. Hinz, A. Schulz, A. Villinger, *J. Am. Chem. Soc.* **2015**, *137*, 9953–9962.

[17] *Gaussian 09, Revision E.01*, M. J. Frisch, G. W. Trucks, H. B. Schlegel, G. E. Scuseria, M. A. Robb, J. R. Cheeseman, G. Scalmani, V. Barone, B. Mennucci, G. A. Petersson, H. Nakatsuji, M. Caricato, X. Li, H. P. Hratchian, A. F. Izmaylov, J. Bloino, G. Zheng, J. L. Sonnenberg, M. Hada, M. Ehara, K. Toyota, R. Fukuda, J. Hasegawa, M. Ishida, T. Nakajima, Y. Honda, O. Kitao, H. Nakai, T. Vreven, J. A. Montgomery Jr., J. E. Peralta, F. Ogliaro, M. Bearpark, J. J. Heyd, E. Brothers, K. N.

Kudin, V. N. Staroverov, T. Keith, R. Kobayashi, J. Normand, K. Raghavachari, A. Rendell, J. C. Burant, S. S. Iyengar, J. Tomasi, M. Cossi, N. Rega, J. M. Millam, M. Klene, J. E. Knox, J. B. Cross, V. Bakken, C. Adamo, J. Jaramillo, R. Gomperts, R. E. Stratmann, O. Yazyev, A. J. Austin, R. Cammi, C. Pomelli, J. W. Ochterski, R. L. Martin, K. Morokuma, V. G. Zakrzewski, G. A. Voth, P. Salvador, J. J. Dannenberg, S. Dapprich, A. D. Daniels, O. Farkas, J. B. Foresman, J. V. Ortiz, J. Cioslowski, D. J. Fox, Gaussian, Inc., Wallingford CT, **2013**.

[18] F. Neese, *WIREs Comput. Mol. Sci.* **2018**, *8*, e1327.

[19] E. D. Glendening, J. K. Badenhoop, A. E. Reed, J. E. Carpenter, J. A. Bohmann, C. M. Morales, C. R. Landis, F. Weinhold, *NBO 6.0*, Theoretical Chemistry Institute, University of Wisconsin, Madison, **2013**.

[20] J. E. Carpenter, F. Weinhold, *J. Mol. Struct.: THEOCHEM* **1988**, *169*, 41–62.

[21] F. Weinhold, J. E. Carpenter, *The Structure of Small Molecules and Ions*, Plenum Press, **1988**.

[22] F. Weinhold, C. R. Landis, *Valency and Bonding. A Natural Bond Orbital Donor-Acceptor Perspective*, Cambridge University Press, **2005**.

[23] J. P. Perdew, K. Burke, M. Ernzerhof, *Phys. Rev. Lett.* **1996**, *77*, 3865–3868.

[24] J. P. Perdew, K. Burke, M. Ernzerhof, *Phys. Rev. Lett.* **1997**, *78*, 1396–1396.

[25] C. Adamo, V. Barone, *J. Chem. Phys.* **1999**, *110*, 6158–6170.

[26] S. Grimme, J. Antony, S. Ehrlich, H. Krieg, *J. Chem. Phys.* **2010**, *132*, 154104.

[27] S. Grimme, S. Ehrlich, L. Goerigk, *J. Comput. Chem.* **2011**, *32*, 1456–1465.

[28] F. Weigend, R. Ahlrichs, *Phys. Chem. Chem. Phys.* **2005**, *7*, 3297–305.

[29] I. M. Alecu, J. Zheng, Y. Zhao, D. G. Truhlar, *J. Chem. Theory Comput.* **2010**, *6*, 2872–2887.

[30] F. London, *J. Phys. Radium* **1937**, *8*, 397–409.

[31] R. McWeeny, *Phys. Rev.* **1962**, *126*, 1028–1034.

[32] R. Ditchfield, *Mol. Phys.* **1974**, *27*, 789–807.

[33] K. Wolinski, J. F. Hinton, P. Pulay, *J. Am. Chem. Soc.* **1990**, *112*, 8251–8260.

[34] J. R. Cheeseman, G. W. Trucks, T. A. Keith, M. J. Frisch, *J. Chem. Phys.* **1996**, *104*, 5497–5509.

[35] C. J. Jameson, A. De Dios, A. Keith Jameson, *Chem. Phys. Lett.* **1990**, *167*, 575–582.

[36] C. van Wüllen, *Phys. Chem. Chem. Phys.* **2000**, *2*, 2137–2144.

[37] W. Deng, J. R. Cheeseman, M. J. Frisch, *J. Chem. Theory Comput.* **2006**, *2*, 1028–1037.

[38] C. J. Cramer, B. A. Smith, *J. Phys. Chem.* **1996**, *100*, 9664–9670.

[39] C. J. Cramer, *Essentials of Computational Chemistry: Theories and Models*, John

Wiley & Sons, Ltd, Chichester, UK, **2004**.

[40] J. Bresien, T. Kröger-Badge, S. Lochbrunner, D. Michalik, H. Müller, A. Schulz, E. Zander, *Chem. Sci.* **2019**, *10*, 3486–3493.

[41] C. Riplinger, F. Neese, *J. Chem. Phys.* **2013**, *138*, 034106.

[42] D. G. Liakos, M. Sparta, M. K. Kesharwani, J. M. L. Martin, F. Neese, *J. Chem. Theory Comput.* **2015**, *11*, 1525–1539.

[43] C. Riplinger, P. Pinski, U. Becker, E. F. Valeev, F. Neese, *J. Chem. Phys.* **2016**, *144*, 024109.

[44] D. Hegarty, M. A. Robb, *Mol. Phys.* **1979**, *38*, 1795–1812.

[45] R. H. A. Eade, M. A. Robb, *Chem. Phys. Lett.* **1981**, *83*, 362–368.

[46] H. B. Schlegel, M. A. Robb, *Chem. Phys. Lett.* **1982**, *93*, 43–46.

[47] P. E. M. Siegbahn, *Chem. Phys. Lett.* **1984**, *109*, 417–423.

[48] F. Bernardi, A. Bottoni, J. J. W. McDouall, M. A. Robb, H. B. Schlegel, *Faraday Symp. Chem. Soc.* **1984**, *19*, 137.

[49] M. A. Robb, U. Niazi, in *Reports in Molecular Theory, Vol. 1* (Eds.: H. Weinstein, G. Náray-Szabó), CRC Press, Boca Raton, FL, **1990**, pp. 23–55.

[50] M. Frisch, I. N. Ragazos, M. A. Robb, H. B. Schlegel, *Chem. Phys. Lett.* **1992**, *189*, 524–528.

[51] N. Yamamoto, T. Vreven, M. A. Robb, M. J. Frisch, H. B. Schlegel, *Chem. Phys. Lett.* **1996**, *250*, 373–378.

[52] M. Klene, M. A. Robb, M. J. Frisch, P. Celani, *J. Chem. Phys.* **2000**, *113*, 5653–5665.

[53] L. Salem, C. Rowland, *Angew. Chem. Int. Ed. Engl.* **1972**, *11*, 92–111.

[54] E. Miliordos, K. Ruedenberg, S. S. Xantheas, *Angew. Chem. Int. Ed.* **2013**, *52*, 5736–5739.
